# Drugtamer‐PROTAC Conjugation Strategy for Targeted PROTAC Delivery and Synergistic Antitumor Therapy

**DOI:** 10.1002/advs.202401623

**Published:** 2024-04-19

**Authors:** Shipeng He, Yuxin Fang, Yaojin Zhu, Ziyang Ma, Guoqiang Dong, Chunquan Sheng

**Affiliations:** ^1^ Center for Basic Research and Innovation of Medicine and Pharmacy (MOE) School of Pharmacy Second Military Medical University (Naval Medical University) 325 Guohe Road Shanghai 200433 P. R. China; ^2^ Institute of Translational Medicine Shanghai University 99 Shangda Road Shanghai 200444 P. R. China

**Keywords:** drug combination, drugtamer, fluorouracil, NAMPT, PROTAC

## Abstract

Proteolysis‐targeting chimeras (PROTACs) have emerged as a promising strategy for targeted protein degradation and drug discovery. To overcome the inherent limitations of conventional PROTACs, an innovative drugtamer‐PROTAC conjugation approach is developed to enhance tumor targeting and antitumor potency. Specifically, a smart prodrug is designed by conjugating “drugtamer” to a nicotinamide phosphoribosyltransferase (NAMPT) PROTAC using a tumor microenvironment responsible linker. The “drugtamer” consists of fluorouridine nucleotide and DNA‐like oligomer. Compared to NAMPT PROTAC and the combination of PROTAC + fluorouracil, the designed prodrug **AS‐2F‐NP** demonstrates superior tumor targeting, efficient cellular uptake, improved in vivo potency and reduced side effects. This study provides a promising strategy for the precise delivery of PROTAC and synergistic antitumor agents.

## Graphical abstract

The first drugtamer‐PROTAC conjugate, which enhances tumor targeting and efficacy in vivo while reducing side effects, is developed by linking a nucleolin‐specific aptamer with nicotinamide phosphoribosyltransferase (NAMPT) PROTAC and fluorouridine nucleotides through a drug‐constituted DNA‐like oligomer (drugtamer) and a tumor microenvironment‐responsive linker. This approach may revolutionize the precise delivery of PROTAC and synergistic cancer treatments.

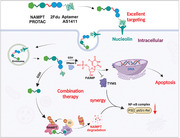

## 1 Introduction

Proteolysis‐targeting chimeras (PROTACs) are gaining popularity as a promising technology for drug discovery and development.^[^ ^1^ ^]^ These bifunctional molecules consist of a target protein‐ligand, linker, and E3 ligase ligand. By recruiting an E3 ligase in proximity to the target protein, PROTACs facilitate the formation of ternary complexes, inducing the ubiquitination of the target protein and its subsequent degradation by the proteasome. Compared with conventional small‐molecule inhibitors, PROTACs exhibit remarkable advantages in catalytic degradation at low dosages and selectively eliminate obstinate proteins.^[^ ^1^, ^2^ ^]^ Nevertheless, drug development for PROTACs faces several major challenges, including non‐specific tumor targeting, limited in vivo potency, and significant toxicity.^[^ ^3^, ^4^ ^]^

To improve the therapeutic efficacy of PROTACs, combinations of PROTAC and small‐molecule antitumor agents are currently being evaluated in clinical trials. For example, the estrogen receptor (ER) PROTAC ARV‐471, in combination with everolimus (ClinicalTrials.gov identifier: NCT05501769) or palbociclib (ClinicalTrials.gov identifier: NCT04072952), has been investigated for the treatment of breast cancer.^[^ ^5^, ^6^, ^7^, ^8^ ^]^ The clinical efficacy of the androgen receptor (AR) PROTAC ARV‐110 and abiraterone in prostate cancer is currently in progress (ClinicalTrials.gov Identifier: NCT05177042).^[^ ^9^, ^10^ ^]^ However, owing to the unique pharmacokinetic properties of individual drugs, traditional “cocktail” combination therapies face challenges in achieving balanced drug delivery to tumor sites, thereby escalating the risk of potential toxicity.^[^ ^11^, ^12^, ^13^, ^14^, ^15^ ^]^ Therefore, there is an imperative need to develop novel strategies to improve the efficiency of PROTAC‐based combination therapies, particularly those capable of precisely modulating drug accumulation in tumors to enhance synergistic therapeutic efficacy.

Aptamers, also known as chemical antibodies, possess great potential as targeting moieties because of their low immunogenicity, facile synthesis, potent tissue penetration, and high binding affinity for specific receptors.^[^ ^16^, ^17^ ^]^ Previously, we developed an aptamer‐PROTAC conjugation strategy by combining the aptamer AS1411 (**AS**) with a bromodomain and extra‐terminal domain (BET) PROTAC through a glutathione‐responsive linker.^[^ ^18^ ^]^ The resulting conjugate showed improved targeting ability in breast cancer cells. However, such aptamer‐drug conjugates typically face limitations in their drug‐loading capacity, making it challenging to sequentially conjugate multiple drug molecules onto aptamers.^[^ ^19^ ^]^ To address this bottleneck, we designed a novel multifunctional smart prodrug system, named drug tamer‐PROTAC conjugate, to simultaneously realize precise tumor targeting, efficient delivery, and synergistic combination therapy. Specifically, taking advantage of the structural similarity of fluorouridine nucleotides (**Fdu**) with thymidine (T) nucleotides, **Fdu** was directly integrated into nucleic acids during the solid‐phase synthesis of the aptamer, which was further attached to a synergistic PROTAC through a tumor microenvironment‐responsive linker. Therefore, improved tumor targeting, antitumor efficacy, and reduced side effects can be achieved. As a conceptual validation study, we designed a smart prodrug by conjugating aptamer **AS**, cytotoxic **Fdu**, and nicotinamide phosphoribosyltransferase (NAMPT) PROTAC. The PROTAC conjugate showed improved aqueous solubility and was selectively recognized and efficiently delivered into tumor cells to release synergistic **Fdu** and PROTAC, leading to excellent in vivo antitumor potency and low toxicity.

## 2 Results

### 2.1 Rational Design of AS‐2Fdu‐NAMPT PROTAC Conjugates

Fluoropyrimidines such as 5‐fluorouracil (**FU**) are intracellularly converted into active **Fdu**, thereby impeding DNA and RNA synthesis (Figure S1, Supporting Information).^[^ ^20^ ^]^ As a frontline chemotherapeutic agent, **FU** is used in various treatment regimens for breast cancer when co‐administered with other anticancer drugs.^[^ ^21^, ^22^ ^]^ NAMPT is the rate‐limiting enzyme in nicotinamide adenine dinucleotide (NAD^+^) biosynthesis and is a promising antitumor target.^[^ ^23^ ^]^ **FU** has been reported to exert synergistic effects with the NAMPT inhibitor FK866, which increases the chemosensitivity of cancer cells to **FU** by inhibiting cell proliferation and inducing apoptosis.^[^ ^24^, ^25^ ^]^

**Figure 1:**
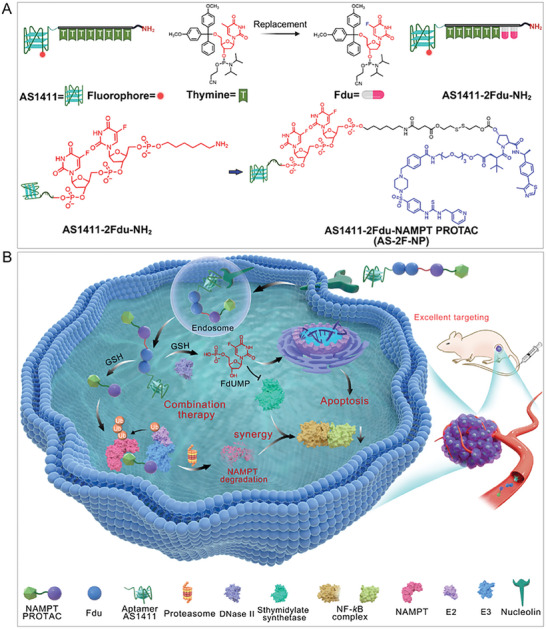
Design of drugtamer‐PROTAC conjugate **AS‐2F‐NP**. A) The chemical structures and design rationale of **AS‐2F‐NP**. The imaging molecules were designed by adding fluorophore in the aptamer. B) Schematic of the design strategy of drugtamer‐PROTAC conjugate **AS‐2F‐NP**. The aptamer segment selectively identifies the cell membrane receptor and is specifically internalized into tumor cells. The cleavable linker is susceptible to be attacked by GSH, leading to the release of the original **NP**. **AS‐2F‐NP** was activated by GSH and DNase II successively, then the FdUMP is released to block DNA synthesis and exerts synergistic antitumor effect with **NP**.

We previously designed a series of NAMPT PROTACs (herein referred to as **NP**).^[^ ^26^, ^27^, ^28^ ^]^ However, such NAMPT degraders are limited by their poor solubility and tumor tissue selectivity. Inspired by the synergism between **FU** and NAMPT inhibitors, we envisioned that the targeted co‐delivery of **FU** and **NP** would achieve enhanced antitumor activity and reduced side effects (**Figure** **1**).

**Figure 2:**
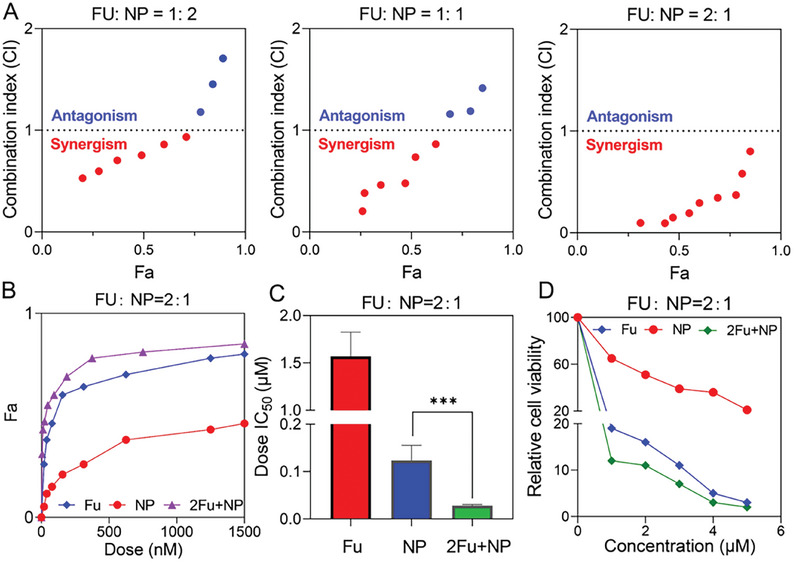
The synergistic effect of **FU** and **NP**. A) The combination index (CI) of **FU** with **NP** at different ratios. The best synergism was observed at a **FU**:**NP** ratio of 2:1. Fa represents fraction affected, which denotes the inhibition rate of cells. B) The dose–response curve for the combination therapy in the **FU**:**NP** ratio of 2:1. C,D) Comparative evaluation of cytotoxicity of **FU**, **NP**, and their 2:1 combination against MDA‐MB‐231 cells through the CCK‐8 assay.

Next, we validated the synergistic effects of **FU** and **NP** using Chou‐Talalay assays to determine the drug combination index (CI).^[^ ^29^ ^]^ Experiments were conducted at **FU:NP** ratios of 1:2, 1:1, and 2:1, respectively. The most pronounced synergism occurred at a ratio of 2:1 (Table S1, Supporting Information). When the cells were treated with different doses, the group with an **FU:NP** ratio of 2:1 exhibited synergism at all concentrations, indicating a stronger synergistic effect. In contrast, groups treated with **FU**: **NP** ratios of 1:1 or 1:2 showed synergistic effects (CI<1) at concentrations <0.5 µm. When the concentration increased, the antitumor efficacy improved accordingly, but the drug combinations tended to be antagonistic (CI >1) or additive (CI = 1) (**Figure** **2**A). The dose–response curves demonstrated that the differences were more significant in the 2:1 ratio than in the other combinations (Figure 2B). The cytotoxic effects of **FU**, **NP,** and their combination on breast cancer cells were evaluated using a cell counting Kit‐8 (CCK8) assay. The results showed that the combination treatment was more effective than the single drugs (Figure S2, Supporting Information), with the best inhibitory activity in cell viability observed at the **FU:NP** ratio of 2:1 (Figure 2C,D). Therefore, the ratio 2:1 was selected for the subsequent conjugate design. **AS** is widely recognized as a nucleolin receptor‐targeting aptamer with a specificity for breast cancer cells.^[^ ^18^ ^]^ Inspired by this synergism, a smart **AS**‐based prodrug for the targeted co‐delivery of **2Fdu** (the active form of **FU**) and **NP** was rationally designed. Considering the possibility that conjugated drugs may disrupt the targeting specificity of **AS** aptamer, we incorporated six thymine nucleotides at the **AS** terminus. Leveraging the structural similarity between **Fdu** and natural thymine nucleotides, we integrated two **Fdu** molecules into thymine nucleotides (**AS‐2Fdu‐NH_2_**) to accurately transport the drug payloads. **AS‐2Fdu‐NH_2_** can be conveniently prepared via solid‐phase synthesis. The **Fdu** integrated aptamer was further conjugated with **NP** through a tumor microenvironment‐responsive linker. Specifically, the hydroxyl group in the VHL ligand of **NP** was used as a suitable attachment point for linker extension. Although introducing a linker could potentially disrupt the VHL‐mediated recruitment of the E3 ubiquitin ligase and thereby reduce degradation activity, we designed a GSH‐sensitive carbonate disulfide cleavable linker to ensure the effective release of **NP**. Finally, the conjugate AS1411‐2Fdu‐NAMPT PROTAC (**AS‐2F‐NP**, **1**) was rationally designed (Figure 1A).

**Scheme 1:**
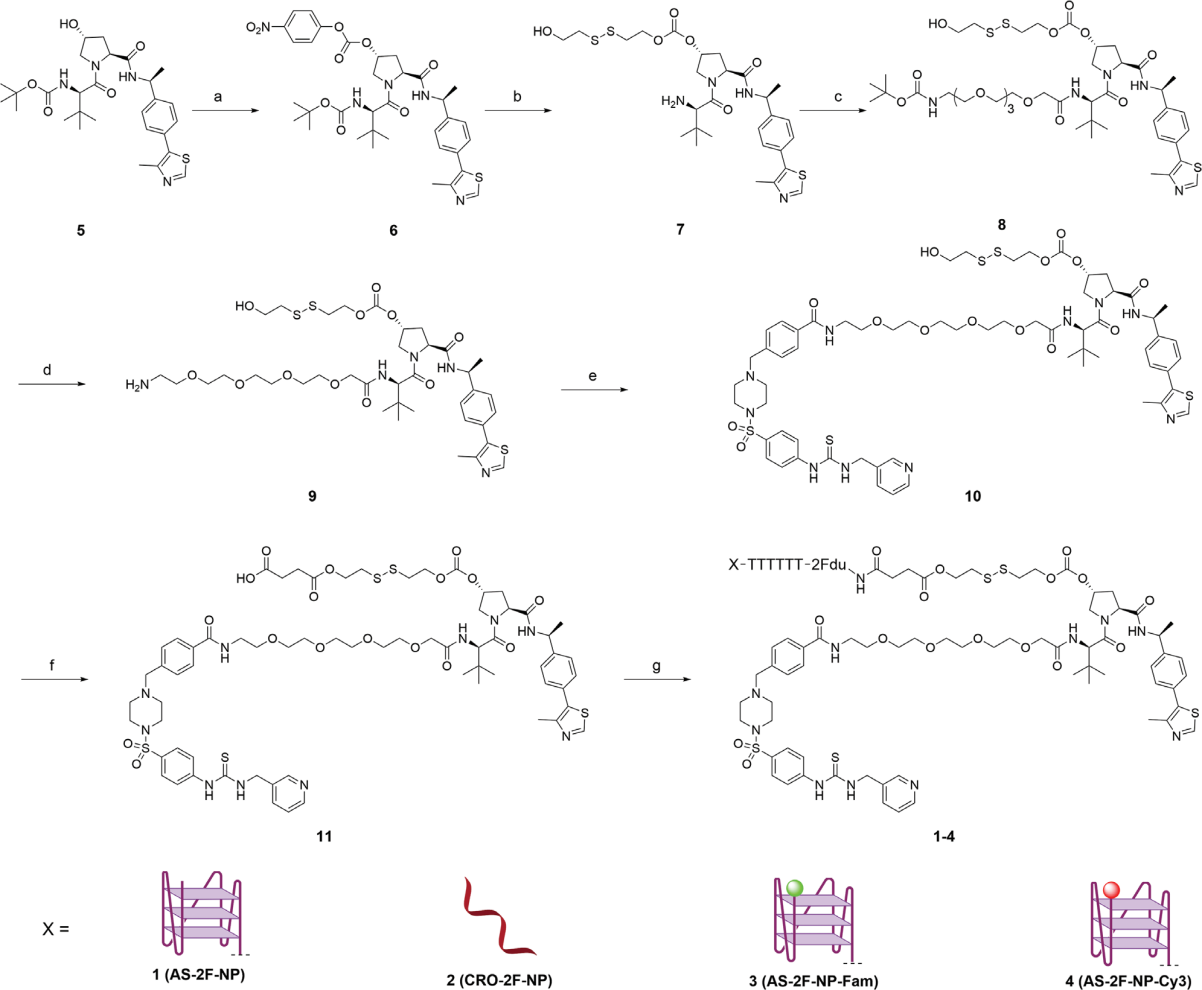
Reagents and conditions: a) 4‐nitrophenylchloroformate, DMAP, DCM, rt, 12 h, 47%; b) 2,2′‐disulfanediylbis(ethan‐1‐ol), DMAP, DCM, TFA, DCM, rt, 10 h, 44%; c) 2,2‐dimethyl‐4‐oxo‐3,8,11,14,17‐pentaoxa‐5‐azanonadecan‐19‐oic acid, HOBT, EDCI, DMF, rt, 5 h, 65%; d) TFA, DCM, rt, 2 h, 79%; e) compound **1S**, HOBT, EDCI, DMF, rt, 10 h, 56%; f) succinic anhydride, DMAP, DCM, rt, 3 h, 72%; g) aptamers, Sulfo‐NHS, EDCI, dd‐H_2_O, DMF, 0.5 m Na_2_CO_3_/NaHCO_3_, 12 h, 5–20%.

To further assess the tumor‐targeting capability and advantages of **AS‐2F‐NP**, we designed various control molecules (**Scheme** **1**), including non‐targeted **CRO‐2Fdu‐NP** (**CRO‐2F‐NP**, 2) and **AS‐2F‐NP** variants tagged with different fluorescent groups (**AS‐2F‐NP‐Fluorescein** (Fam), **3** and **AS‐2F‐NP‐Cyanine 3** (Cy3), **4**), for in vitro and in vivo evaluations.

### 2.2 Synthesis of Drugtamer‐PROTAC Conjugates

The synthesis of drugtamer‐PROTAC conjugates is outlined in Scheme 1. First, the hydroxyl group in the VHL ligand **5** was reacted with 4‐nitrophenyl carbonochloridate in the presence of DMAP to afford intermediate **6**. Key intermediate **7** was synthesized by reacting compound **6** with 2,2′‐disulfanediylbis(ethan‐1‐ol) under the catalytic reaction by DMAP. Compound **7** was condensed with dimethyl‐4‐oxo‐3,8,11,14‐tetraoxa‐5‐azahexadecan‐16‐oic acid in the presence of HOBT and EDCI to afford the intermediate **8**. Subsequently, the Boc protecting group was removed using TFA to yield compound **9**, which was further condensed with intermediate **1S** (Scheme S1, Supporting Information) in the presence of HATU and DIPEA to obtain compound **10**. Further esterification of **10** with succinic anhydride afforded the intermediate **11**. Finally, **11** was conjugated with **AS‐2Fdu‐NH_2_** in the presence of sulfo‐NHS and EDCI, resulting in the synthesis of the target compound **AS‐2F‐NP**. As controls, the CRO‐PROTAC conjugate (herein denoted as **CRO‐2F‐NP**) and Fam‐ or Cy3‐modified conjugates (hereafter denoted as **AS‐2F‐NP**‐**Fam**, **AS‐2F‐NP**‐**Cy3**, and **AS‐2F‐NP‐Cy3**) were synthesized using a protocol similar to that described for **AS‐2F‐NP**. The structures of the crucial intermediates were verified using ^1^H‐NMR, ^13^C‐NMR, and high‐resolution mass spectrometry (HRMS). The aptamer‐modified conjugates were purified using reversed‐phase high‐performance liquid chromatography (RP‐HPLC) and validated using mass spectrometry.

### 2.3 GSH‐Responsive Conjugate **AS‐2F‐NP** Demonstrated Favorable In Vitro Stability and Efficiently Released the Prototype PROTAC

**Figure 3:**
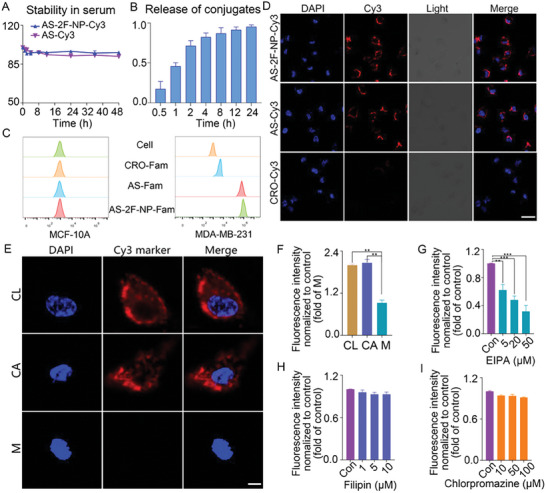
The stability, release, targeting, cellular uptake, and internalization of conjugate **AS‐2F‐NP**. A) The 10 µM concentrations of **AS‐Cy3** and **AS‐2F‐NP‐Cy3** were incubated for 48 h in a medium containing serum. B) HPLC of drug‐release of **AS‐2F‐NP** in the reducing conditions for 24 h, the Y axis represents relative amount of **NP**. C) MDA‐MB‐231 and MCF‐10A cells were individually subjected to flow cytometry after incubation with 500 nm Fam‐modified **AS**, **AS‐2F‐NP,** and **CRO** for 0.5 h at 45 °C. D) Confocal laser scanning micrographs of MDA‐MB‐231 cells were captured following a 2 h treatment with 500 nm Cy3‐modified **AS‐2F‐NP**, **AS**, and **CRO** in DMEM at 37 °C. The scale bar represents 20 µm. E) Illustrative confocal micrographs of MDA‐MB‐231 cells post‐pretreatment with inhibitors targeting endocytic pathways (CA, caveolae; CL, clathrin; M, macropinocytosis) and subsequent incubation with 500 nM Cy3‐modified **AS‐2F‐NP**. The scale bar denotes 10 µm. F–I) Chemical inhibition was employed to impede the cellular internalization of Cy3‐modified conjugates in MDA‐MB‐231 cells. Compared to the control experiment (no inhibitor, F), the relative fluorescence of Cy3 was assessed via flow cytometry after treatment with inhibitors targeting three endocytic pathways: EIPA (macropinocytosis, G), filipin (caveolae‐mediated endocytosis pathway, H), or chlorpromazine (clathrin pathway, I). The error bars represent the mean ± sd values; n = 3. The p values were calculated using ANOVA with Tukey's test. *p <0.1, **p <0.01, ***p <0.05.

The stability of **AS‐2F‐NP** was assessed using 10% polyacrylamide gel electrophoresis (PAGE) (**Figure** **3**A; Figure S3, Supporting Information). **AS‐Cy3** and **AS‐2F‐NP‐Cy3** were incubated in a medium containing 10% fetal bovine serum (FBS) at 37 °C for different durations. Similar to **AS‐Cy3**, **AS‐2F‐NP‐Cy3** demonstrated good stability after 48 h of incubation in a serum‐containing medium. **AS‐2F‐NP‐Cy3** mostly retained its intact form.

This suggests that the terminal modification of **AS** with **Fdu** and the PROTAC molecule did not compromise the degradation of **AS**. Thus, the conjugate **AS‐2F‐NP‐Cy3** possessed good in vitro stability. Additionally, to confirm effective NP release from **AS‐2F‐NP**, a 50 µM solution of **AS‐2F‐NP** was supplemented with an equivalent concentration of GSH to mimic the elevated glutathione environment in tumor tissues. As illustrated in Figure 3B, the **NP** concentration increased in a time‐dependent manner, and effective release from **AS‐2F‐NP** occurred within 2 h with a release rate of 63.7%. These findings validate the effective release of prototype **NP** from conjugate **AS‐2F‐NP** under reducing conditions.

### 2.4 Effects of Conjugate **AS‐2F‐NP** on Cellular Uptake and Internalization

To assess the uptake and internalization of **AS‐2F‐NP** by MDA‐MB‐231 cells, Fam‐ and Cy3‐modified conjugates were analyzed using flow cytometry and confocal laser scanning microscopy. **AS** specifically targets cell membrane‐localized nucleolin that is overexpressed in MDA‐MB‐231 breast cancer cells. Thus, MDA‐MB‐231 and MCF‐10A (normal human mammary epithelial) cells were selected as nucleolin‐positive and nucleolin‐negative cells, respectively. Flow cytometry demonstrated a notably elevated cellular uptake efficiency for both **AS‐Fam** and **AS‐2F‐NP‐Fam** compared to that of the non‐selective **CRO‐Fam** conjugates following incubation with MDA‐MB‐231 cells (Figure 3C). Conversely, no noticeable differences in the fluorescence intensities between Fam‐modified targeting and non‐targeting aptamers were observed during incubation with MCF‐10A control cells. These findings confirm the specific recognition of **AS‐2F‐NP**‐Fam by MDA‐MB‐231 cells. The uptake and specificity of **AS‐2F‐NP** were explored using confocal laser scanning microscopy. After treatment for 1 h with **AS‐2F‐NP‐Cy3**, **AS‐Cy3**, and **CRO‐Cy3**, MDA‐MB‐231 cells were stained with DAPI, and Fam‐modified samples were examined. As shown in Figure 3D, cells incubated with **AS‐2F‐NP‐Cy3** or **AS‐Cy3** exhibited stronger red fluorescence than those incubated with **CRO‐Cy3**, confirming the excellent and specific nucleolin‐mediated internalization of **AS‐2F‐NP**. Moreover, pretreatment of MDA‐MB‐231 cells with EIPA (a macropinocytosis inhibitor) resulted in a dose‐dependent decrease in the red fluorescence of **AS‐2F‐NP‐Cy3** (Figure 3E–G) compared with vehicle‐treated control cells. Conversely, Cy3 fluorescence signals were observed in the cytoplasm of MDA‐MB‐231 cells (Figure 3H,I) pretreated with various concentrations of chlorpromazine (a clathrin pathway inhibitor) or filipin (a caveolae‐mediated endocytosis pathway inhibitor). This difference indicated that **AS‐2F‐NP‐Cy3** primarily entered MDA‐MB‐231 cells through macropinocytosis. This intriguing rapid internalization mechanism may offer a novel strategy for enhancing the cell membrane permeability of synergistic drugs.

### 2.5 AS‐2F‐NP Specifically Degraded NAMPT in MDA‐MB‐231 Cells

**Figure 4:**
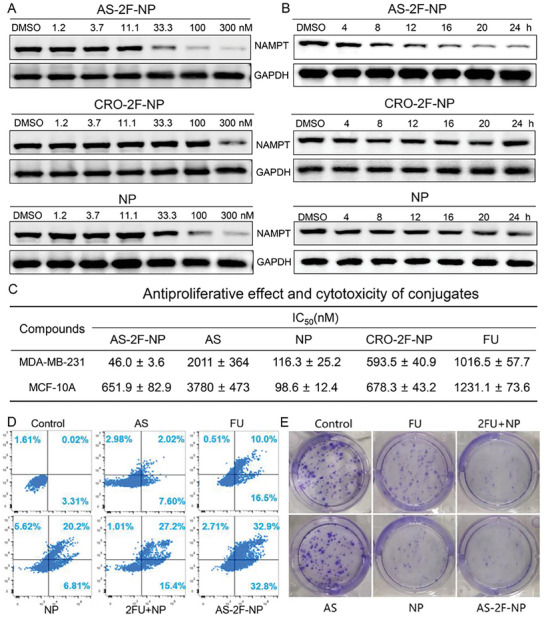
Degradation of NAMPT and cytotoxicity induced by the conjugates. A) Analysis using western blotting assessing the effects of **AS‐2F‐NP**, **CRO‐2F‐NP**, and **NP** compounds on NAMPT degradation in MDA‐MB‐231 cells after 24 h of treatment. The results of grayscale statistics are shown in Figure S4 (Supporting Information). B) Evaluation of the impact of **AS‐2F‐NP**, **CRO‐2F‐NP**, and **NP** on NAMPT degradation in MDA‐MB‐231 cells at different time points, with GAPDH serving as the loading control. The results of grayscale statistics are shown in Figure S4 (Supporting Information). C) Cytotoxicity of **AS‐2F‐NP**, **CRO‐2F‐NP**, **Fdu**, and **NP** in MDA‐MB‐231 nucleolin‐positive cells and MCF‐10A nucleolin‐negative cells. IC_50_ values are the mean of at least three independent assays, presented as the mean ± sd. D) Apoptosis of MDA‐MB‐231 cells in different treatment groups. E) Clone formation of target MDA‐MB‐231 cells treated with different formulations.

To further investigate the NAMPT degradation activity of **AS‐2F‐NP**, western blotting was performed on MDA‐MB‐231 cells stimulated with various concentrations of **AS‐2F‐NP** and **CRO‐2F‐NP** using **NP** as a control (**Figure** **4**A; Figure S4, Supporting Information). **AS‐2F‐NP** and **NP** caused concentration‐dependent degradation of NAMPT (**AS‐2F‐NP**: DC_50_ = 18 nm, *D*_max_ >90%; **NP**: DC_50_ = 70 nm, *D*_max_ >90%), whereas NAMPT degradation was significantly diminished in cells treated with **CRO‐2F‐NP** (DC_50_ = 133 nm). These results demonstrate the enhanced efficacy of **AS‐2F‐NP** in degrading NAMPT in nucleolin‐overexpressing MDA‐MB‐231 cells, indicating that **AS** addition improved the degradation activity compared to the original **NP**. In addition, the time‐dependent degradation activity of **AS‐2F‐NP** was examined (Figure 4B). In MDA‐MB‐231 cells, **AS‐2F‐NP** and **NP** showed degradation activity at 12 and 16 h, respectively, whereas **CRO‐2F‐NP** exhibited no significant degradation activity at 24 h. The earlier onset of degradation observed with **AS‐2F‐NP** may be attributed to their enhanced cellular uptake, suggesting a more efficient internalization process. In MCF‐10A cells, **NP** maintained robust NAMPT degradation activity at 33.3 nm (Figure S5, Supporting Information). Conversely, **AS‐2F‐NP** and **CRO‐2F‐NP** only displayed weak degradation activity at 300 nm, possibly because of the lack of specificity and larger molecular size impeding cellular entry into MCF‐10A cells (Figure S5, Supporting Information). These findings highlighted the selective tumor cell‐targeting efficacy of **AS‐2F‐NP**.

### 2.6 **AS‐2F‐NP** Possessed Higher Cytotoxicity in MDA‐MB‐231 Cells than FU, NP, and Their Combination

The antiproliferative activity of **AS‐2F‐NP** was assessed using the CCK8 assay (Figure 4C; Figure S6, Supporting Information) using **NP** as a positive control, and all compounds were treated for 72 h. In MDA‐MB‐231 cells, **AS‐2F‐NP** (IC_50_ = 46 nm) demonstrated superior in vitro antiproliferative activity compared to **FU** (IC_50_ = 1016 nm) and **NP** (IC_50_ = 116 nm). At the same concentration gradient, the inhibitory rate of **AS‐2F‐NP** was similar to that of the combination of **2FU** and **NP** (Figure S7, Supporting Information). In contrast, **CRO‐2F‐NP** (IC_50_ = 593 nm) and **AS** (IC_50_ = 2.01 µm) exhibited significantly lower antiproliferative activity than **AS‐2F‐NP**. In MCF‐10A cells, the cytotoxicity of **AS‐2F‐NP** was substantially reduced (IC_50_ = 652 nM), which was markedly lower than **NP** (IC_50_ = 98.6 nm) and comparable to that of **CRO‐2F‐NP** (IC_50_ = 678 nm). In addition, **AS** exhibited weak cytotoxicity against MCF‐10A cells within the tested concentration range (IC_50_ = 3.8 µm). These findings are consistent with the reported results that AS‐based conjugates exhibit significantly lower toxicity toward normal cells than toward breast cancer cells.^[^ ^30^, ^31^ ^]^ These results suggested that the designed **AS‐2F‐NP** exhibited excellent targeting capabilities toward nucleolin‐positive MDA‐MB‐231 cells. The apoptotic effects of **AS‐2F‐NP** on MDA‐MB‐231 cells were assessed (Figure 4D). After 48 h of treatment with 100 nM **AS‐2F‐NP**, **NP**, **FU**, or **2FU+NP**, the apoptosis rates in MDA‐MB‐231 cells were 68.4%, 32.6%, 27%, and 43.6%, respectively. Under the same conditions, the apoptotic rate of **AS**‐treated cells was 12.6%, which was significantly lower than that of cells treated with **AS‐2F‐NP**. The effects on apoptosis were consistent with the antiproliferative activity in MDA‐MB‐231 cells. A colony formation assay was conducted to investigate the antiproliferative effects on MDA‐MB‐231 cells (Figure 4E). **AS‐2F‐NP**, **NP**, **FU**, and **2FU+NP** effectively inhibit the growth of MDA‐MB‐231 cells. Among them, **AS‐2F‐NP** exhibited the most pronounced inhibitory effect, indicating enhanced selectivity toward nucleolin‐overexpressing MDA‐MB‐231 cells. These results highlighted the potential of **AS‐2F‐NP** as targeted therapeutic agents for cancer treatment, especially in cells with elevated nucleolin expression. **AS‐2F‐NP** exhibited enhanced NAMPT degradation activity and cytotoxicity compared with **NP**, **FU**, or their combination.

### 2.7 Proteome Level Analysis

**Figure 5:**
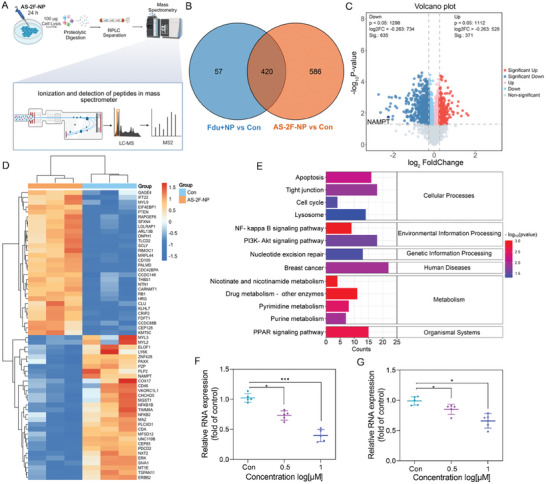
Proteome‐level analysis of **AS‐2F‐NP** treatment in MDA‐MB‐231 cells. A) MDA‐MB‐231 cells were incubated with **AS‐2F‐NP** and **2FU+NP** for 24 h, followed by processing cell lysates according to standard procedures for proteomic analysis. B) Venn diagram comparing differentially expressed proteins in the cell lysate of MDA‐MB‐231 cells treated with **AS‐2F‐NP** and PBS‐treated control. C) Volcano plot illustrating changes in differentially expressed proteins in the cell lysate of MDA‐MB‐231 cells treated with **AS‐2F‐NP** compared to the PBS‐treated control (n = 3; fold change >1.5; *p* <0.05). Colored points represent proteins that are significantly decreased (blue), increased (red), and unchanged (gray), respectively. D) Heatmap analysis of representative differentially expressed proteins in the cell lysate of MDA‐MB‐231 cells treated with **AS‐2F‐NP** compared to the PBS‐treated control (n = 3; *p* <0.05). E) KEGG analysis revealing different signaling pathways influenced by 1 µM **AS‐2F‐NP** in MDA‐MB‐231 cells. Quantitative analysis of p65 F) and ErK G) mRNA expression in MDA‐MB‐231 cells treated with **AS‐2F‐NP**, as determined using RT‐qPCR. The error bars represent the mean ± sd; n = 3. The p values were calculated by ANOVA with Tukey's test. *p <0.1, **p <0.01, ***p <0.05.

To further clarify the synergistic antitumor mechanism of **AS‐2F‐NP**, whole‐cell proteomic experiments were conducted using MDA‐MB‐231 cells (**Figure** **5**A). The results showed that **AS‐2F‐NP** interfered with the expression of 1006 genes, significantly larger than that of the 477 genes in the combined group of **FU** and **NP**. Notably, 420 genes in the **AS‐2F‐NP** intervention group were identical to those in the **FU** and **NP** combination groups (Figure 5B). To elucidate the impact of **AS‐2F‐NP** on cellular signaling pathways, we analyzed the relevant pathways and protein expression. Volcano plot and heatmap analyses revealed the differential expression of 635 downregulated and 371 upregulated proteins following **AS‐2F‐NP** treatment, among which NAMPT was significantly downregulated (Figure 5C,D; Figure S8A, Supporting Information). KEGG pathway enrichment analysis revealed the involvement of these affected proteins in several key biological pathways (Figure 5E; Figure S8B, Supporting Information). Notably, within the NAMPT‐involved pathway, a downregulation trend was observed for nicotinamide (Figure S9, Supporting Information). In the drug metabolism pathway, the metabolism of **FU** led to the downregulation of thymidine phosphorylase (TYMP) and five other genes (Figure S10, Supporting Information). Additionally, significant gene expression differences were observed between **AS‐2F‐NP** and PBS‐treated cells in the MAPK signaling pathway, with five genes markedly downregulated and four genes upregulated (Figure S11, Supporting Information). The representative genes p65 and ErK were selected as candidate genes, and their regulation was confirmed through RT‐qPCR and aligned using KEGG analysis. This consistency suggested that the expression trends of p65 (Figure 5F) and ErK (Figure 5G) were in agreement with the pathway‐level outcomes. These findings suggested that **AS‐2F‐NP** could serve as a pivotal regulatory agent with promising therapeutic potential for breast cancer treatment. Considering its NAMPT degradation and antiproliferative activities, **AS‐2F‐NP** was further subjected to in vivo assessment of its antitumor mechanisms and activities.

### 2.8 Conjugate **AS‐2F‐NP** Showed Excellent In Vivo Tumor‐Targeting Capability

**Figure 6:**
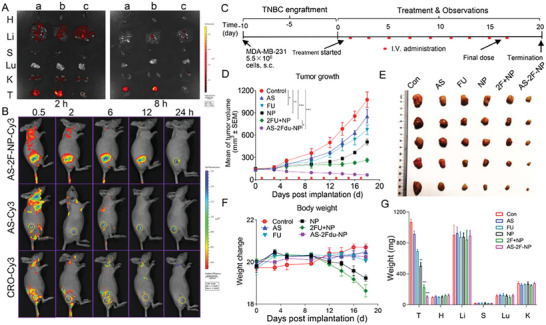
Distribution and antitumor efficacy of conjugates in MDA‐MB‐231 xenografts mice in vivo. A,B) Distribution results of **AS‐Cy3**, **AS‐2F‐NP‐Cy3**, and **CRO‐Cy3** in major organs (H, heart; Li, liver; S, spleen; L, lung; and K, kidney) and the tumor (T) at 2 h and 8 h after intravenous injection (a: **AS‐Cy3**, b: **AS‐2F‐NP‐Cy3**, c: **CRO‐Cy3**). C) Schematic of the procedure for in vivo experimental studies. D) Changes in tumor volume were recorded every other day during the 19‐day period of intravenous injection therapy, with red patches indicating the injection time points. Six groups were treated with intravenous injections of PBS, **AS** (10 µm kg^−1^), **FU** (1.3 mg kg^−1^), **NP** (11.8 mg kg^−1^), **2FU+NP** (2.6 mg kg^−1^+11.8 mg kg^−1^), and **AS‐2F‐NP** (10 µm kg^−1^), with each mice receiving 100 µL per injection. The doses of **FU** and **NP** were equivalent to 10 µm kg^−1^. Data are expressed as the mean ± sd. *p <0.05 and ***p <0.001, determined with one‐way ANOVA test for significance. E) Representative images depicting the size effects of xenograft tumors in each group after 19 days of treatment. F) Changes in body weight of mice were recorded every day during the 19‐day treatment period. G) On the last day of the animal experiment, mice from all groups were euthanized, major organs were harvested and weighed, and the weights of tumors and organs were recorded and analyzed.

To assess the tumor‐targeting characteristics of **AS‐2F‐NP**, imaging assays were employed to examine the tissue distribution of **AS‐2F‐NP‐Cy3** in MDA‐MB‐231 xenograft mice, with **AS‐Cy3** and **CRO‐Cy3** serving as controls. Following intravenous administration, the distribution of **AS‐Cy3**, **AS‐2F‐NP‐Cy3**, and **CRO‐Cy3** in the major organs (heart, liver, spleen, lung, and kidney) and tumors was examined (**Figure** **6**A). Following 2 h post‐administration, the fluorescence intensity of Cy3 in the tumor tissues of mice treated with **AS‐2F‐NP‐Cy3** was markedly higher than that observed in mice treated with **AS‐Cy3**. Conversely, minimal Cy3 fluorescence was detected in tumor tissues of mice treated with **CRO‐Cy3**. Additionally, all three compounds were slightly distributed in the liver and kidneys. After 8 h, the fluorescence intensity of Cy3 in the tumor tissues of mice treated with **AS‐2F‐NP‐Cy3** remained higher than that in **AS‐Cy3**‐treated mice, with no observable Cy3 fluorescence in the tumor tissues of mice treated with **CRO‐Cy3**. Furthermore, virtually no fluorescence signal was detected in the heart, spleen, or lungs in all three groups of mice. To further confirm the tumor‐targeting capability of the **AS‐2F‐NP** conjugate in MDA‐MB‐231 xenografts, we assessed the in vivo distribution and aggregation of **AS‐2F‐NP‐Cy3** in mice (Figure 6B). Remarkably, **AS‐2F‐NP‐Cy3** demonstrated the ability to recognize MDA‐MB‐231 tumor cells and aggregate in tumors because of the prolonged fluorescence retention time of **AS‐2F‐NP‐Cy3** in tumor tissues compared to **CRO‐Cy3** and **AS‐Cy3**. Furthermore, robust fluorescence signals were still observable in tumors at 12 h post‐injection, whereas no fluorescence signal was detected in the tumors of mice treated with **CRO‐Cy3**. **AS‐Cy3** group presented little accumulation in the tumor 2 h after iv administration. However, strong Cy3 fluorescence was observed even after 8 h (Figure 6A). These disparities may arise from variations in the pharmacokinetics of **AS‐Cy3** across experiments and time points. These findings confirmed the outstanding tumor‐targeting capability of **AS‐2F‐NP** in the MDA‐MB‐231 xenograft model.

### 2.9 Conjugate AS‐2F‐NP Showed Improved In Vivo Antitumor Potency by Diffusion of PROTAC within Tumor Tissues

**Figure 7:**
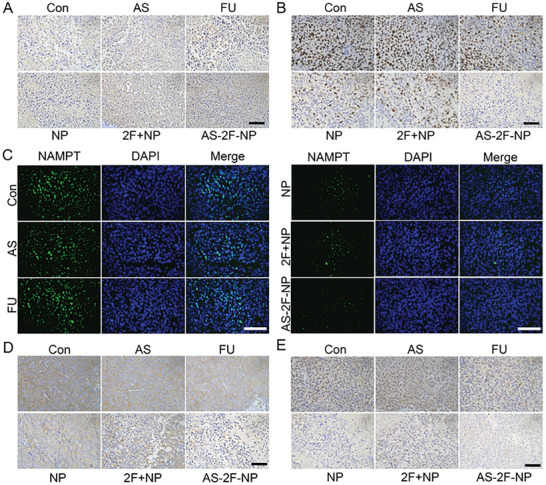
Immunohistochemistry and immunofluorescence of tumor tissues from different experimental groups. A) Immunohistochemistry results of tunnel staining on tumor tissue sections from the five treatment groups and the control group. Brown color is the result of tunnel staining. B) Micrographs of tumor tissue sections with Ki67 staining from the five treatment groups and the control group. Brown color is the result of Ki67 staining. C) Immunofluorescence results of NAMPT staining on tumor tissue sections from the five treatment groups and the control group. D) Micrographs of tumor tissue sections with ErK staining immunohistochemistry from the five treatment groups and the control group. E) Immunohistochemistry results of p65 staining on tumor tissue sections from the five treatment groups and the control group. Scale bar, 200 µm.

Considering the promising targeted antitumor activity of the **AS‐2F‐NP**, we evaluated their in vivo efficacy in a mouse MDA‐MB‐231 xenograft model. (Figure 6C). Upon reaching an average tumor volume of 150 mm^3^, the mice were randomly assigned to six groups. Each mouse received intravenous injections every other day, with a volume of 100 µL per injection. The injected substances included PBS, **AS** (10 µm kg^−1^), **FU** (1.3 mg kg^−1^), **NP** (11.8 mg kg^−1^), 2**FU**+**NP** (2.6 mg kg^−1^+11.8 mg kg^−1^), and **AS‐2F‐NP** (10 µm kg^−1^). The combined administration of 2**FU** and **NP** achieved a tumor growth inhibition (TGI) rate of 78.8% after 19 days, which was superior to that of **NP** (TGI = 60.1%) and **FU** (TGI = 44.2%) (Figure 6D,E). In contrast, **AS** exhibited negligible antitumor activity in vivo (TGI = 30.7%). Notably, **AS‐2F‐NP** demonstrated the best in vivo antitumor efficacy with a TGI >92%, highlighting the therapeutic advantages of the **AS‐2F‐NP** conjugation strategy. Throughout the treatment duration, the body weights of the treated mice were measured every 3 days to evaluate potential toxicity. The **AS**, **FU**, and **AS‐2F‐NP** groups exhibited favorable tolerability without significant weight loss or adverse reactions, whereas the **NP** and 2**FU**+**NP** groups exhibited toxicity, as evidenced by obvious weight loss (Figure 6F). Additionally, the weights of the vital organs of the treated mice were assessed. (Figure 6G), only the 2**FU**+**NP** group exhibited weight loss in the liver and lungs, whereas no significant differences were observed in the other groups compared to the control group. Toxicity was further evaluated using H&E staining, and lung damage was detected in mice treated with **NP** and **2FU**+**NP**, consistent with our previous findings^[^ ^18^, ^32^ ^]^ (Figure S12, Supporting Information). In contrast, mice treated with **AS‐2F‐NP** did not exhibit any signs of organ damage. These findings indicated that **AS** modification significantly reduced the toxicity of **NP** and **FU**. Furthermore, the in vivo antitumor mechanisms of **AS‐2F‐NP** were investigated using immunohistochemistry. These results indicate that **AS‐2F‐NP** was more active in inducing apoptosis than **Fdu**, **NP**, or the **2FU**+**NP** combination (**Figure** **7**A).

In the **AS‐2F‐NP** group, the number of TUNEL‐positive cells was significantly higher than those in the control, **AS**, **FU**, **NP**, and 2**FU**+**NP** treatment groups, indicating the superior apoptotic efficacy of **AS‐2F‐NP**. Immunohistochemistry revealed a significant decrease in the number of Ki67‐stained cells in the **AS‐2F‐NP** group compared to that in the control, **AS**, **FU**, **NP**, and 2**FU**+**NP** treatment groups, indicating that **AS‐2F‐NP** had a better inhibitory effect on cell proliferation (Figure 7B). To further explore in vivo NAMPT degradation efficiency, immunofluorescence, and immunohistochemical staining were performed using anti‐NAMPT antibodies. In contrast to the control group, the **AS** and **FU** groups showed no change in green fluorescence, the **NP** and **2FU**+**NP** groups exhibited a slight reduction, while green fluorescence was nearly undetectable in the **AS‐2F‐NP** treatment group, indicating lower NAMPT expression levels (Figure 7C). Immunohistochemistry results also suggested that **AS‐2F‐NP** was more effective in degrading NAMPT than **NP** and **2FU**+**NP**, leading to better in vivo antitumor efficacy (Figure S13, Supporting Information). These findings demonstrated that **AS** modification enhanced the degradation efficiency of NAMPT PROTAC in mice. Additionally, to further verify whether the combination of **NP** and **FU** synergistically decreased p65 (Figure 7D) and ErK (Figure 7E) expression, protein expression, and immunohistochemistry were conducted on tumor sections from all groups. Compared to the control group, there was no significant change in p65 and ErK proteins in the **AS** and **FU** groups, whereas the 2**FU**+**NP** group showed a notable enhancement and **AS‐2F‐NP** induced the strongest downregulation of p65 and ErK proteins. These findings indicate that **AS** modification further enhances the synergistic efficacy of the combined treatment with **NP** and **FU** in mice.

## 3 Discussion

The drugtamer‐PROTAC conjugation strategy represents a significant advancement in addressing the limitations of conventional PROTACs. By conjugating a nucleolin‐specific aptamer with NAMPT PROTAC and a fluorouridine nucleotide, the smart prodrug **AS‐2F‐NP** demonstrated superior tumor targeting, efficient cellular uptake, improved in vivo potency, and reduced toxicity, thereby providing a promising strategy for the precise delivery of PROTAC and synergistic antitumor agents.

Recently, several types of PROTAC prodrugs have been developed.^[^ ^33^, ^34^, ^35^, ^36^, ^37^, ^38^ ^]^ For instance, Tate et al. reported an antibody‐based PROTAC that demonstrated tumor cell targeting.^[^ ^39^ ^]^ However, the transmembrane efficacy of such antibody‐PROTACs is impeded by the intrinsic limitations of antibody‐drug conjugates (ADCs) in cellular transport. Furthermore, ADCs carry the risk of eliciting immune responses, thereby causing in vivo immunogenic hazards. Aptamers are emerging as superior alternatives owing to their high affinity and specificity, low immunogenic potential, and reduced molecular size. By leveraging macropinocytosis, this drugtamer‐PROTAC conjugation approach facilitates swift penetration through tumor cell membranes, substantially improving the potency and selectivity of protein degradation, and attenuating potential immunogenic risks. Wei et al. recently reported folate‐coupled PROTAC prodrugs for targeted delivery of PROTACs.^[^ ^40^ ^]^However, the advantages of these prodrugs have not been evaluated in animal models. The tumor‐targeting efficiency, in vivo potency, and toxicity of the designed drugtamer‐PROTAC prodrugs were validated in MDA‐MB‐231 xenograft mouse models.

To augment the therapeutic efficacy of PROTAC, the co‐administration of small‐molecule anticancer inhibitors has been widely evaluated in clinical trials.^[^ ^5^, ^6^, ^9^, ^10^ ^]^ Such combined therapeutic strategies have demonstrated notable synergistic benefits in clinical settings. Despite the advantages, traditional “cocktail” treatments encounter difficulties in ensuring balanced drug delivery to tumor sites, primarily due to the unique pharmacokinetic profiles of each drug and the complexity of controlling drug ratios. The limitations of PROTAC‐based drug combinations underscore the importance of developing innovative methods for precise co‐delivery of PROTAC and synergistic drugs. In this context, this study determined the optimal dosing ratio of **FU** and **NP** (2:1) and embedded two Fdu molecules into the AS1411 aptamer to achieve precise co‐delivery at the desired ratio. The best synergistic antitumor effects were observed.

Traditional aptamer‐PROTAC conjugates usually encounter limitations in their drug‐loading capacity, particularly when multiple drug molecules are sequentially conjugated to aptamers.^[^ ^41^, ^42^ ^]^ Our design effectively circumvents these constraints by leveraging the structural resemblance between Fdu and thymidine nucleosides. Fdu was directly incorporated into the nucleic acid structure during solid‐phase synthesis of the aptamer. Subsequently, it was conjugated to NAMPT PROTAC to facilitate sequential loading of multiple drugs.

The drugtamer‐PROTAC conjugation approach exhibited remarkable effects in mitigating the toxicity associated with PROTACs. By modulating the in vivo distribution of PROTAC, **FU** and **NP** were preferentially concentrated in tumor tissues, thereby diminishing their toxicity to non‐target tissues. After in vivo analysis of **AS‐2F‐NP**, a pronounced decrease in **NP** levels was observed in non‐target organs, such as the liver, kidneys, and lungs, leading to attenuated toxic side effects in these tissues. Compared to drug combinations, conjugate **AS‐2F‐NP** ensured precise control over drug ratios and their uniform distribution in mouse models, effectively maximizing antitumor efficacy and minimizing the toxicity commonly associated with drug combinations. Therefore, **AS‐2F‐NP** were safer and more effective than the PROTAC‐based drug combination.

Despite these promising profiles, this study has several limitations. First, only breast cancer models were used in this study; therefore, expanding these findings to a broader range of cancer types is essential for a comprehensive assessment of the drugtamer‐PROTAC conjugation strategy. Second, further research is required to understand the long‐term effects and potential immune responses induced by drugtamer‐PROTAC conjugates. Given the “precise targeting and potent killing” attributes of the conjugates, optimizing the linker may be the key to enhancing the tumoricidal effects and broadening the therapeutic window. New linkers with better tumor‐responsive abilities and high drug release efficacies would further enhance the precision and safety of conjugates.

## 4 Conclusion

In summary, we developed a novel drugtamer‐PROTAC conjugation strategy to enhance the tumor‐targeting ability and antitumor potency of conventional PROTACs. Inspired by the synergism between NAMPT PROTAC and 5‐fluorouracil, a multifunctional smart prodrug conjugate. For aptamer‐drug conjugates, it remains challenging to sequentially incorporate various drugs into aptamers. In this study, based on the optimal drug combination ratio, two molecules of **Fdu** replaced natural thymine nucleotides to ensure synthetic inconvenience and precise drug release. Conjugate **AS‐2F‐NP** showed improved aqueous solubility and cellular uptake, resulting in the degradation of NAMPT in nucleolin‐overexpressing MDA‐MB‐231 cells. In animal models, **AS‐2F‐NP** is activated by GSH in the TME and achieves precise co‐delivery of synergistic **Fdu** and **NP**, leading to enhanced antitumor potency (TGI >92%) and reduced side effects. Thus, the drugtamer‐PROTAC conjugation strategy provides an effective approach for the precise co‐delivery of PROTAC and its synergistic drugs, which may have broad applications in PROTAC‐based drug development.

## 5 Experimental Section

### Experimental Subheading Synthesis and Structural Characterization of Intermediates and Target Compounds

In general, NMR spectra (^1^H and ^13^C) were obtained using Bruker AVANCE300 and AVANCE600 spectrometers with TMS as the internal standard and DMSO‐*d*_6_ as the solvent, with chemical shifts in ppm (*δ*). The mass spectra were recorded on an Esquire 3000 LC‐MS. TLC was performed on GF254 silica gel plates from Qingdao Haiyang Chemical, China, and silica gel column chromatography utilized their Silica gel 60 G. All materials, unless specified, were sourced commercial and used as received without additional purification. The details of the synthesis and characterization of intermediates and target compounds employed in this manuscript were described in the Supplementary Information.

### Chou‐Talalay Assays

Dose‐response curves were obtained for **FU** and **NP** as single agents, as well as at a constant ratio of their IC_50_ values, to determine the extent of synergy between these drugs. The Combination Index (CI) scores were calculated using CompuSyn software (ComboSyn, Inc), employing the Chou‐Talalay combination index method based on the principles of the median‐effect equation. Synergy between the two drugs was defined as CI <1, additivity as CI ≈1, and antagonism as CI > 1. Additionally, isobolograms were generated using CompuSyn to visualize the synergy. In essence, the quantities of each individual drug needed to achieve effects at nine different efficacy levels were calculated, and these were used as intercepts to generate an isobole connecting those points. Dose pairs for the combination therapy were then plotted, with points below the isobole considered synergistic, on the isobole indicating additivity, and above the isobole suggesting antagonism.^[^ ^29^ ^]^

### Confocal Microscopy

Cellular fluorescence images were acquired on a Leica TCS SP5 confocal microscope equipped with an Argon‐Helium‐Neon laser (Leica Microsystems Inc., Exton, PA). MDA‐MB‐231 cells (10×10^4^) were seeded in glass‐bottom confocal dishes and incubated overnight at 37 °C. Subsequently, MDA‐MB‐231 cells were treated with 500 nM of **AS‐2F‐NP‐Cy3**, **AS‐Cy3**, and **CRO‐Cy3**, respectively, in serum‐free cell culture medium. After incubating for 1 h at 37 °C, the cells were washed three times with PBS solution. Following that, the cells were treated with 4% formaldehyde for 15 min at room temperature and washed with PBS solution three times. After a 15 min treatment with DAPI, the cells were rinsed with PBS three times and visualized using a confocal microscope.

For confocal imaging of endocytosis pathways, MDA‐MB‐231 cells (2×10^4^) were seeded in glass‐bottom confocal dishes and incubated overnight at 37°C. MDA‐MB‐231 cells in glass‐bottom confocal dishes should be pre‐incubated with the macropinocytosis inhibitor (EIPA), clathrin pathway inhibitor (Chlorpromazine), or caveolae pathway inhibitor (Filipin) at various concentrations for 30 min before the addition of **AS‐2F‐NP**. Other experimental steps were the same as described above.

### Colony Formation Assay

MDA‐MB‐231 cells were meticulously harvested, resuspended in growth medium, and precisely seeded into six‐well plates using a single‐cell suspension technique. After seeding, an exact cell count adjusted the density to 1000 cells per well, followed by the addition of DMEM medium to each well, reaching a precise volume of 1000 µL. Following a closely monitored 7‐day incubation, **AS‐2F‐NP**, **AS**, **NP**, **FU**, and **2FU+NP** were individually added to the respective plates. Another 7‐day period of cultured cell growth ensued, culminating in the removal of the culture medium. Adherent cells underwent a dual PBS rinse and meticulous fixation with 4% paraformaldehyde. Each well received 200 µL of a 1% crystal violet dye solution from the esteemed Beyotime Institute of Biotechnology, ensuring complete coverage. After a precisely timed 15‐minute ambient incubation, the plate underwent a thorough tap water wash and subsequent air‐drying for assay detection preparation.

### Study of Specificity and Binding Ability

The specificity and binding ability of **AS‐2F‐NP‐Fam**, **AS‐Fam**, or **CRO‐Fam** (final DNA concentrations: 250 nM) were explored by flow cytometry. MDA‐MB‐231 cells (1×10^5^) were incubated in the corresponding binding buffer at 4 °C for 40 min, followed by washing with washing buffer for 3 times. Then, precipitated cells were suspended in 400 µL binding buffer at 4 °C for flow cytometric analysis on a flow cytometer (BD Accuri C6). Data were analyzed using FlowJo software. **CRO‐Fam** was used as negative control, and **AS‐Fam** was used as a positive control.

### Binding Buffer

4.5 g L^−1^ glucose, 5 mM MgCl_2_, 0.1 mg mL^−1^ yeast tRNA (Sigma Aldrich), and 1 mg mL^−1^ BSA (Fisher Scientific) in Dulbecco's PBS (Sigma). Washing buffer: 4.5 g L^−1^ glucose and 5 mM MgCl_2_ in Dulbecco's PBS (Sigma).

### The Release of AS‐2F‐NP Conjugate In Vitro

**AS‐2F‐NP** at a concentration of 100 µM in water was first transferred into centrifuge tube, and then incubated with 5 mM DTT in 5 mL PBS (containing 0.5% Tween 80). The control group was conducted by immersing the **AS‐2F‐NP** in the PBS solution without DTT. The treatment groups were incubated at 37 °C under continuous shaking at a rate of 100 rpm min^−1^. Compounds concentrations at different time intervals were determined by HPLC using RP‐C18 column (5 µm, 4.6 × 150 mm) with 0.8 mL mi^−1^n. Mobile phase: A:100 mM TEAA pH = 7.0, B: CAN; Column temperature:40 °C; detection, UV, 260 nm.

### Proteome Assay

MDA‐MB‐231 cells in good condition were digested and 9 × 10^7^ cells were seeded in nine cell‐culture dishes for 24 h. Then, **AS‐2F‐NP**, **2FU+NP,** and PBS in the medium were incubated with cells, respectively. Repeat 3 groups for each compound. After incubation, cells were washed with PBS and then lysed by RIPA lysis buffer on the ice for 30 min. Total cell protein was obtained from the supernatant collected by centrifuging the cell lysate (12 000 g, 4 °C). Subsequently, the cell lysate (100‐200 µg) was sent to OEBiotech company for proteomic assay. Briefly, cellular samples were processed to extract total proteins, a portion of which was utilized for protein concentration determination and SDS‐PAGE analysis. Another portion was subjected to trypsin digestion and labeling, followed by equal mixing of the labeled samples for chromatographic separation. Subsequently, the samples were subjected to LC‐MS/MS analysis, and the acquired data were subjected to comprehensive data analysis.

### Bioinformatical Analysis

Following protein identification and quantification using Proteome Discovery v1.4 software, the expression profiles of proteins were obtained in each sample. The first and foremost step involved checking data quality through Pearson correlation analysis. In this study, each experimental group was replicated three times, and data were processed by filtering out outliers using Mean Absolute Differences (MAD) and imputing missing values through a random forest‐based algorithm. After consolidating the data, linear models (limma v3.52.4 R package) were employed to analyze significantly differentially expressed proteins between the two groups (The above experimental procedures were conducted by OEBiotech company). Volcano plots, expression pattern clustering heatmaps, Venn analysis, and other methods were employed for differential comparison group data. To gain a deeper understanding of differentially expressed proteins in the target KEGG pathway, this stud was focused on the MAPK signaling pathway and integrated it with proteins of interest. Lastly, enrichment analysis was conducted for the proteins of interest using R packages cluster Profiler v4.4.4 and org.Hs.eg.db v3.15.0.

### RNA Extraction and Real‐Time Quantitative PCR (qPCR)

The p65 and ErK gene expression effect of conjugates on MDA‐MB‐231 cells at different concentrations (0.5 µM and 1 µM) was assessed by the qPCR assay. Briefly, cells in good condition were digested and seeded with 3 × 10^5^ cells in each well of the 6‐well plates for 24 h. Then, 500 µL **AS‐2F‐NP** in the medium were incubated with cells for 24 h. After incubation, cells were washed with 1 × PBS, and total RNA was S44 extracted using RNAiso PLUS (TaKaRa Bio Inc, Shiga, Japan) according to the manufacturer's protocol. Complementary DNAs (cDNAs) were synthesized from 1 µg of purified total RNAs using Prime Script RT Master Mix (TaKaRa Bio Inc). Real‐time qPCR was executed on qTOWER3 Real‐time PCR Thermal Cycler (Analytik Jena, Jena, Germany) in reaction mixtures containing TB Green Premix Ex Taq (TaKaRa Bio Inc), cDNA, and forward and reverse primers, and cycling conditions of: 95 °C for 3 mins; followed by 40 cycles of 94 °C for 10 secs, 60 °C for 20 secs, and 72 °C for 20 secs; and final extension at 72 °C for 10 mins. The primer sets used were based on the following genes: ErK forward: 5′‐TCAACACCACCTGCGACCT‐3′, ErK reverse: 5′‐CGTAGCCACCTGCGACCT‐3′; GAPDH forward: 5′‐AACATCATCCCTGCTTCCAC‐3′, GAPDH reverse: 5′‐GACCACCTGGTCCTCAGTGT‐3′; p65 forward: 5′‐AAGATCTGCCGAGTGAACCG‐3′, p65 reverse:5′‐GCCTGGTCCCGTGAAATACA‐3′. Target gene expressions were normalized to the internal loading control gene GAPDH using 2‐ΔΔCT method. The mean CT value of target genes in the experimental group was normalized to the CT value of GAPDH to give a ΔCT value. The ΔCT value was then further normalized to control samples to obtain ΔΔCT value.

### In Vitro Antiproliferative Assay

Cells were seeded in 96‐well transparent plates at a density of ≈5 × 10^3^ cells well^−1^ and incubated in a humidified atmosphere with 5% CO_2_ at 37 °C for 24 h. Solutions of 10 µM **AS‐2F‐NP**, **CRO‐2F‐NP**, and **AS** were prepared in PBS (**NP** and **FU** were dissolved in DMSO with a concentration of 10 mm and diluted to achieve a concentration of 10 µM with PBS). Serial three‐fold dilutions of the solutions were performed with serum‐containing medium to create a concentration range from 13.7 to 10 000 nm. The cell culture medium was removed, and the solutions were added to triplicate wells with different concentrations. The medium containing 0.1% DMSO served as the control. After incubating for 72 h, 10 µL of cell counting kit‐8 (CCK8) solution was added to each well, and the plate was further incubated for 0.5‐1 h. The absorbance (OD) was measured using a Biotek Synergy H2 (Lab systems) at 405 nm. The concentration causing 50% inhibition of cell growth (IC_50_) was determined using the Logit method. Each experiment was conducted three times.

### Western Blotting

MDA‐MB‐231 cell lines were seeded at a density of 4.0 × 10^5^ cells well^−1^ in 6‐well transparent plates (Corning). The test compounds were added 24 h after seeding, and the cells were incubated for an additional 24 h. Subsequently, the cells were washed twice with cold PBS and lysed with 60 µL of ice‐cold lysis buffer containing 1% protease and phosphatase inhibitors (Roche). After 30 min on ice, the cells were scraped off and centrifuged at 12 000 rpm for 15 min at 4 °C to obtain the protein lysate. The protein extract was denatured in a 100 °C water bath and analyzed on 10% SDS‐PAGE gels. The gels were transferred onto a PVDF membrane (Merck Millipore), and blocking was performed with 5% BSA Buffer (5% Bovine Serum Albumin in TBST) for 2 h at room temperature. Subsequently, the membranes were probed with infrared secondary antibodies. After three washes with TBST, the blots were scanned using a LI‐COR Odyssey imaging system. Protein levels were quantified based on the gray values of the bands in the resulting images, with the control group serving as the standard.

Image J software was used to statistically analyze the grayscale values of the target protein blots and internal reference protein blots. According to the formula SF1, the remaining percentage of the target protein under different concentrations of target compound was calculated separately.

(1) $$R_{p}=\frac{A_{t}/A_{i}}{B_{t}/B_{i}}\times 100\%\;\mathrm{SF}1$$

*R_p_* represents the remaining percentage of the target protein; *A_t_* represents the grayscale value of the target protein blot in the treated group; *A_i_* represents the grayscale value of the internal reference protein blot in the treated group; *B_t_* represents the grayscale value of the target protein blot in the blank group; *B_i_* represents the grayscale value of the internal reference protein blot in the blank group.

The curve was plotted between the concentration of the compound and the remaining percentage of the target protein by using GraphPad Prism 8 software, and the DC_50_ of the target compound was calculated accordingly.

The remaining percentage of the target protein corresponding to the optimal degradation activity was selected. According to the formula SF2, the *D*_max_ was calculated

(2) $$D_{\max}=1-R_{p}\;\mathrm{SF}2$$

### In Vivo Imaging

All the animal protocols were assessed and approved by the Committee on Ethics of Medicine, Navy Medical University (SMMU82030105). BALB/C nude female mice (certificate SCXK‐2021‐0013, weighing 18−20 g) were obtained from Changzhou Cavens Experimental Animal Co., Ltd. 100 µL of **AS‐2F‐NP**‐**Cy3**, **AS**‐**Cy3** and **CRO**‐**Cy3** at a single dose of 5 µm were given to two groups of the MDA‐MB‐231 xenografts bearing BALB/C nude mice, respectively, by intravenous route (iv) via the tail vein. The nude mice were anesthetized and in vivo fluorescent imaging was carried out 4 and 8 h postinjection using Lumina XR imaging system. All the mice were sacrificed right after the last imaging. Tumors and major organs (hearts, lungs, livers, spleens, and kidneys) were collected and imaged with the in vivo imaging system (Lumina XR).

### In Vivo Therapy

The efficacy experiment in vivo was evaluated using the MDA‐MB‐231 tumor xenograft in mice. MDA‐MB‐231 cells (6 × 10^6^ cells/animal) were subcutaneously into the flank area of the female nude mice (5−6 weeks old). When tumors reached an average volume of 150 mm^3^ after implantation. nude mice were randomly divided into six groups. Five groups of nude mice were given intravenously with **AS** (10 µm kg^−1^) and **AS‐2F‐NP** (10 µm kg^−1^) in PBS and **FU** (1.3 mg kg^−1^), **NP** (11.8 mg kg^−1^)**, 2FU+NP** (2.6 mg kg^−1^+11.8 mg kg^−1^) in solution with formula (0.5% DMSO+5% ethanol + 0.5% Tween 80 + ddH_2_O) every other day with a volume of 100 µL per injection. The doses of **FU** and **NP** were equivalent to 10 µm kg^−1^. The control group was given an equal volume of PBS. During the treatment, tumor size was measured using vernier caliper, and monitored body weight every four days. After 19 days treatment, the mice were killed. The major organs (hearts, lungs, livers, spleens, and kidneys) were dissected, collected and weighed. The tumors were taken out of the mice and pictured. The tumor volume was calculated by this formula, volume = AB^2^/2. A and B are the length and width dimension of the tumor, respectively. Data were analyzed by 1‐way ANOVA test. *P* level <0.05 was considered statistically significant.

### Histology

After 30 days post‐treatment, the heart, liver, spleen, lung, kidney, and tumors of all treatment groups were dissected and fixed with 4% paraformaldehyde. The tissue of organs was sliced and stained by Bios Biological Company.

### Statistical Analysis

All the data were presented as mean ± sd. Student's *t*‐test or 1‐way analyses of variance (ANOVA) were performed in the evaluation of statistical. *P* level <0.05 was considered statistically significant.

## Conflict of Interest

The authors declare no conflict of interest.

## Supporting information

Supporting Information

## Data Availability

The data that support the findings of this study are available from the corresponding author upon reasonable request.
