# Supplementary material for: Drugtamer‐PROTAC Conjugation Strategy for Targeted PROTAC Delivery and Synergistic Antitumor Therapy
Source: Adv Sci (Weinh). 2024 Apr 19;11(25):2401623. doi: 10.1002/advs.202401623 (PMC11220662; doi:10.1002/advs.202401623)
Supplement: Supplementary file 1 — Supporting Information [file ADVS-11-2401623-s001.pdf]

ADVANCED SCIENCE
Open Access

Supporting Information

for *Adv. Sci.*, DOI 10.1002/advs.202401623

Drugtamer-PROTAC Conjugation Strategy for Targeted PROTAC Delivery and Synergistic Antitumor Therapy

Shipeng He, Yuxin Fang, Yaojin Zhu, Ziyang Ma, Guoqiang Dong* and Chunquan Sheng**

Supporting Information

Drugtamer-PROTAC Conjugation Strategy for Targeted PROTAC Delivery and Synergistic Antitumor Therapy

Shipeng He[†*b], *Yuxin Fang*[†a], *Yaojin Zhu*[b], *Ziyang Ma*[a], *Guoqiang Dong*[*a] *and Chunquan Sheng*[*a]

[a] The Center for Basic Research and Innovation of Medicine and Pharmacy (MOE), School of Pharmacy, Second Military Medical University (Naval Medical University), 325 Guohe Road, Shanghai 200433, P.R. China

[b] Institute of Translational Medicine, Shanghai University, 99 Shangda Road, Shanghai 200444, P.R. China.

E-mail: shengcq@smmu.edu.cn, gdong@smmu.edu.cn, heshipeng@shu.edu.cn.

Table of Contents

Figure S1. The mechanism of action of **FU** and its derivatives.................................S4

Table S1. CI values of **FU-NP** combined administration...S4

Figure S2. The dose-effect of **FU-NP** was calculated by CompuSyn software S5

Figure S3. Stability of Cy3-modified conjugates in 10% FBS..................................S5

Figure S4. Degradation activity of the target compounds in MDA-MB-231 cells......S6

Figure S5. Degradation Effects of target compounds in MCF-10A cells S6

Figure S6. The antiproliferative activity of target compounds against MDA-MB-231 and MCF-10A cells, respectively...S7

Figure S7. The ability of **AS-2F-NP** to inhibit the growth of MDA-MB-231 cells compared with **FU**, **AS**, **NP**, and the combination of **2FU**: **NP** at the same dose concentration gradient..S7

Figure S8. Proteome level analysis of MDA-MB-231 cells treated with **AS-2F-NP**. S8

Figure S9. Nicotinate and nicotinamide metabolism signaling pathways of MDA-MB-231 cell line treated by **AS-2F-NP**. S8

Figure S10. Drug metabolism signaling pathways of MDA-MB-231 cell line treated by **AS-2F-NP**. S9

Figure S12. Microphotographs of tissue sections from different organs with H&E staining in different treatment groups S10

Figure S13. Immunohistochemistry of tumor tissue sections with NAMPT staining from the six treatment groups *in vivo* assay. S10

Figure S14. Uncropped blot corresponding to Figure 4A..S11

Figure S15. Uncropped blot corresponding to Figure 4B..S11

Figure S16. Uncropped blot corresponding to Figure S5. ....S12

Materials and Methods...S12

The spectra of the synthesized compounds...S34

Reference S56

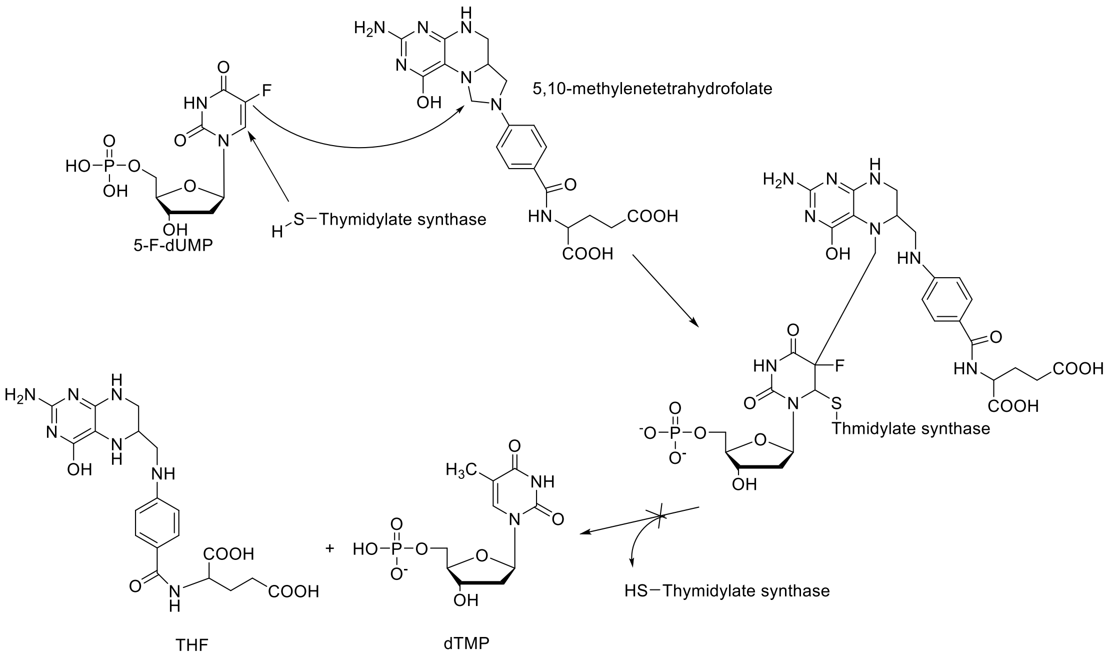

Figure S1. The mechanism of action of **FU** and its derivatives. Initially, **FU** and its derivatives are converted into **Fdu** *in vivo*, which inhibit the enzyme thymidylate synthase. This inhibition prevents the methylation of deoxyuridine monophosphate (dUMP) into deoxythymidine monophosphate (dTMP), thereby disrupting DNA synthesis. Additionally, **FU** can be converted into 5-fluorouridine, which incorporates into RNA as a pseudouridine and interferes with protein synthesis.

Table S1. CI values of **FU-NP** combined administration

**FU:NP=1:2**			**FU:NP=1:1**			**FU:NP=2:1**		
Dose	Fa	CI	Dose	Fa	CI	Dose	Fa	CI
3.00	0.89	1.63	2.00	0.85	1.27	1.50	0.85	0.65
1.50	0.84	1.37	1.00	0.79	1.04	0.75	0.81	**0.46**
0.75	0.78	1.10	0.50	0.69	0.99	0.38	0.78	**0.29**
0.38	0.71	0.86	0.25	0.62	0.72	0.19	0.69	**0.26**
0.19	0.6	0.78	0.13	0.52	0.60	0.09	0.60	**0.21**
0.09	0.49	0.67	0.06	0.47	**0.38**	0.05	0.55	**0.14**
0.05	0.37	0.61	0.03	0.35	**0.35**	0.02	0.47	**0.11**
0.02	0.28	**0.50**	0.02	0.27	**0.28**	0.01	0.43	**0.07**
0.01	0.20	**0.43**	0.01	0.26	**0.15**	0.01	0.31	**0.06**

Fa: fraction affected, CI: combination index.

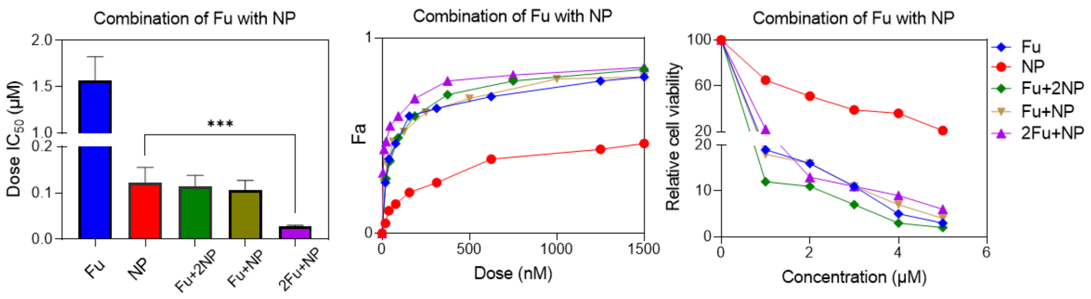

Figure S2. The dose-effect of **FU-NP** was calculated by CompuSyn software

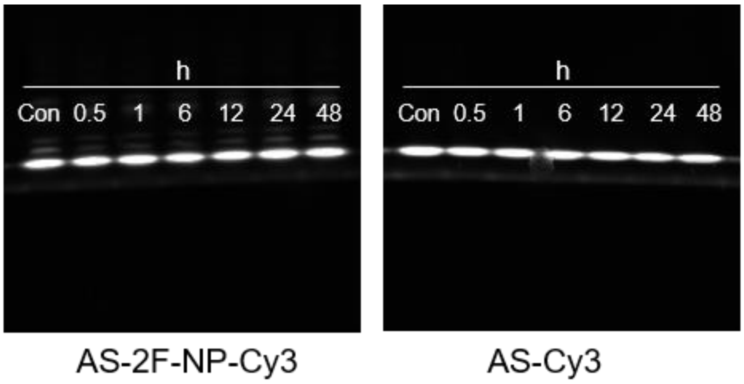

Figure S3. Stability of Cy3-modified conjugates in 10% FBS.

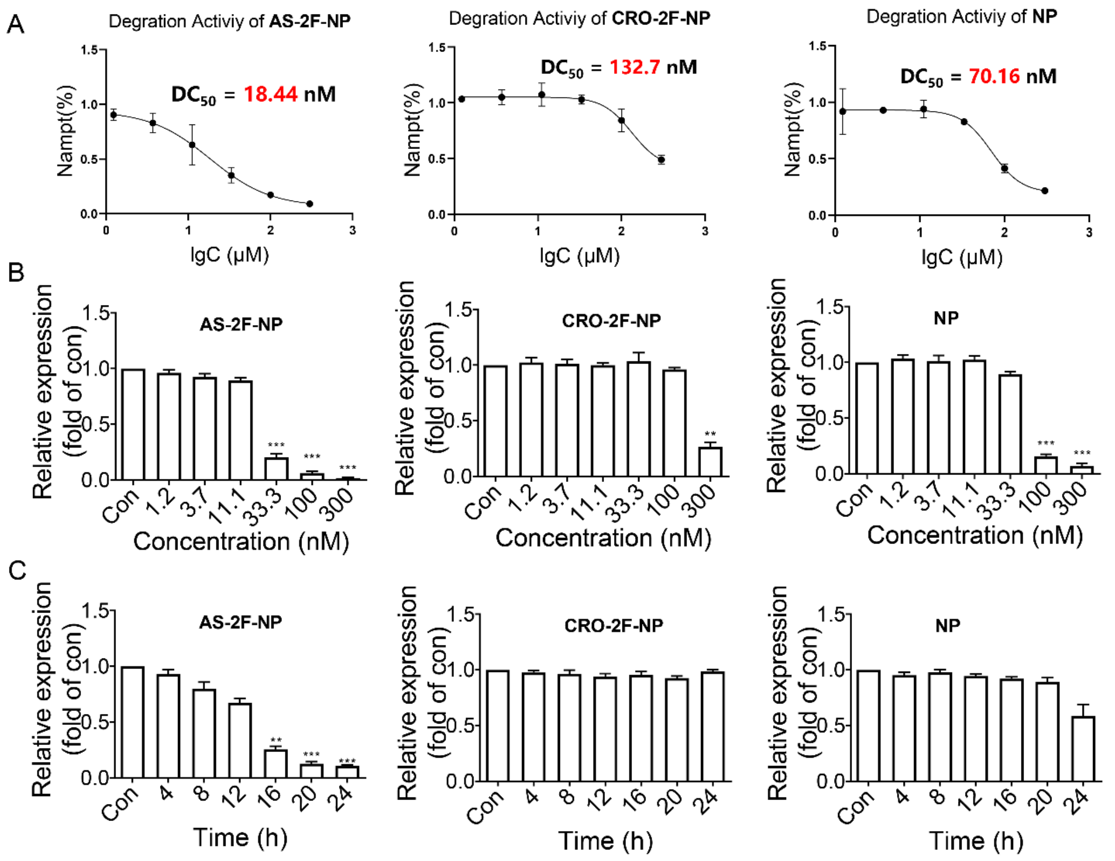

Figure S4. Degradation activity of the target compounds. (A) The DC_{50} values of target compounds in MDA-MB-231 cells. (B) The grayscale statistics of NAMPT degradation by target compounds in MDA-MB-231 cells in a concentration-dependent manner. (C) The grayscale statistics of NAMPT degradation by target compounds in MDA-MB-231 cells in time-dependent manner.

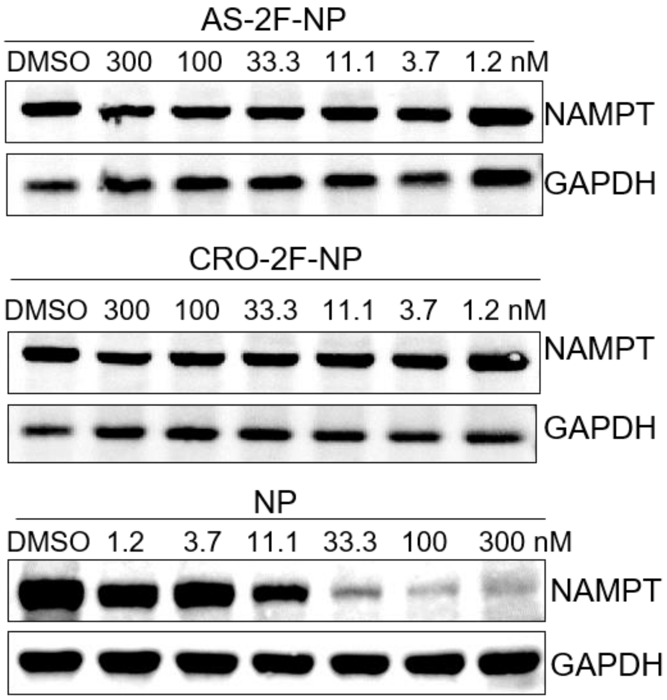

Figure S5. Degradation Effects of target compounds in MCF-10A cells

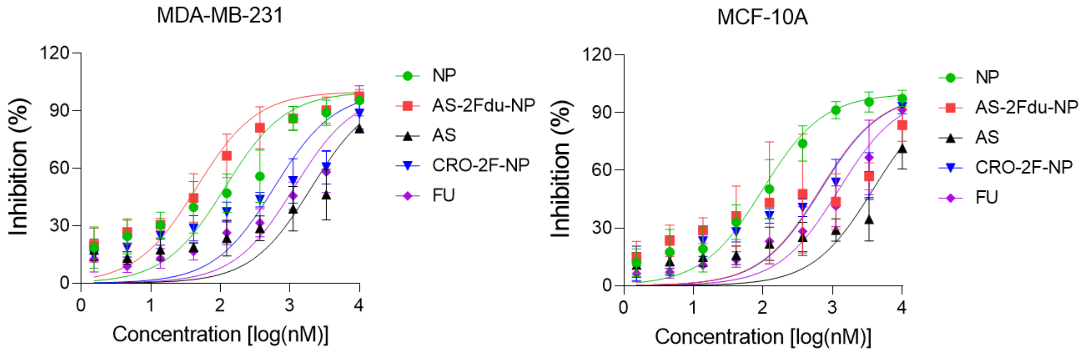

Figure S6. The antiproliferative activity of target compounds against MDA-MB-231 and MCF-10A cells, respectively.

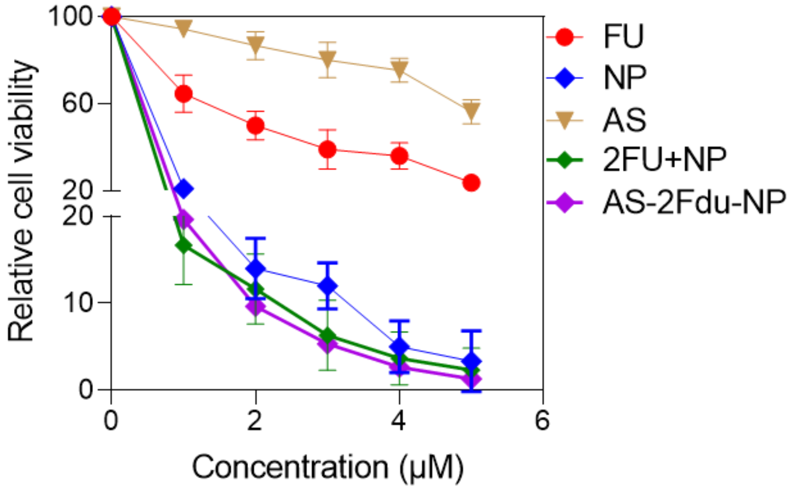

Figure S7. The ability of **AS-2F-NP** to inhibit the growth of MDA-MB-231 cells compared with **FU**, **AS**, **NP**, and the combination of **2FU**: **NP** at the same dose concentration gradient.

A

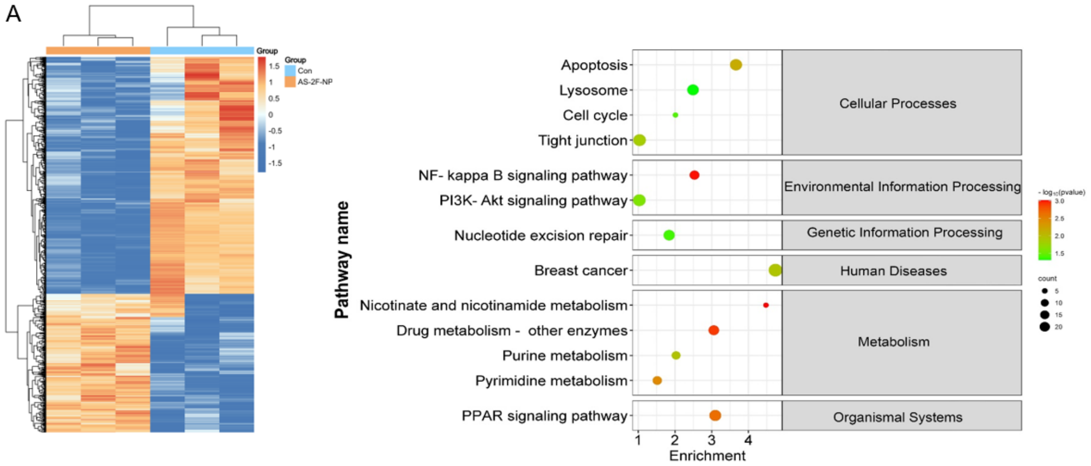

Figure S8. Proteome level analysis of MDA-MB-231 cells treated with **AS-2F-NP**. (A) Heatmap analysis of 1006 differentially expressed proteins in the cell lysate of MDA-MB-231 cells treated with **AS-2F-NP** compared to the PBS-treated control (n = 3; $p < 0.05$). (B) The bubble map of KEGG enrichment analysis revealed different signaling pathways influenced by **AS-2F-NP** in MDA-MB-231 cells.

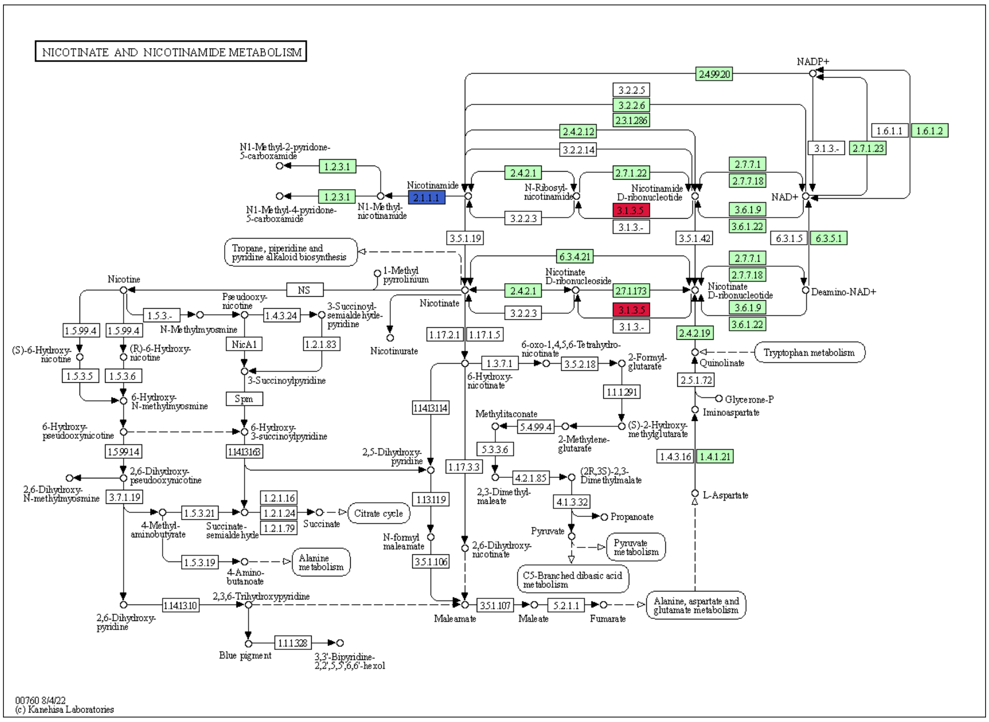

Figure S9. Nicotinate and nicotinamide metabolism signaling pathways of MDA-MB-231 cell line treated by **AS-2F-NP**. Red means up, blue means down.

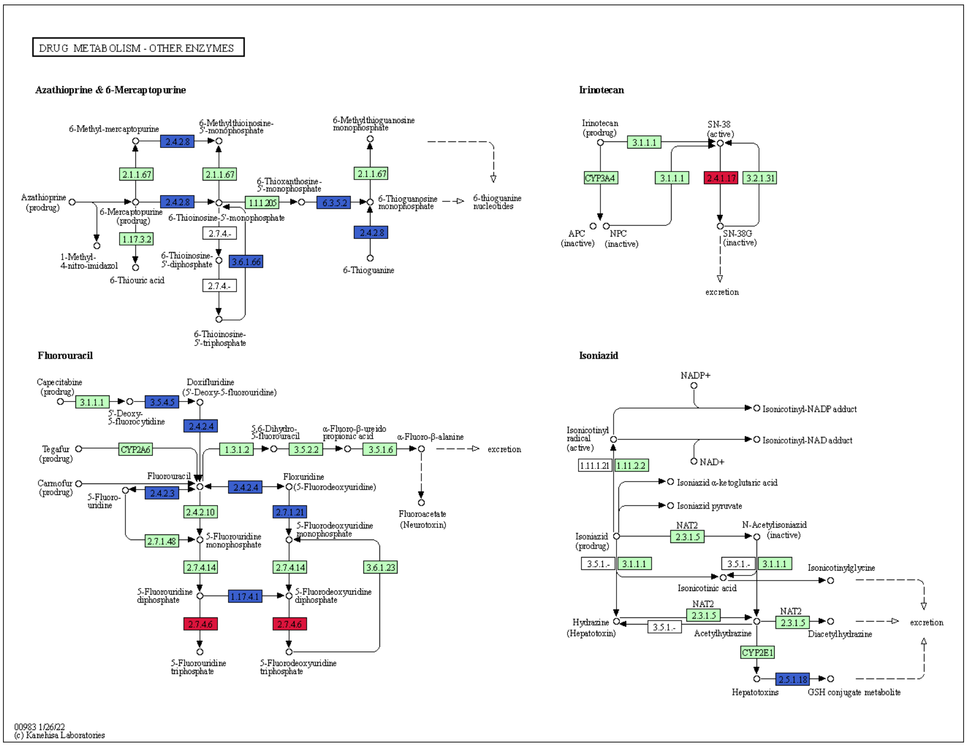

Figure S10. Drug metabolism signaling pathways of MDA-MB-231 cell line treated by **AS-2F-NP**. Red means up, blue means down.

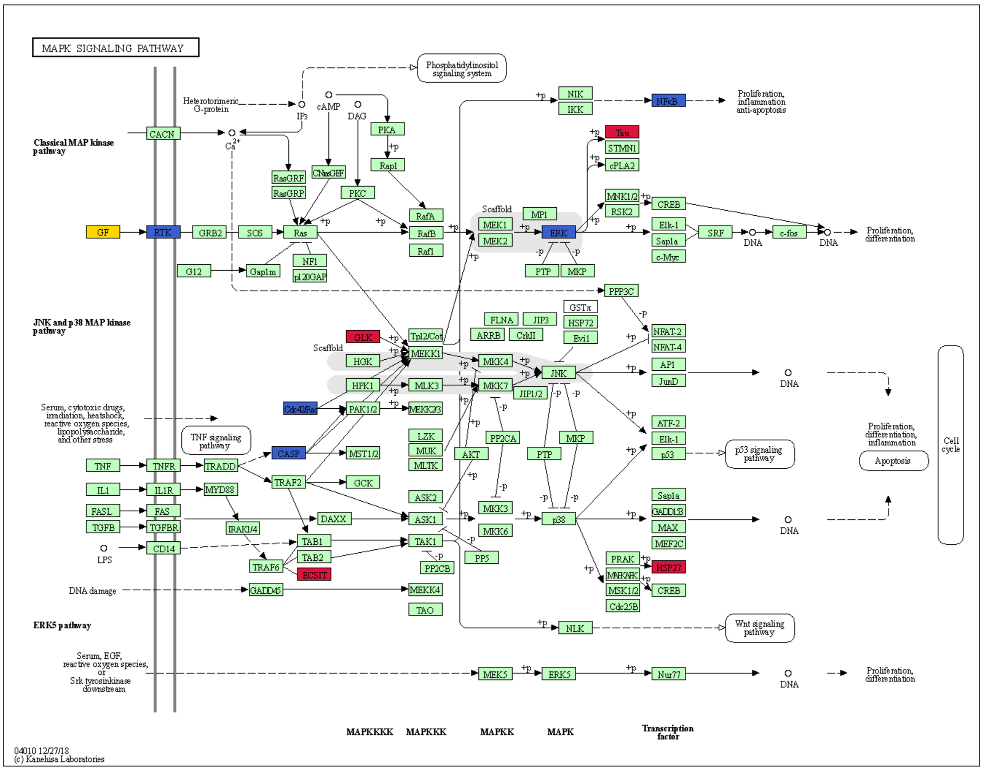

Figure S11. MAPK signaling pathways of MDA-MB-231 cell line treated by **AS-2F-NP**. Red means up, blue means down.

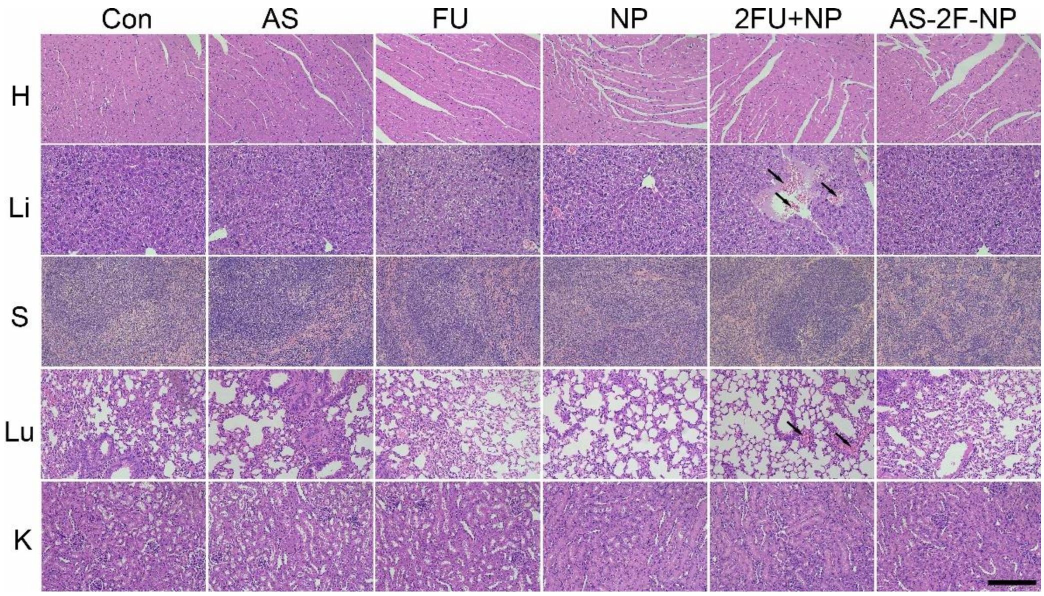

Figure S12. Microphotographs of tissue sections from different organs with H&E staining in different treatment groups. Scale bars, 200 μm.

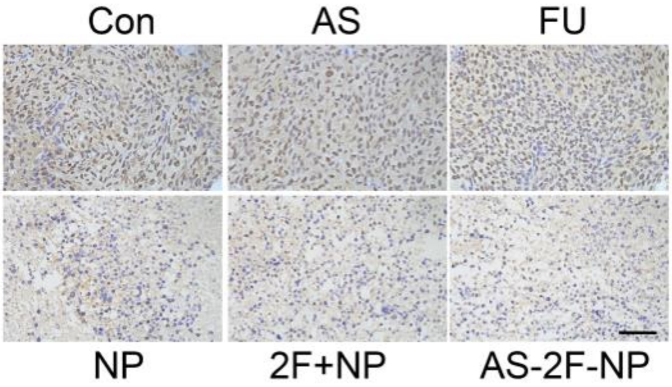

Figure S13. Immunohistochemistry of tumor tissue sections with NAMPT staining from the six treatment groups *in vivo* assay. Error bar, 200 μm.

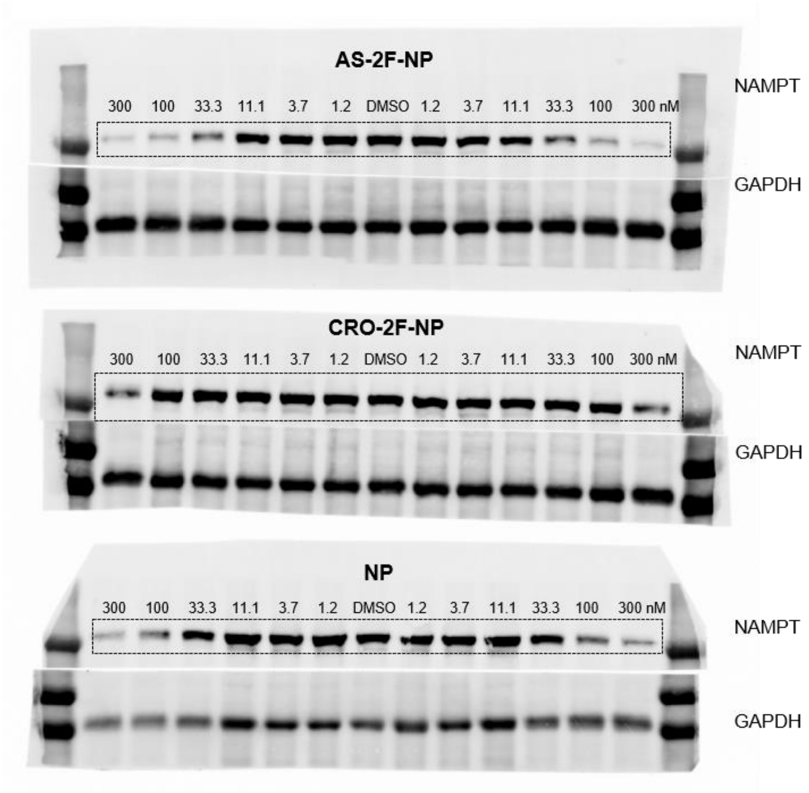

Figure S14. Uncropped blot corresponding to **Figure 4A**.

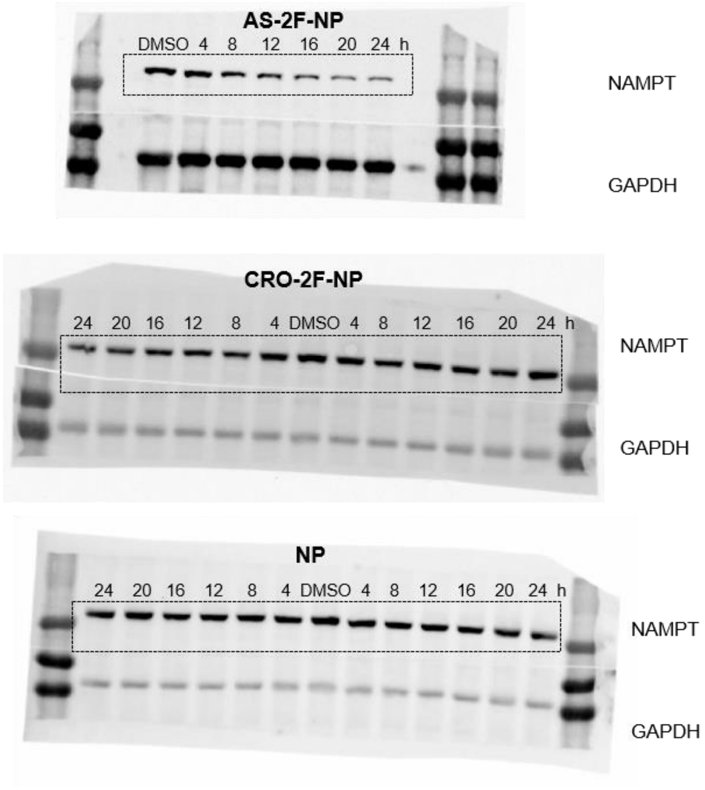

Figure S15. Uncropped blot corresponding to **Figure 4B**.

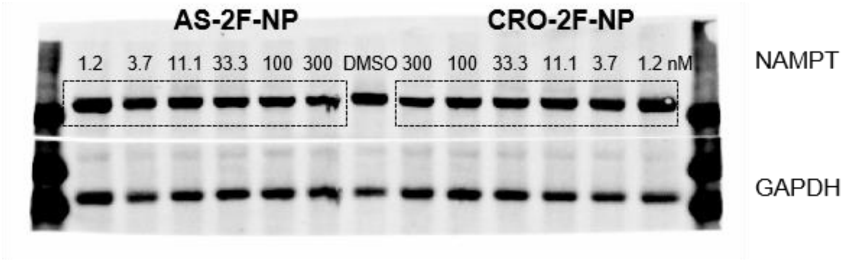

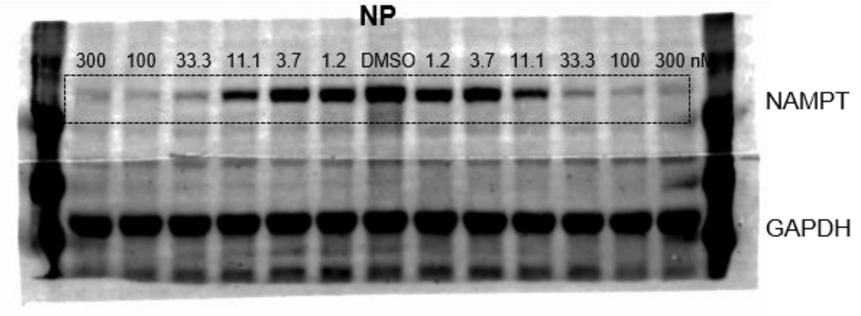

Figure S16. Uncropped blot corresponding to **Figure S5**.

Materials and Methods

Chemistry.

The synthesis of the key intermediate **1S** is outlined in **Scheme S1**. Initially, commercially available compound **12** and *tert*-butyl piperazine-1-carboxylate were reacted in the presence of Et_3N in DCM to yield compound **13** through a nucleophilic reaction. Subsequently, compound **13** underwent catalytic reduction with H_2 gas in presence of Pd/C, producing compound **14**. The key intermediate **15** was obtained through a condensation reaction between compound **14** and pyridin-3-ylmethanamine. Compound **15** underwent deprotection, followed by a coupling reaction with Methyl 4-(bromomethyl)benzoate, resulting in the formation of intermediate **16**. Finally, compound **1S** was produced through a hydrolysis reaction of intermediate **16**.

Scheme S1[1]

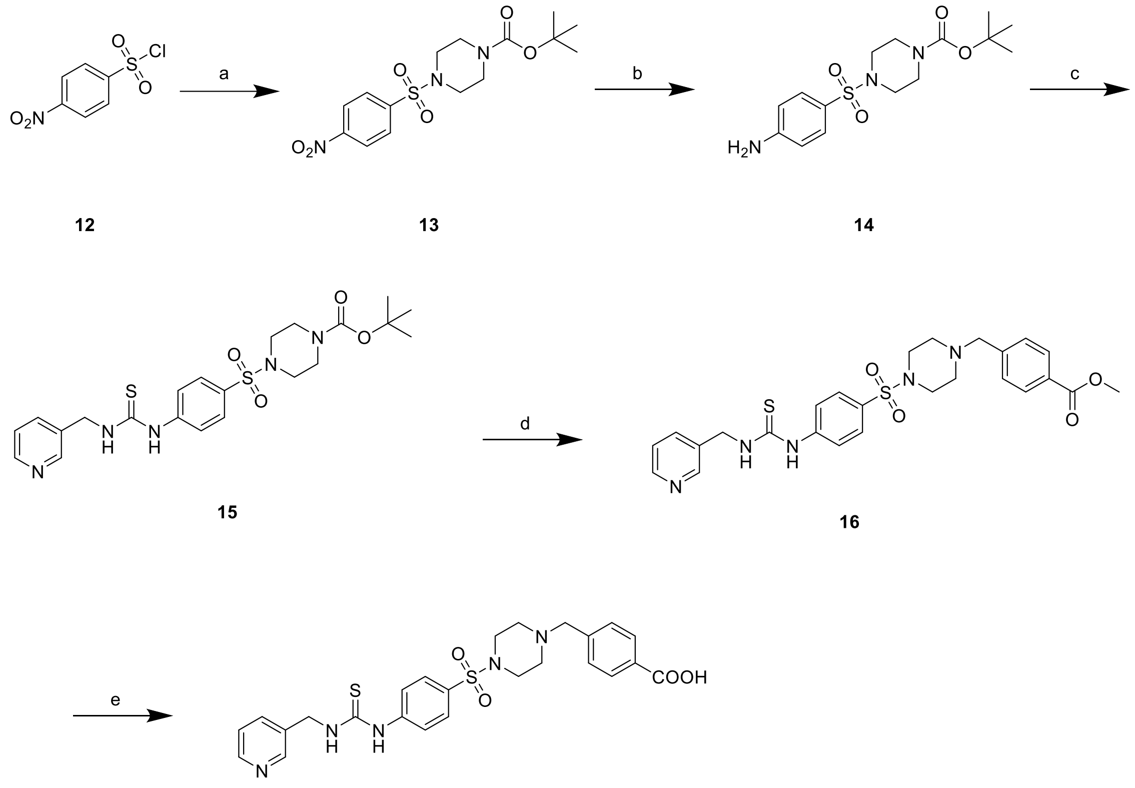

1S

Reagents and conditions: (a) *tert*-butyl piperazine-1-carboxylate, Et_3N, DCM, rt, 2 h, 92%; (b) Pd/C, H_2, DCM, rt, overnight, 96%; (c) 1,1-thiocarbonyldiimidazole, DCM, rt, overnight, pyridin-3-ylmethanamine, DCM, rt, overnight, 39%; (d) TFA, DCM, rt, 4 h, methyl 4-(bromomethyl)benzoate, Et_3N, DCM, rt, 4 h, 52%; (e) LiOH, THF/MeOH/H_2O, rt, 3 h, 53%.

The synthesis of DPCs is outlined in **Scheme S2**. First, the hydroxyl group in VHL ligand **5** reacted with 4-nitrophenyl carbonochloridate in the presence of DMAP to afford intermediate **6**. Key intermediate **7** was synthesized by the reaction of compound **6** with 2,2'-disulfanediylbis(ethan-1-ol) under catalysis by DMAP. Then, the compound **7** was condensed with dimethyl-4-oxo-3,8,11,14-tetraoxa-5-azahexadecan-16-oic acid to afford intermediate **8** in the presence of HOBT and EDCI. Subsequently, the Boc

protecting group was removed by TFA to give compound **9**, which was further condensed with intermediate **1S** in the presence of HATU and DIPEA to afford compound **10**. The esterification of compound **10** with succinic anhydride gave intermediate **11**. Finally, compound **11** was conjugated with AS-2Fdu-NH_2 in the presence of Sulfo-NHS and EDCI to yield the AFPC **AS-2F-NP**. Next, the CRO-PROTAC conjugate (herein denoted compound **SJ-2F-NP**) and the Fam- or Cy3-modified AFPC conjugates (herein denoted compounds **AS-2F-NP-Fam**, **AS-2F-NP-Cy3**, **AS-2F-NP-Cy3**) were synthesized using a similar protocol as described for **AS-2F-NP** synthesis. The structures of key intermediates were confirmed by ^{1}H-NMR, ^{13}C-NMR spectroscopy and high-resolution mass spectrometry (HRMS). The aptamer-modified conjugates were purified by reversed phase high-performance liquid chromatography (RP-HPLC) and confirmed by mass spectrometry.

Scheme S2

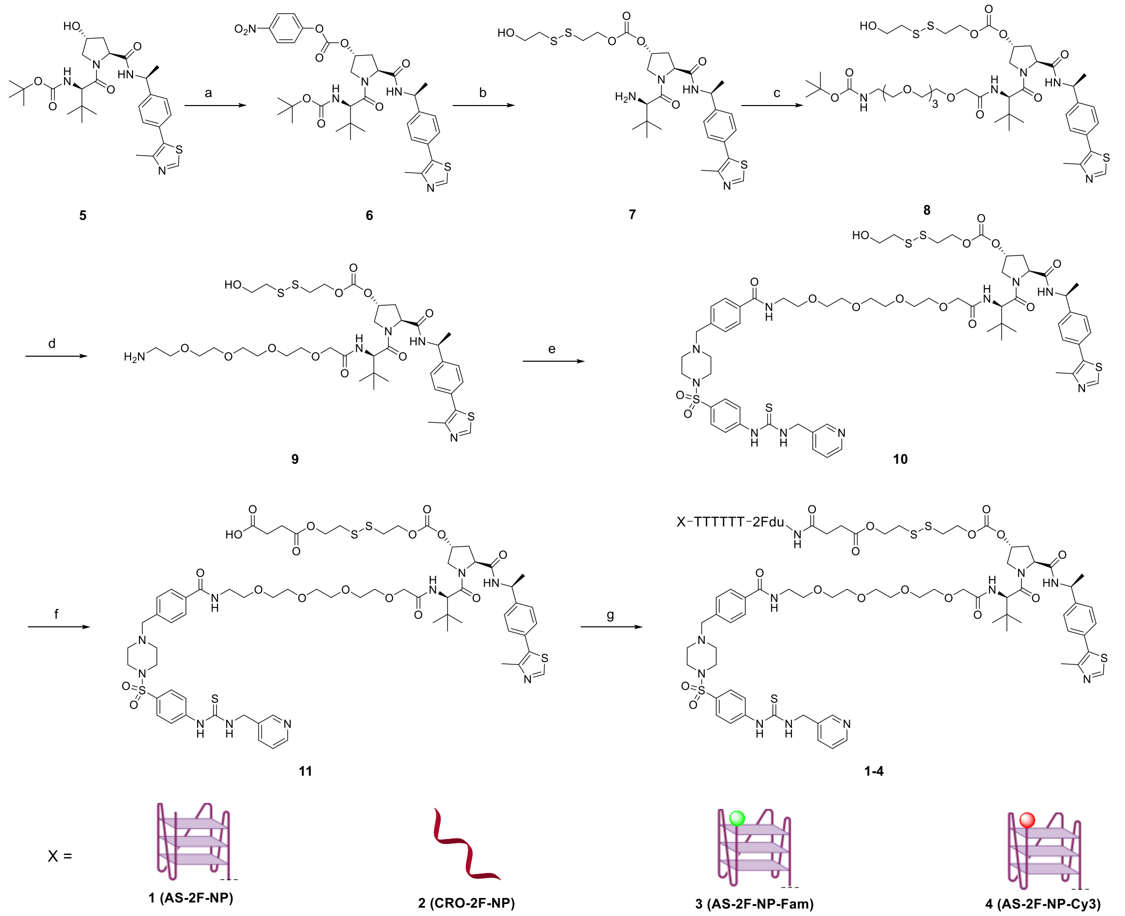

Reagents and conditions: (a) 4-nitrophenylchloroformate, DMAP, DCM, rt, 12 h, 47%; (b) 2,2'-disulfanediylbis(ethan-1-ol), DMAP, DCM, TFA, DCM, rt, 10 h, 44%; (c) 2,2-dimethyl-4-oxo-3,8,11,14,17-pentaoxa-5-azanonadecan-19-oic acid, HOBT, EDCI, DMF, rt, 5 h, 65%; (d) TFA, DCM, rt, 2 h, 79%; (e) compound **1S**, HOBT, EDCI, DMF, rt, 10 h, 56%; (f) succinic anhydride, DMAP, DCM, rt, 3 h, 72%; (g) aptamers, Sulfo-NHS, EDCI, dd-H_2O, DMF, 0.5 M Na_2CO_3/$NaHCO_3$, 12 h, 5–20%.

Synthesis and Structural Characterization of Intermediates and Target Compounds.

General. NMR spectra (^{1}H and ^{13}C) were obtained using Bruker AVANCE300 and AVANCE600 spectrometers with TMS as the internal standard and DMSO-d_6 as the solvent, with chemical shifts in ppm (δ). The mass spectra were recorded on an Esquire 3000 LC-MS. TLC was performed on GF254 silica gel plates from Qingdao Haiyang Chemical, China, and silica gel column chromatography utilized their Silica gel 60 G. All materials, unless specified, were sourced commercial and used as received without additional purification.

***tert*-Butyl 4-((4-nitrophenyl)sulfonyl)piperazine-1-carboxylate (13).** A stirred solution of compound **12** (4.47 g, 20.23 mmol) in DCM (50 mL) was prepared. *tert*-Butyl 1-piperazinecarboxylate (3.77 g, 20.23 mmol) and Et_3N (4.10 g, 40.46 mmol) was dissolved in DCM (50 mL). The mixture was added dropwise to the solution of compound **12** and stirred at room temperature for 2 h. The progress of the reaction was monitored by TLC. Then, the solvent was removed under reduced pressure, and the resulting residue was collected without purification to give compound **13** (6.95 g, 92%) as a white solid. ^{1}H NMR (600 MHz, DMSO-d_6) δ 8.43 (d, J = 8.65 Hz, 2H), 8.00 (d, J = 8.63 Hz, 2H), 3.40 (s, 4H), 2.95 (t, J = 4.77 Hz, 4H), 1.33 (s, 9H). ^{13}C NMR (150 MHz, DMSO-d_6) δ 153.8, 150.6, 141.2, 129.6, 125.2, 79.8, 46.1, 28.4.

***tert*-Butyl 4-((4-aminophenyl)sulfonyl)piperazine-1-carboxylate (14)**. Compound **13** (6.50 g, 17.51 mmol) was dissolved in DCM (50 mL) and 30% Pd/C (1.95 g, 18.32 mmol) was added. The reaction mixture was stirred at room temperature overnight

under the H_2 gas. When completed, the solution was filtered through diatomite, followed by evaporation under reduced pressure. The residue was purified by column chromatography (DCM: MeOH = 100: 0 – 100: 3) to yield compound **14** (5.76 g, 96%) as a white solid. ^{1}H NMR (600 MHz, DMSO-d_6) δ 7.33 (d, J = 8.64 Hz, 2H), 6.64 (d, J = 8.70 Hz, 2H), 6.11 (s, 2H), 3.37 (s, 4H), 2.73 (t, J = 4.69 Hz, 4H), 1.34 (s, 9H). ^{13}C NMR (150 MHz, DMSO-d_6) δ 153.8, 130.1, 119.4, 113.2, 79.7, 46.2, 28.4.

***tert*-Butyl 4-((4-(3-(pyridin-3-ylmethyl)thioureido)phenyl)sulfonyl)piperazine - 1-carboxylate (15).** Intermediate **14** (5.00 g, 14.66 mmol) and 1,1-thiocarbonyldiimidazole (3.92 g, 21.99 mmol) was dissolved in DCM (100 mL) into a round-bottom flask and stirred overnight. Pyridin-3-ylmethanamine (1.59 g, 14.66 mmol) was dissolved in DCM (50 mL) and was added dropwise into the above solution. The reaction was stirred at room temperature overnight and monitored by TLC. When completed, the solvent was removed under reduced pressure. The residue was purified by column chromatography (DCM: MeOH = 100: 4 – 100: 10) to give compound **15** (2.83 g, 39%) as a white solid. ^{1}H NMR (600 MHz, DMSO-d_6) δ 10.12 (s, 1H), 8.61 (s, 1H), 8.57 (d, J = 1.44 Hz, 1H), 8.47 (d, J = 4.63 Hz, 1H), 7.80 (d, J = 8.76 Hz, 2H), 7.76 (d, J = 8.09 Hz, 1H), 7.66 (d, J = 8.76 Hz, 2H), 7.37 (dd, J = 7.49, 4.86 Hz, 1H), 4.79 (d, J = 5.45 Hz, 2H), 3.39 (s, 4H), 2.83 (t, J = 4.86 Hz, 4H), 1.33 (s, 9H). ^{13}C NMR (150 MHz, DMSO-d_6) δ 181.2, 153.9, 149.4, 148.7, 144.6, 135.7, 134.6, 128.9, 123.9, 121.9, 79.80, 46.2, 45.2, 28.4.

Methyl 4-((4-((4-(3-(pyridin-3-ylmethyl)thioureido)phenyl)sulfonyl)piperazin - 1-yl)methyl)benzoate (16). Intermediate **15** (2.50 g, 5.09 mmol) was dissolved in

DCM (100 mL) into a round-bottom flask. Then, trifluoroacetic acid (25 mL) was added dropwise, and the reaction mixture was stirred at room temperature for 4 h. After completion of the reaction monitored by TLC, the DCM solvent and trifluoroacetic acid were removed under reduced pressure. The resulting residue was dissolved in DMF (20 mL), and then methyl 4-(bromomethyl)benzoate (1.17 g, 5.09 mmol) and Et_3N (1.32 g, 10.18 mmol) were added. The mixture was stirred at room temperature for 4 h. Upon completion of the reaction, the solvent DMF was removed under reduced pressure. The residue was purified by column chromatography (DCM: MeOH = 100: 0 – 100: 5) to give compound **16** (1.43 g, 52%) as a white solid. ^{1}H NMR (600 MHz, DMSO-d_6) δ 10.16 (s, 1H), 8.66 (s, 1H), 8.59 (d, J = 1.57 Hz, 1H), 8.49 (dd, J = 4.74, 1.50 Hz, 1H), 7.88 (d, J = 8.34 Hz, 2H), 7.82 (d, J = 8.72 Hz, 2H), 7.78 (d, J = 7.83 Hz, 1H), 7.66 (d, J = 8.85 Hz, 2H), 7.39 (d, J = 8.12 Hz, 3H), 4.81 (s, 2H), 3.83 (s, 3H), 3.54 (s, 2H), 2.89 (s, 4H), 2.44 (s, 4H). HRMS (ESI) *m/z* Calculated for $C_{26}H_{29}N_5O_4S_2^+$ $[M+H]^+$ 540.1734, found 540.1739.

4-((4-((4-(3-(Pyridin-3-ylmethyl)thioureido)phenyl)sulfonyl)piperazin-1-yl)methyl)benzoic acid (1S). Intermediate **16** (1.30 g, 2.41 mmol) was dissolved in a mixed solvent composed of THF/MeOH/H_2O (v: v: v = 3: 2: 1, 21 mL). LiOH (0.20 g, 8.35 mmol) was added to the solution and stirred for 3h. Upon completion of the reaction monitored by TLC, the mixed solvent was removed under reduced pressure. The residue was purified by column chromatography (DCM: MeOH = 100: 0 – 100: 10) to give compound **1S** (0.67 g, 53%) as a white solid. ^{1}H NMR (600 MHz, DMSO-d_6) δ 11.95 (s, 1H), 11.70 (s, 1H), 9.73 (s, 1H), 8.80 (s, 1H), 8.76 (d, J = 4.84 Hz, 1H),

8.42 (d, *J* = 7.77 Hz, 1H), 8.05 (d, *J* = 8.65 Hz, 2H), 7.95 (t, *J* = 6.01 Hz, 1H), 7.91 (d, *J* = 8.06 Hz, 2H), 7.78 (d, *J* = 7.96 Hz, 2H), 7.64 (d, *J* = 8.71 Hz, 2H), 4.93 (d, *J* = 5.32 Hz, 2H), 4.47 (s, 2H), 3.67-3.72 (m, 4H), 3.26 (s, 4H). HRMS (ESI) *m/z* Calculated for $C_{25}H_{27}N_5O_4S_2^+$ $[M+H]^+$ 526.1577, found 526.1585.

The key intermediate **1S** was prepared according to the literature.[1]

***tert*-Butyl ((*R*)-3,3-dimethyl-1-((2*S*,4*R*)-2-(((*S*)-1-(4-(4-methylthiazol-5-yl) phenyl)ethyl)carbamoyl)-4-(((4-nitrophenoxy)carbonyl)oxy)pyrrolidin-1-yl)-1-oxobutan-2-yl)carbamate (6)**. A solution of compound **5** (2.20 g, 4.04 mmol) in DCM (50 mL) was stirred. Separately, 4-nitrophenylchloroformate (4.07 g, 20.20 mmol) and DMAP (2.47 g, 20.20 mmol) was dissolved in DCM (100mL). This mixture was added dropwise to the solution of compound **5** at room temperature, and the reaction mixture was stirred at room temperature for 12 h continuously. The reaction was monitored by TLC. The DCM solvent was removed under reduced pressure, and the residue was purified by column chromatography (PE: EA = 9: 1 – 1: 2) to give compound **6** (1.35 g, 47%) as a white solid. ^1H NMR (600 MHz, DMSO-*d*$_6$) δ 8.98 (s, 1H), 8.45 (d, *J* = 7.45 Hz, 1H), 8.25 (d, *J* = 8.37 Hz, 2H), 7.65 (d, *J* = 8.45 Hz, 2H), 7.37-7.44 (m, 4H), 6.65 (d, *J* = 7.72 Hz, 1H), 5.26-5.30 (m, 1H), 4.89-4.94 (m, 1H), 4.50 (t, *J* = 8.37 Hz, 1H), 4.05-4.09 (m, 1H), 4.01-4.04 (m, 1H), 3.76 (d, *J* = 9.54 Hz, 1H), 2.45 (s, 3H), 2.33-2.36 (m, 1H), 2.00-2.04 (m, 1H), 1.38 (d, *J* = 6.96 Hz, 3H), 1.32 (s, 9H), 0.95 (s, 9H).

(3*R*,5*S*)-1-((*R*)-2-amino-3,3-dimethylbutanoyl)-5-(((*S*)-1-(4-(4-methylthiazol-

5-yl)phenyl)ethyl)carbamoyl)pyrrolidin-3-yl (2-((2-hydroxyethyl)disulfaneyl)ethyl) carbonate (7). To a stirred solution of 2,2'-Disulfanediylbis(ethan-1-ol) (1.26 g, 8.17 mmol) and DMAP (0.4 g, 3.27 mmol) in DCM (100 mL), the solution of intermediate **6** (1.16 g, 1.63 mmol) in DCM (100mL) was added dropwise at room temperature. The reaction mixture was stirred at room temperature for 10 h. Trifluoroacetic acid (10 mL) was then added dropwise, and the reaction was stirred at room temperature for another 2 h. After the reaction was complete, the DCM solvent and trifluoroacetic acid were removed under reduced pressure. The residue was purified by column chromatography (DCM: MeOH = 100: 7 – 100: 12) to give compound **7** (0.45 g, 44%) as a white solid. ^{1}H NMR (600 MHz, DMSO-d_6) δ 8.99 (s, 1H), 8.59 (d, J = 7.80 Hz, 1H), 8.00 (s, 2H), 7.37-7.45 (m, 4H), 5.22 (t, J = 3.75 Hz, 1H), 4.90-4.94 (m, 2H), 4.51-4.54 (m, 1H), 4.35-4.38 (m, 1H), 4.32-4.35 (m, 1H), 4.06 (d, J = 11.54 Hz, 1H), 4.02 (d, J = 4.87 Hz, 1H), 3.69 (dd, J = 8.75, 3.68 Hz, 1H), 3.63 (t, J = 6.36 Hz, 2H), 3.00 (t, J = 6.24 Hz, 2H), 2.82 (t, J = 6.43 Hz, 2H), 2.45 (s, 3H), 2.37-2.40 (m, 1H), 1.99-2.03 (m, 1H), 1.38 (d, J = 6.97 Hz, 3H), 1.03 (s, 9H). HRMS (ESI) *m/z* Calculated for $C_{28}H_{40}N_4O_6S_3^+$ [M+H]$^+$ 625.2183, found 625.2153.

***tert*-Butyl ((*R*)-16-((2*S*,4*R*)-4-(((2-((2-hydroxyethyl)disulfaneyl)ethoxy) carbonyl)oxy)-2-(((*S*)-1-(4-(4-methylthiazol-5-yl)phenyl)ethyl)carbamoyl)pyrrolidine-1-carbonyl)-17,17-dimethyl-14-oxo-3,6,9,12-tetraoxa-15-azaoctadecyl)carbamate (8).** 2,2-Dimethyl-4-oxo-3,8,11,14,17-pentaoxa-5-azanonadecan-19-oic acid (253mg, 0.72 mmol) and HOBT (149mg, 1.08

mmol) and EDCI (208mg, 1.08mmol) was dissolved in DMF (10 mL) and stirred at room temperature for 10 min. Then, a solution of intermediate **7** (450 mg, 0.72 mmol) in DMF (5 mL) was added and the reaction was stirred at room temperature for another 5 h. After completion of the reaction, saturated aqueous NaCl (400 mL) was added, and the mixture was extracted with ethyl acetate (50 mL × 3). The organic phases were combined and dried with anhydrous sodium sulfate. After filtration, ethyl acetate was removed under reduced pressure and the residue was purified by silica gel column chromatography (DCM: MeOH = 100: 4 – 100: 8) to give compound **8** (448 mg, 65%) as a white solid. ^{1}H NMR (600 MHz, DMSO-d_6) δ 8.98 (s, 1H), 8.46 (d, J = 7.63 Hz, 1H), 7.43-7.44 (m, 2H), 7.36-7.40 (m, 3H), 6.73 (s, 1H), 5.16 (s, 1H), 4.88-4.92 (m, 2H), 4.44-4.46 (m, 1H), 4.32-4.34 (m, 1H), 4.30-4.32 (m, 1H), 3.99 (d, J = 12.02 Hz, 1H), 3.85 (s, 2H), 3.78 (dd, J = 8.24, 3.84 Hz, 1H), 3.56-3.63 (m, 8H), 3.52-3.55 (m, 4H), 3.49-3.52 (m, 5H), 3.37 (t, J = 6.21 Hz, 2H), 2.98-3.00 (m ,2H), 2.80-2.82 (m, 2H), 2.45 (s, 3H), 2.32-2.35 (m, 1H), 1.98-2.03 (m, 1H), 1.38 (d, J = 7.13 Hz, 3H), 1.36 (s, 9H), 0.95 (s, 9H). HRMS (ESI) *m/z* Calculated for $C_{43}H_{67}N_5O_{13}S_3^+$ $[M+H]^+$ 958.3970, found 958.3945.

(3*R*,5*S*)-1-((*R*)-17-Amino-2-(*tert*-butyl)-4-oxo-6,9,12,15-tetraoxa-3-azaheptadecanoyl)-5-(((*S*)-1-(4-(4-methylthiazol-5-yl)phenyl)ethyl)carbamoyl)pyrrolidin-3-yl (2-((2-hydroxyethyl)disulfaneyl)ethyl) carbonate (9). To a stirred solution of intermediate **8** (420 mg, 0.44 mmol) in DCM (16 mL), trifluoroacetic acid (4 mL) was added dropwise and then the reaction mixture was stirred at room temperature for 2 h. After the reaction was complete, the solvent

and trifluoroacetic acid were removed under reduced pressure. The residue was purified by silica gel column chromatography (DCM: MeOH = 100: 8–100: 15) to give compound **9** (298 mg, 79%) as a white solid. ^{1}H NMR (600 MHz, DMSO-d_6) δ 8.97 (s, 1H), 8.50 (d, J = 7.58 Hz, 1H), 7.43-7.46 (m, 3H), 7.37 (d, J = 8.28 Hz, 2H), 5.16 (s, 1H), 4.90 (t, J = 7.19 Hz, 1H), 4.45 (t, J = 9.09 Hz, 2H), 4.33-4.34 (m, 1H), 4.31-4.32 (m, 1H), 3.99-4.01 (m, 1H), 3.98 (s, 2H), 3.79 (dd, J = 8.29, 3.62 Hz, 1H), 3.60-3.64 (m, 7H), 3.56-3.59 (m, 10H), 2.97-3.00 (m ,4H), 2.81 (t, J = 6.44 Hz, 2H), 2.45 (s, 3H), 2.34-2.37 (m, 1H), 1.98-2.03 (m, 1H), 1.38 (d, J = 6.97 Hz, 3H), 0.96 (s, 9H), 0.93 (s, 2H).

(3*R*,5*S*)-1-((*R*)-18-(*tert*-Butyl)-1,16-dioxo-1-(4-((4-((4-(3-(pyridin-3-ylmethyl)thioureido)phenyl)sulfonyl)piperazin-1-yl)methyl)phenyl)-5,8,11,14-tetraoxa-2,17-diazanonadecan-19-oyl)-5-(((*S*)-1-(4-(4-methylthiazol-5-yl)phenyl)ethyl)carbamoyl)pyrrolidin-3-yl(2-((2-hydroxyethyl)disulfaneyl)ethyl) carbonate (10). 4-((4-((4-(3-(Pyridin-3-ylmethyl)thioureido)phenyl)sulfonyl)piperazin-1-yl)methyl)benzoic acid (147 mg, 0.28 mmol), HOBT (57 mg, 0.42 mmol) and EDCI (81 mg, 0.42 mmol) was dissolved in DMF (10mL) and stirred at room temperature for 10 min. The solution of intermediate **9** (200 mg, 0.23 mmol) in DMF (5 mL) was added and the reaction was stirred at room temperature for another 5 h. After the reaction was complete, saturated aqueous NaCl (400 mL) was added, and then the mixture was extracted with ethyl acetate (50 mL × 3). The combined organic phases were dried with anhydrous sodium sulfate, filtered, and then evaporated under reduced pressure. The resulting residue was

purified by silica gel column chromatography (DCM: MeOH = 100: 10 – 100: 15) to give compound **10** (176 mg, 56%) as a white solid. ^{1}H NMR (600 MHz, DMSO-*d*$_6$) *δ* 10.11 (s, 1H), 8.98 (s, 1H), 8.62 (s, 1H), 8.57 (d, *J* = 1.73 Hz, 1H), 8.47 (dd, *J* = 3.16, 1.58 Hz, 1H), 8.45 (d, *J* = 7.76 Hz, 1H), 8.42 (t, *J* = 5.50 Hz, 1H), 7.79 (d, *J* = 8.74 Hz, 2H), 7.75-7.77 (m, 3H), 7.64 (d, *J* = 8.82 Hz, 2H), 7.43 (d, *J* = 8.21 Hz, 2H), 7.36-7.39 (m, 4H), 7.30 (d, *J* = 8.21 Hz, 2H), 5.16 (s, 1H), 4.90 (t, *J* = 7.29 Hz, 1H), 4.88 (t, *J* = 5.35 Hz, 1H), 4.79 (d, *J* = 5.35 Hz, 2H), 4.44 (t, *J* = 8.99 Hz, 2H), 4.31-4.34 (m, 2H), 3.99 (d, *J* = 12.15 Hz, 1H), 3.95 (s, 2H), 3.78 (dd, *J* = 8.28, 3.93 Hz, 1H), 3.60-3.63 (m, 2H), 3.58-3.60 (m, 2H), 3.50-3.56 (m, 17H), 3.38-3.41 (m ,2H), 2.99 (t, *J* = 6.33 Hz, 2H), 2.88 (s, 1H), 2.80 (t, *J* = 6.36 Hz, 2H), 2.45 (s, 3H), 2.43 (s, 4H), 2.32-2.36 (m, 1H), 1.98-2.03 (m, 1H), 1.37 (d, *J* = 7.01 Hz, 3H), 0.95 (s, 9H). HRMS (ESI) *m/z* Calculated for $C_{63}H_{84}N_{10}O_{14}S_5^+$ [M+H]$^+$ 1365.4845, found 1365.4830.

4-(2-((2-(((((3*R*,5*S*)-1-((*R*)-18-(*tert*-Butyl)-1,16-dioxo-1-(4-((4-((4-(3-(pyridin-3-ylmethyl)thioureido)phenyl)sulfonyl)piperazin-1-yl)methyl)phenyl)-5,8,11,14-tetraoxa-2,17-diazanonadecan-19-oyl)-5-(((*S*)-1-(4-(4-methylthiazol-5-yl)phenyl)ethyl)carbamoyl)pyrrolidin-3-yl)oxy)carbonyl)oxy)ethyl)disulfaneyl)ethoxy)-4-oxobutanoic acid (11). Intermediate **10** (50 mg, 0.037 mmol), Succinic anhydride (19 mg, 0.18 mmol) and DMAP (7 mg, 0.055 mmol) were dissolved in DCM (100mL). The reaction was stirred at room temperature for 3 h. Upon completion of the reaction monitored by TLC, the solvent DCM was removed under reduced pressure. The residue was purified by silica gel column chromatography (DCM: MeOH = 100: 10 – 100: 15) to give compound **11**

(39 mg, 72%) as a white solid. ^{1}H NMR (600 MHz, DMSO-d_6) δ 11.99 (s, 1H), 10.25 (s, 1H), 8.97 (s, 1H), 8.76 (s, 1H), 8.57 (d, J = 1.89 Hz, 1H), 8.45-8.48 (m, 2H), 8.42 (t, J = 5.68 Hz, 1H), 7.82 (d, J = 8.67 Hz, 2H), 7.75-7.77 (m, 3H), 7.64 (d, J = 8.77 Hz, 2H), 7.43 (d, J = 8.17 Hz, 2H), 7.36-7.39 (m, 4H), 7.30 (d, J = 8.17 Hz, 2H), 5.16 (s, 1H), 4.89-4.93 (m, 1H), 4.79 (d, J = 5.43 Hz, 2H), 4.44-4.47 (m, 2H), 4.30-4.35 (m, 2H), 4.25 (t, J = 6.45 Hz, 2H), 4.01 (d, J = 12.11 Hz, 1H), 3.95 (s, 2H), 3.78 (dd, J = 8.40, 3.52 Hz, 1H), 3.58-3.60 (m, 2H), 3.50-3.56 (m, 17H), 3.39-3.42 (m, 2H), 3.00 (t, J = 6.25 Hz, 2H), 2.97 (t, J = 6.25 Hz, 2H), 2.88 (s, 1H), 2.50-2.52 (m, 2H), 2.47-2.48 (m, 2H), 2.45 (s, 3H), 2.43 (s, 4H), 2.33-2.36 (m, 1H), 1.99-2.04 (m, 1H), 1.37 (d, J = 6.92 Hz, 3H), 0.95 (s, 9H). HRMS (ESI) *m/z* Calculated for $C_{67}H_{88}N_{10}O_{17}S_5^+$ $[M+H]^+$ 1465.4963, found 1465.4994.

The synthesis of AS-2F-NP. To a solution of compound **11** (29.3 mg, 0.02 mmol), EDCI (5.0 mg, 0.025 mmol) in DMF (500 μL), Sulfo-NHS (5.5 mg, 0.025 mmol) dissolved in ddH_2O (250 μL) was slowly added and the reaction mixture was stirred for 2 h at room temperature. Activated compound **11** was then reacted with amino-modified nucleolin aptamer **AS-2Fdu-NH$_2$**. **AS-2Fdu-NH$_2$** (0.65 mg, 0.06 μmol) was dissolved in 0.5 M Na_2CO_3/$NaHCO_3$ buffer (100 μL, pH = 8.4), and 350 μL freshly prepared compound **11** *N*-hydroxysulfosuccinimide ester reaction solution was added. After stirred for 2.5 h, an additional 350 μL of the active ester reaction solution was added. The reaction mixture was stirred for 12 h at 37 °C. After reaction completed, the mixture was purified by RP-HPLC to give compound **AS-2F-NP**.[2, 3]

Target compounds **CRO-2F-NP**, **AS-2F-NP-Fam**, and **AS-2F-NP-Cy3** were

synthesized according to a similar procedure described for **AS-2F-NP**.

Chou-Talalay assays

We obtained dose-response curves for **FU** and **NP** as single agents, as well as at a constant ratio of their IC_{50} values, to determine the extent of synergy between these drugs. The Combination Index (CI) scores were calculated using CompuSyn software (ComboSyn, Inc), employing the Chou-Talalay combination index method based on the principles of the median-effect equation. Synergy between the two drugs was defined as CI < 1, additivity as CI around 1, and antagonism as CI > 1. Additionally, isobolograms were generated using CompuSyn to visualize the synergy. In essence, the quantities of each individual drug needed to achieve effects at nine different efficacy levels were calculated, and these were used as intercepts to generate an isobole connecting those points. Dose pairs for the combination therapy were then plotted, with points below the isobole considered synergistic, on the isobole indicating additivity, and above the isobole suggesting antagonism.[4]

Confocal microscopy

Cellular fluorescence images were acquired on a Leica TCS SP5 confocal microscope equipped with an Argon-Helium-Neon laser (Leica Microsystems Inc., Exton, PA). MDA-MB-231 cells (10×10^4) were seeded in glass-bottom confocal dishes and incubated overnight at 37°C. Subsequently, MDA-MB-231 cells were treated with 500 nM of **AS-2F-NP-Cy3**, **AS-Cy3**, and **CRO-Cy3**, respectively, in serum-free cell

culture medium. After incubating for 1h at 37°C, the cells were washed three times with PBS solution. Following that, the cells were treated with 4% formaldehyde for 15 min at room temperature and washed with PBS solution three times. After a 15 min treatment with DAPI, the cells were rinsed with PBS three times and visualized using a confocal microscope.

For confocal imaging of endocytosis pathways, MDA-MB-231 cells (2×10^4) were seeded in glass-bottom confocal dishes and incubated overnight at 37 °C. MDA-MB-231 cells in glass-bottom confocal dishes should be pre-incubated with the macropinocytosis inhibitor (EIPA), clathrin pathway inhibitor (Chlorpromazine), or caveolae pathway inhibitor (Filipin) at various concentrations for 30 min before the addition of **AS-2F-NP**. Other experimental steps were the same as described above.

Colony formation assay

MDA-MB-231 cells were meticulously harvested, resuspended in growth medium, and precisely seeded into six-well plates using a single-cell suspension technique. After seeding, an exact cell count adjusted the density to 1000 cells per well, followed by the addition of DMEM medium to each well, reaching a precise volume of 1000 μL. Following a closely monitored 7-day incubation, **AS-2F-NP**, **AS**, **NP**, **FU**, and **2FU+NP** were individually added to the respective plates. Another 7-day period of cultured cell growth ensued, culminating in the removal of the culture medium. Adherent cells underwent a dual PBS rinse and meticulous fixation with 4% paraformaldehyde. Each well received 200 μL of a 1% crystal violet dye solution from

the esteemed Beyotime Institute of Biotechnology, ensuring complete coverage. After a precisely timed 15-minute ambient incubation, the plate underwent a thorough tap water wash and subsequent air-drying for assay detection preparation.

Study of specificity and binding ability

The specificity and binding ability of **AS-2F-NP-Fam**, **AS-Fam**, or **CRO-Fam** (final DNA concentrations: 250 nM) were explored by flow cytometry. MDA-MB-231 cells (1×10^5) were incubated in the corresponding binding buffer at 4 ^oC for 40 min, followed by washing with washing buffer for 3 times. Then, precipitated cells were suspended in 400 μL binding buffer at 4 ^oC for flow cytometric analysis on a flow cytometer (BD Accuri C6). Data were analyzed using FlowJo software. **CRO-Fam** were used as negative control, and **AS-Fam** was used as a positive control.

Binding buffer: 4.5 g/L glucose, 5 mM $MgCl_2$, 0.1 mg/mL yeast tRNA (Sigma Aldrich), and 1 mg/mL BSA (Fisher Scientific) in Dulbecco's PBS (Sigma). Washing buffer: 4.5 g/L glucose and 5 mM $MgCl_2$ in Dulbecco's PBS (Sigma).

The release of AS-2F-NP conjugate *in vitro*

AS-2F-NP at a concentration of 100 μM in water was first transferred into centrifuge tube, and then incubated with 5 mM DTT in 5 mL PBS (containing 0.5% Tween 80). The control group was conducted by immersing the **AS-2F-NP** in the PBS solution without DTT. The treatment groups were incubated at 37 °C under continuous shaking at a rate of 100 rpm/min. Compounds concentrations at different time intervals

were determined by HPLC using RP-C18 column (5 μm, 4.6 × 150 mm) with 0.8 mL/min. Mobile phase: A:100 mM TEAA pH = 7.0, B: CAN; Column temperature:40 °C; detection, UV, 260 nm.

Proteome assay

MDA-MB-231 cells in good condition were digested and 9×10^7 cells were seeded in nine cell-culture dishes for 24 h. Then, **AS-2F-NP**, **2FU+NP** and PBS in the medium was incubated with cells, respectively. Repeat 3 groups for each compound. After incubation, cells were washed with PBS and then lysed by RIPA lysis buffer on the ice for 30 min. Total cell protein was obtained from the supernatant collected by centrifuging the cell lysate (12000 g, 4 °C). Subsequently, the cell lysate (100-200 μg) was sent to OEBiotech company for proteomic assay. Briefly, cellular samples were processed to extract total proteins, a portion of which was utilized for protein concentration determination and SDS-PAGE analysis. Another portion was subjected to trypsin digestion and labeling, followed by equal mixing of the labeled samples for chromatographic separation. Subsequently, the samples were subjected to LC-MS/MS analysis, and the acquired data were subjected to comprehensive data analysis.

Bioinformatical analysis

Following protein identification and quantification using Proteome Discovery v1.4 software, we obtained the expression profiles of proteins in each sample. The first and foremost step involved checking data quality through Pearson correlation analysis.

In our study, each experimental group was replicated three times, and data were processed by filtering out outliers using Mean Absolute Differences (MAD) and imputing missing values through a random forest-based algorithm. After consolidating the data, we employed linear models (limma v3.52.4 R package) to analyze significantly differentially expressed proteins between the two groups (The above experimental procedures were conducted by OEBiotech company). Volcano plots, expression pattern clustering heatmaps, Venn analysis, and other methods were employed for differential comparison group data. To gain a deeper understanding of differentially expressed proteins in the target KEGG pathway, we focused on the MAPK signaling pathway and integrated it with proteins of interest. Lastly, we conducted enrichment analysis for the proteins of interest using R packages cluster Profiler v4.4.4 and org.Hs.eg.db v3.15.0.

RNA extraction and real-time quantitative PCR (qPCR).

The p65 and ErK gene expression effect of conjugates on MDA-MB-231 cells at different concentrations (0.5 μM and 1 μM) was assessed by the qPCR assay. Briefly, cells in good condition were digested and seeded with 3 × 10^5 cells in each well of the 6-well plates for 24 h. Then, 500 μL **AS-2F-NP** in the medium were incubated with cells for 24 h. After incubation, cells were w ashed with 1 × PBS and total RNA was S44 extracted using RNAiso PLUS (TaKaRa Bio Inc, Shiga, Japan) according to the manufacturer's protocol. Com plementary DNAs (cDNAs) were synthesized from 1 μg of purified total RNAs

using Prime Script RT Master Mix (TaKaRa Bio Inc). Real-time qPCR was e xecuted on qTOWER3 Real-time PCR Thermal Cycler (Analytik Jena, Jena, Ge rmany) in reaction mixtures containing TB Green Premix Ex Taq (TaKaRa Bio Inc), cDNA, and forward and reverse primers, and cycling conditions of: 95 ° C for 3 mins; followed by 40 cycles of 94 °C for 10 secs, 60 °C for 20 secs, and 72 °C for 20 secs; and final extension at 72 °C for 10 mins. The primer sets used were based on the following genes: ErK forward: 5'-TCAACACCA CCTGCGACCT-3', ErK reverse: 5'-CGTAGCCACCTGCGACCT-3'; GAPDH for ward: 5'-AACATCATCCCTGCTTCCAC-3', GAPDH reverse: 5'-GACCACCTGG TCCTCAGTGT-3'; p65 forward: 5'-AAGATCTGCCGAGTGAACCG-3', p65 reve rse:5'-GCCTGGTCCCGTGAAATACA-3'. Target gene expressions were normaliz ed to the internal loading control gene GAPDH using 2-ΔΔCT method. The m ean CT value of target genes in the experimental group were normalized to th e CT value of GAPDH to give a ΔCT value. The ΔCT value was then further normalized to control samples to obtain ΔΔCT value.

***In vitro* antiproliferative assay.**

Cells were seeded in 96-well transparent plates at a density of approximately 5×10^3 cells/well and incubated in a humidified atmosphere with 5% CO_2 at 37 °C for 24 h. Solutions of 10 μM **AS-2F-NP**, **CRO-2F-NP**, and **AS** were prepared in PBS (**NP** and **FU** were dissolved in DMSO with a concentration of 10 mM and diluted to achieve a concentration of 10 μM with PBS). Serial three-fold dilutions of the solutions were

performed with serum-containing medium to create a concentration range from 13.7 to 10000 nM. The cell culture medium was removed, and the solutions were added to triplicate wells with different concentrations. The medium containing 0.1% DMSO served as the control. After incubating for 72 h, 10 μL of cell counting kit-8 (CCK8) solution was added to each well, and the plate was further incubated for 0.5-1 h. The absorbance (OD) was measured using a Biotek Synergy H2 (Lab systems) at 405 nm. The concentration causing 50% inhibition of cell growth (IC_{50}) was determined using the Logit method. Each experiment was conducted three times.

Western blotting.

MDA-MB-231 cell lines were seeded at a density of 4.0×10^5 cells/well in 6-well transparent plates (Corning). The test compounds were added 24 h after seeding, and the cells were incubated for an additional 24 h. Subsequently, the cells were washed twice with cold PBS and lysed with 60 μL of ice-cold lysis buffer containing 1% protease and phosphatase inhibitors (Roche). After 30 min on ice, the cells were scraped off and centrifuged at 12000 rpm for 15 min at 4 °C to obtain the protein lysate. The protein extract was denatured in a 100 °C water bath and analyzed on 10% SDS-PAGE gels. The gels were transferred onto a PVDF membrane (Merck Millipore), and blocking was performed with 5% BSA Buffer (5% Bovine Serum Albumin in TBST) for 2 h at room temperature. Subsequently, the membranes were probed with infrared secondary antibodies. After three washes with TBST, the blots were scanned using a LI-COR Odyssey imaging system. Protein levels were quantified based on the gray

values of the bands in the resulting images, with the control group serving as the standard.

Image J software was used to statistically analyze the grayscale values of the target protein blots and internal reference protein blots. According to the formula **SF1**, the remaining percentage of the target protein under different concentrations of target compound was calculated separately.

$$R_p = \frac{A_t/A_i}{B_t/B_i} \times 100\% \qquad \text{SF1}$$

R_p represents the remaining percentage of the target protein; A_t represents the grayscale value of the target protein blot in the treated group; A_i represents the grayscale value of the internal reference protein blot in the treated group; B_t represents the grayscale value of the target protein blot in the blank group; B_i represents the grayscale value of the internal reference protein blot in the blank group.

The curve was plotted between the concentration of the compound and the remaining percentage of the target protein by using GraphPad Prism 8 software, and the DC_{50} of the target compound was calculated accordingly.

The remaining percentage of the target protein corresponding to the optimal degradation activity was selected. According to the formula **SF2**, the D_{max} was calculated

$$D_{max} = 1\text{-}R_p \qquad \text{SF2}$$

In vivo imaging

All the animal protocols were assessed and approved by the Committee on Ethics of

Medicine, Navy Medical University (SMMU82030105). BALB/C nude female mice (certificate SCXK-2021-0013, weighing 18−20 g) were obtained from Changzhou Cavens Experimental Animal Co., Ltd. 100 μL of **AS-2F-NP-Cy3**, **AS-Cy3** and **CRO-Cy3** at a single dose of 5 μM were given to two groups of the MDA-MB-231 xenografts bearing BALB/C nude mice, respectively, by intravenous route (iv) via the tail vein. The nude mice were anesthetized and in vivo fluorescent imaging was carried out 4 and 8 h postinjection using Lumina XR imaging system. All the mice were sacrificed right after the last imaging. Tumors and major organs (hearts, lungs, livers, spleens, and kidneys) were collected and imaged with the in vivo imaging system (Lumina XR).

***In vivo* therapy**

The experimental procedures and the animal use and care protocols were approved by the Committee on Ethical Use of Animals of Second Military Medical University. BALB/C nude female mice (certificate SCXK-2007-0005, weighing 18−20 g) were obtained from Shanghai Experimental Animal Center, Chinese Academy of Sciences. The efficacy experiment in vivo was evaluated using the MDA-MB-231 tumor xenograft in mice. MDA-MB-231 cells (6×10^6 cells/animal) were subcutaneously into the flank area of the female nude mice (5−6 weeks old). When tumors reached an average volume of 150 mm^3 after implantation. nude mice were randomly divided into six groups. Five groups of nude mice were given intravenously with **AS** (10 μM/kg) and **AS-2F-NP** (10 μM/kg) in PBS and **FU** (1.3 mg/kg), **NP** (11.8 mg/kg)**, 2FU+NP** (2.6 mg/kg+11.8 mg/kg) in solution with formula (0.5% DMSO+5% ethanol + 0.5%

Tween 80 + ddH_2O) every other day with a volume of 100 μL per injection. The doses of **FU** and **NP** were equivalent to 10 μM/kg. The control group were given equal volume of PBS. During the treatment, we measured tumor size using vernier caliper and monitored body weight every four days. After 19 days treatment, the mice were killed. The major organs (hearts, lungs, livers, spleens, and kidneys) were dissected, collected and weighed. The tumors were taken out of the mice and pictured. The tumor volume was calculated by this formula, volume = $AB^2/2$. A and B are the length and width dimension of the tumor, respectively. Data were analyzed by 1-way ANOVA test. P level < 0.05 was considered statistically significant.

Histology

After 30 days post-treatment, the heart, liver, spleen, lung, kidney, and tumors of all treatment groups were dissected and fixed with 4% paraformaldehyde. The tissue of organs was sliced and stained by Bios Biological Company.

Statistical analysis

All the data were presented as mean ± sd. Student's *t*-test or 1-way analyses of variance (ANOVA) were performed in the evaluation of statistical. P level < 0.05 was considered statistically significant.

The spectra of the synthesized compounds

Compound 6

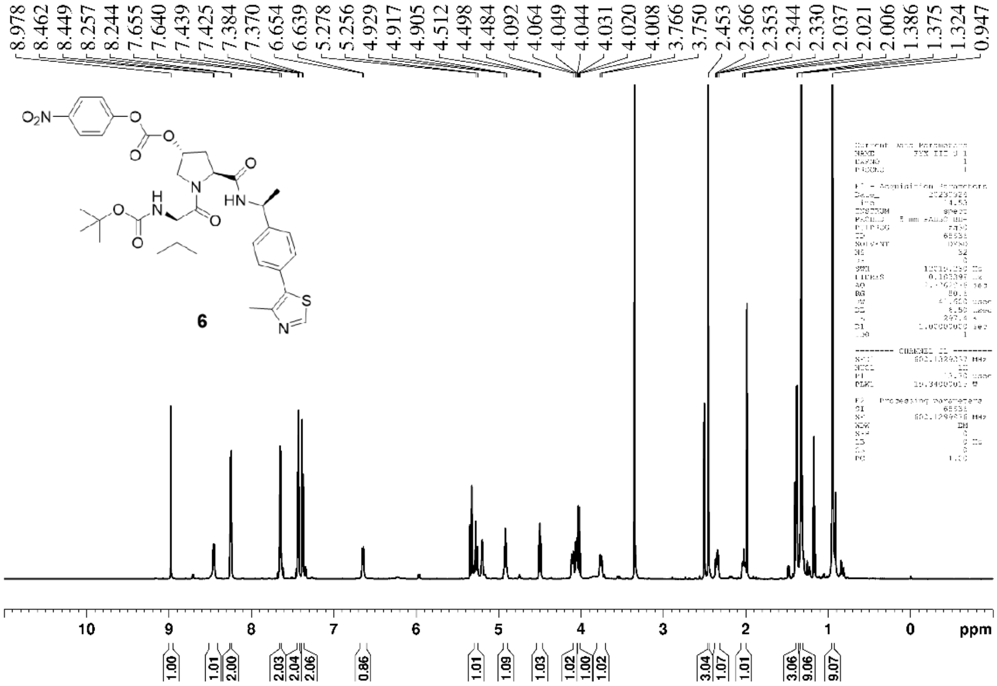

Compound 7

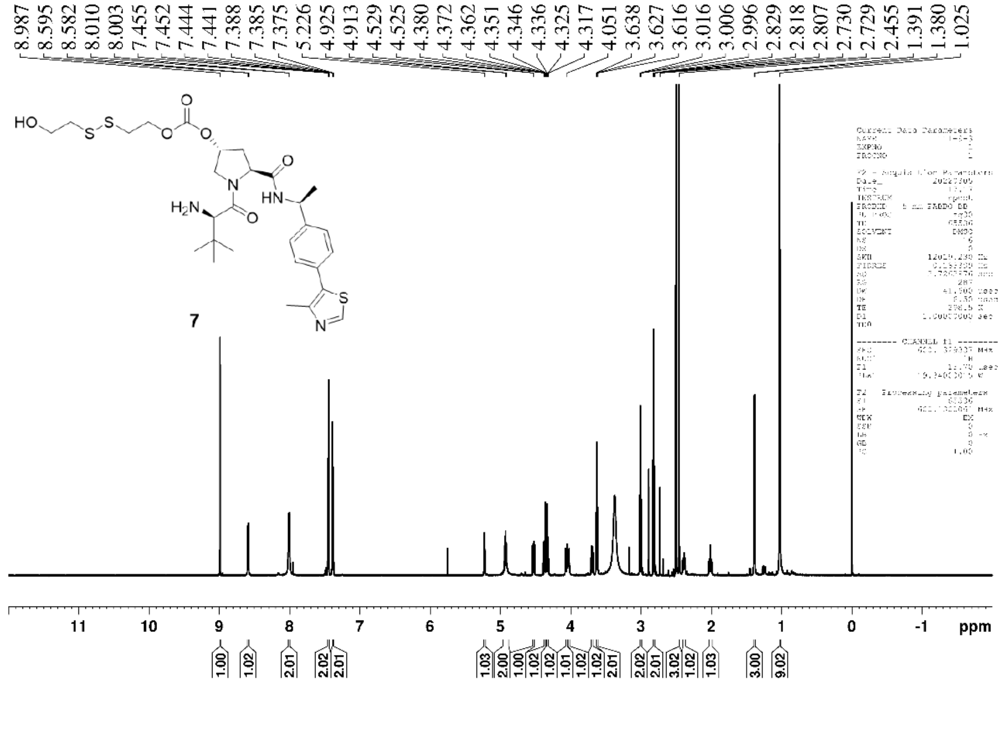

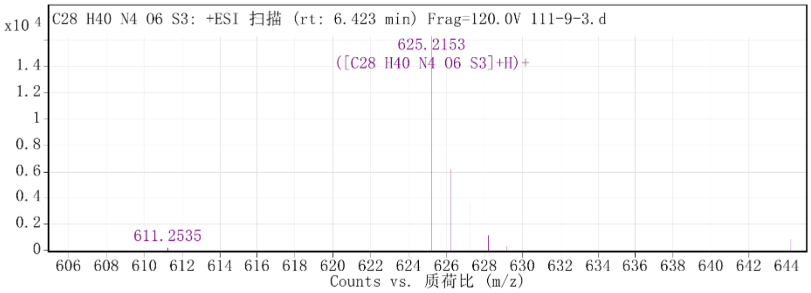
C28 H40 N4 O6 S3: +ESI 扫描 (rt: 6.423 min) Frag=120.0V 111-9-3.d
625.2153
([C28 H40 N4 O6 S3]+H)+
611.2535
Counts vs. 质荷比 (m/z)

Compound 8

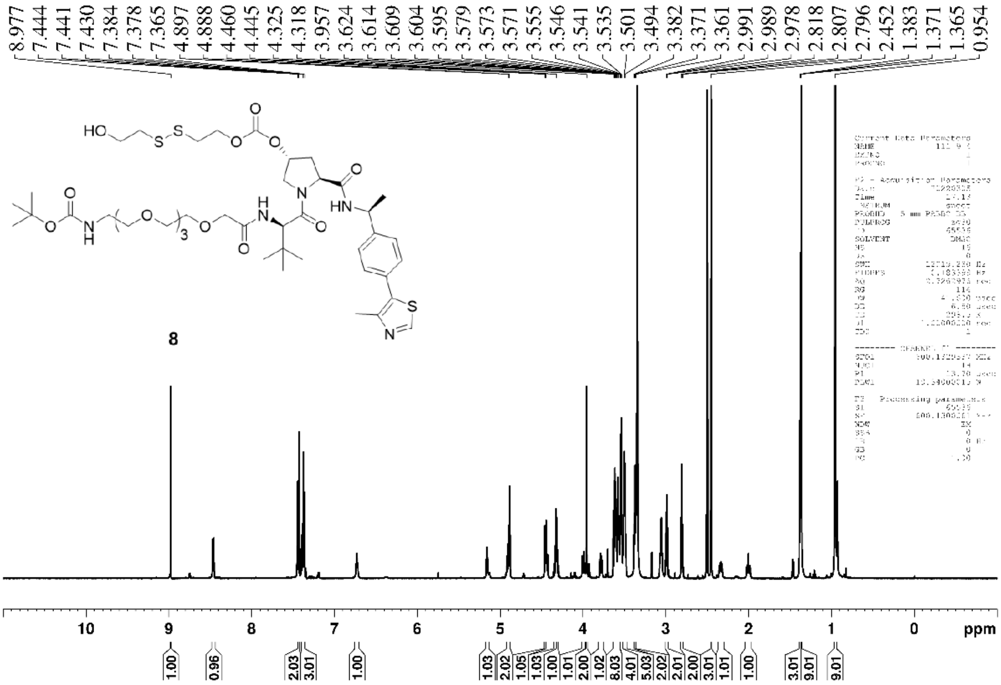
8
ppm

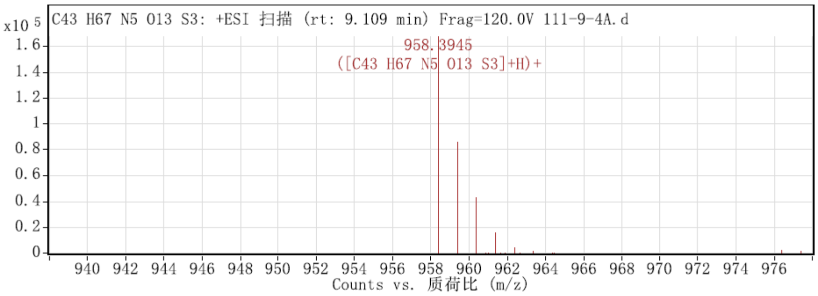
C43 H67 N5 O13 S3: +ESI 扫描 (rt: 9.109 min) Frag=120.0V 111-9-4A.d
958.3945
([C43 H67 N5 O13 S3]+H)+
Counts vs. 质荷比 (m/z)

Compound 9

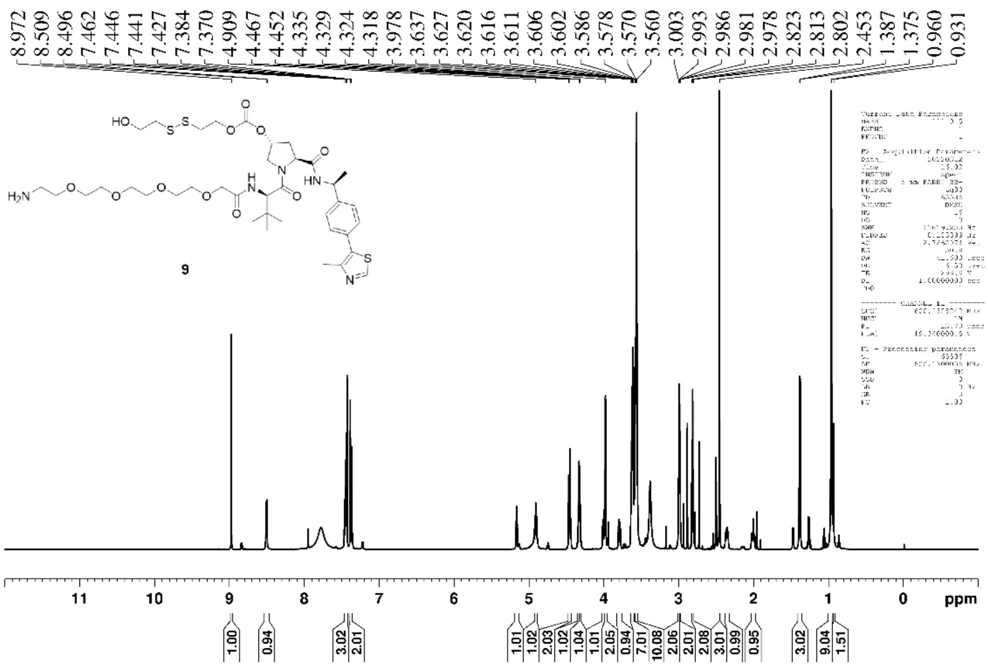

Compound 10

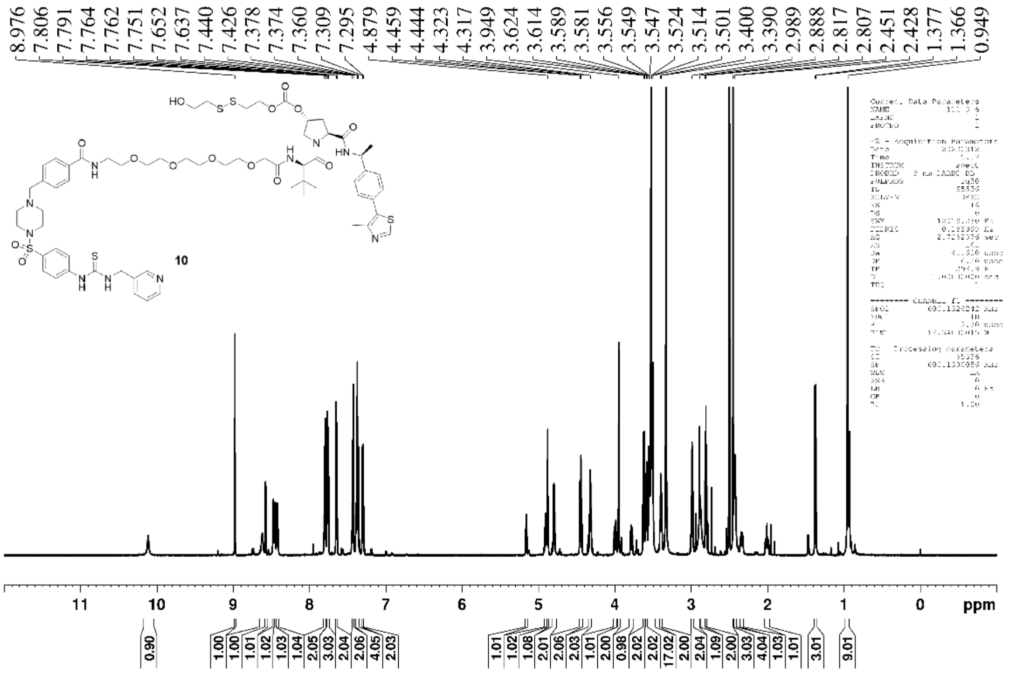
8.976 7.806 7.791 7.764 7.762 7.751 7.652 7.637 7.440 7.426 7.378 7.374 7.360 7.309 7.295 4.879 4.459 4.444 4.323 4.317 3.949 3.624 3.614 3.589 3.581 3.556 3.549 3.547 3.524 3.514 3.501 3.400 3.390 2.989 2.888 2.817 2.807 2.451 2.428 1.377 1.366 0.949
10
11 10 9 8 7 6 5 4 3 2 1 0 ppm
0.90 1.00 1.00 1.01 1.02 1.03 1.04 2.05 3.03 2.04 2.06 4.05 2.03 1.01 1.02 1.08 2.01 2.06 2.03 1.01 2.00 0.98 2.02 2.02 17.02 2.00 2.04 1.09 2.00 3.03 4.04 1.03 1.01 3.01 9.01

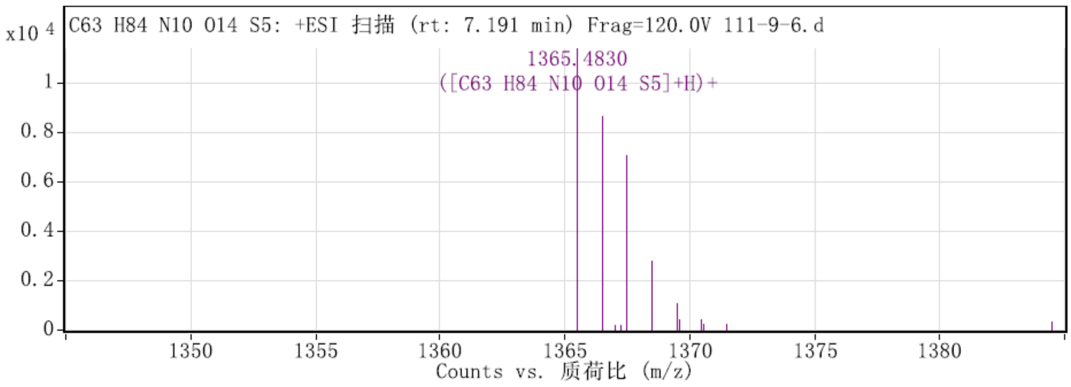
x10 4
C63 H84 N10 O14 S5: +ESI 扫描 (rt: 7.191 min) Frag=120.0V 111-9-6.d
1365.4830
([C63 H84 N10 O14 S5]+H)+
1
0.8
0.6
0.4
0.2
0
1350 1355 1360 1365 1370 1375 1380
Counts vs. 质荷比 (m/z)

Compound 11

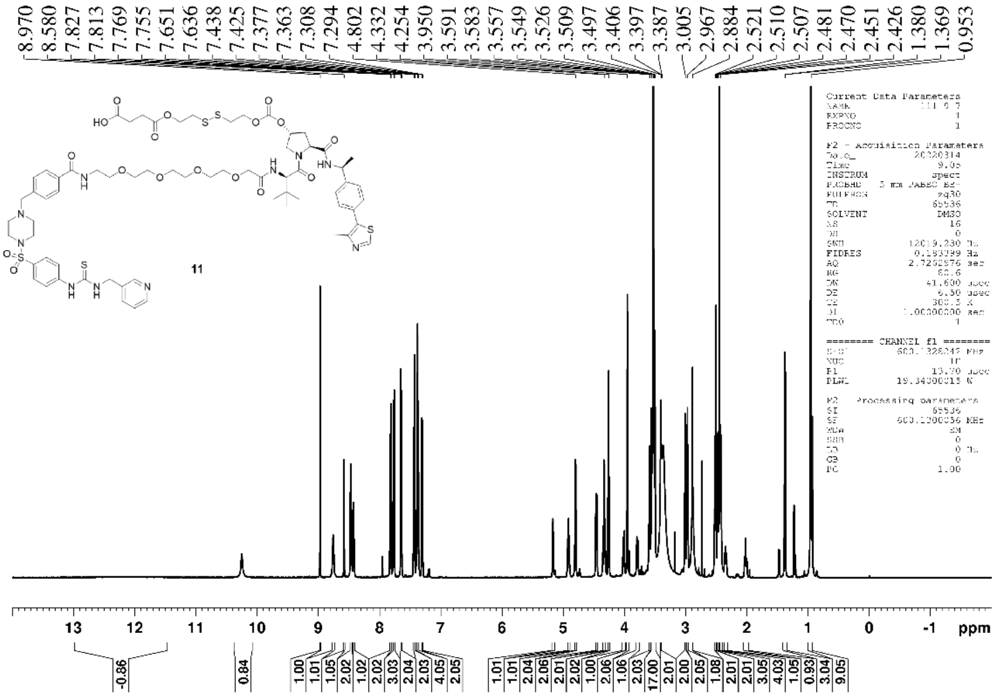

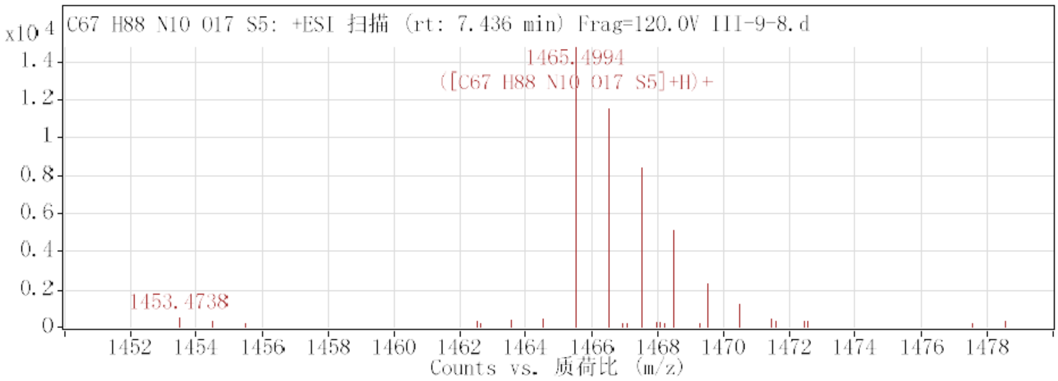

C67 H88 N10 O17 S5: +ESI 扫描 (rt: 7.436 min) Frag=120.0V 111-9-8.d
1465.4994
([C67 H88 N10 O17 S5]+H)+
1453.4738
Counts vs. 质荷比 (m/z)

Compound 13

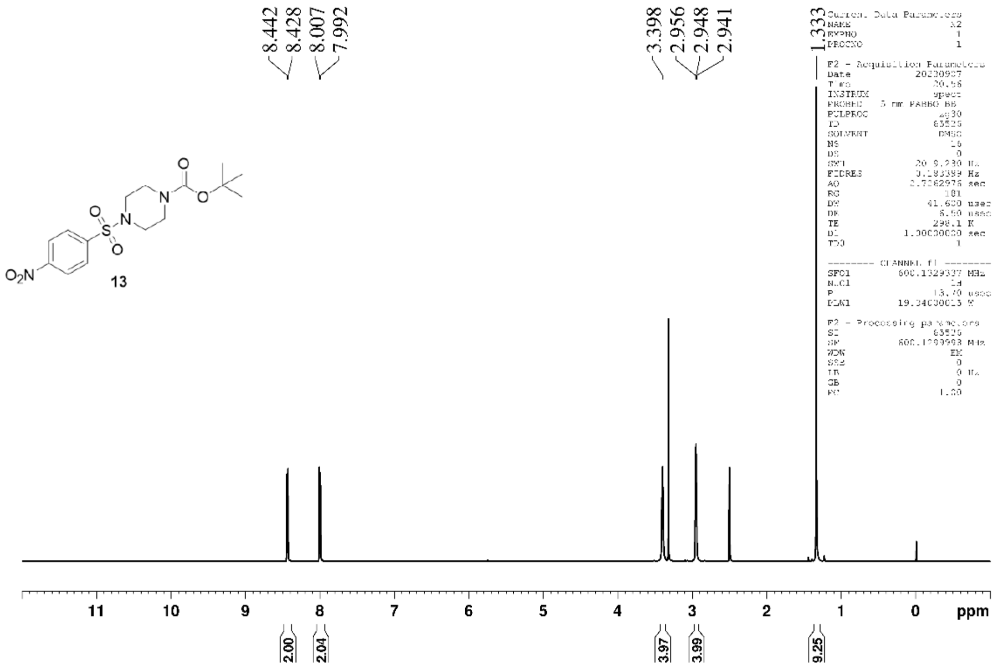

8.442
8.428
8.007
7.992
3.398
2.956
2.948
2.941
1.333
13
11 10 9 8 7 6 5 4 3 2 1 0 ppm
2.00
2.04
3.97
3.99
9.25

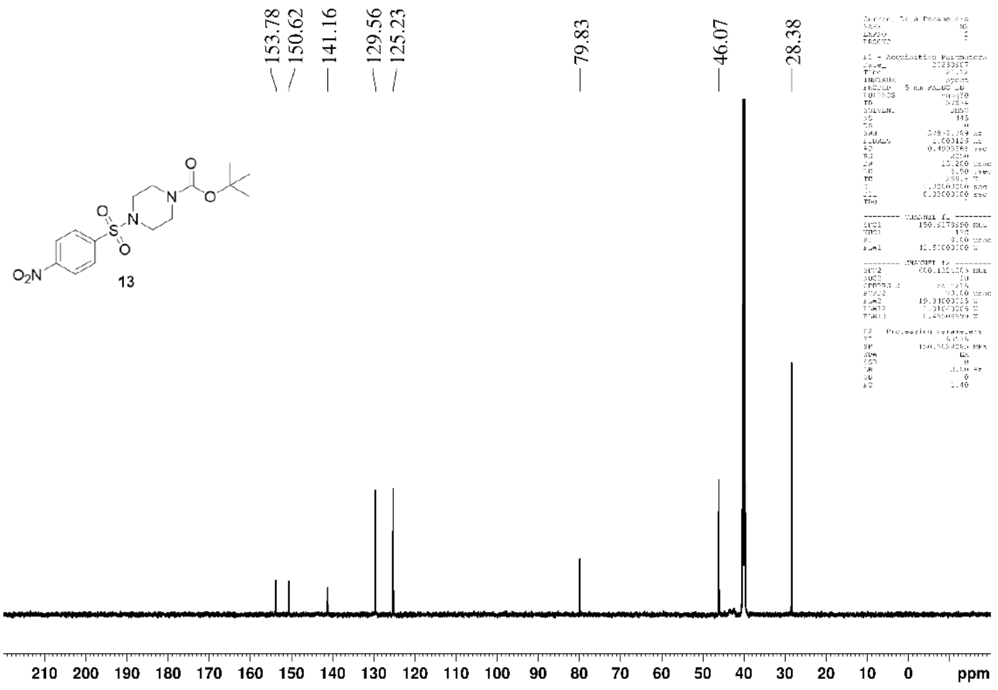

153.78
150.62
141.16
129.56
125.23
79.83
46.07
28.38
13
210 200 190 180 170 160 150 140 130 120 110 100 90 80 70 60 50 40 30 20 10 0 ppm

Compound 14

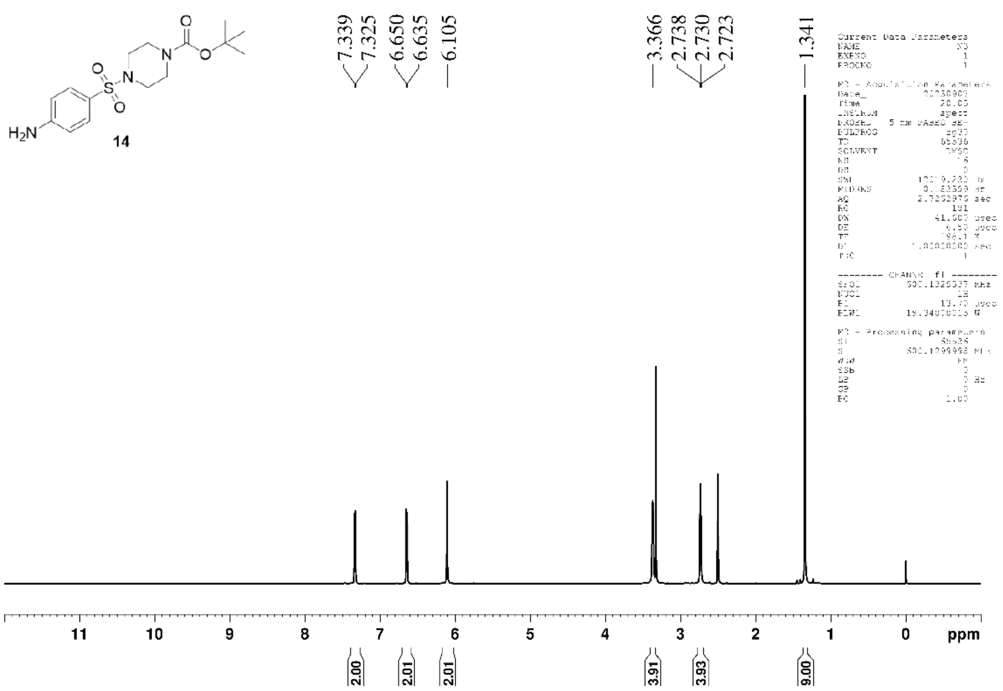

14
7.339
7.325
6.650
6.635
6.105
3.366
2.738
2.730
2.723
1.341
2.00
2.01
2.01
3.91
3.93
9.00
ppm

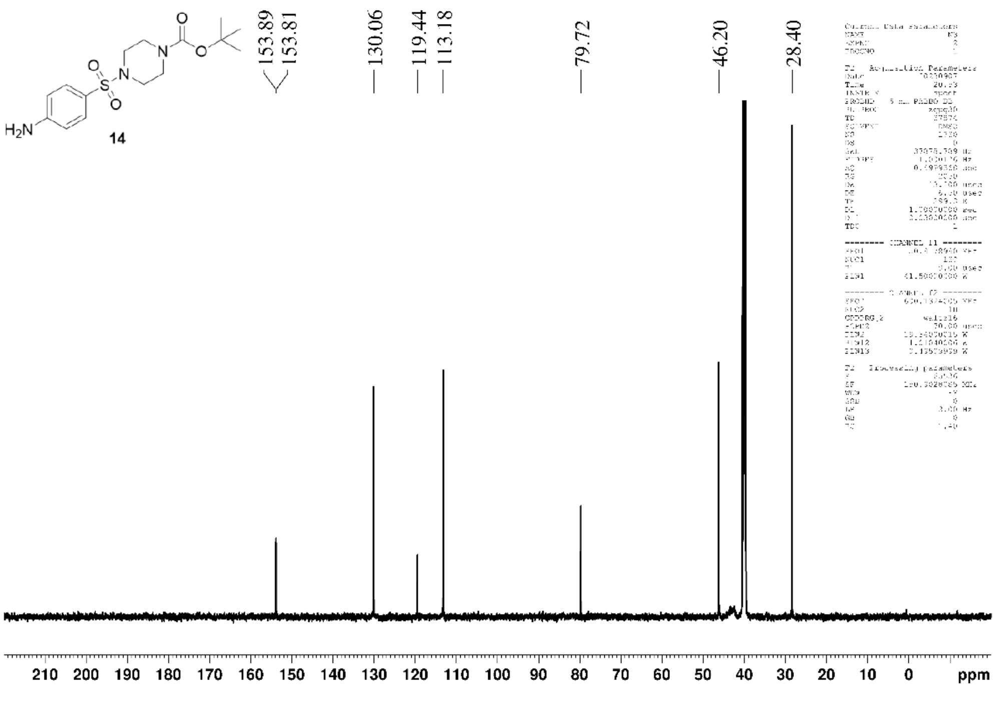

14
153.89
153.81
130.06
119.44
113.18
79.72
46.20
28.40
ppm

Compound 15

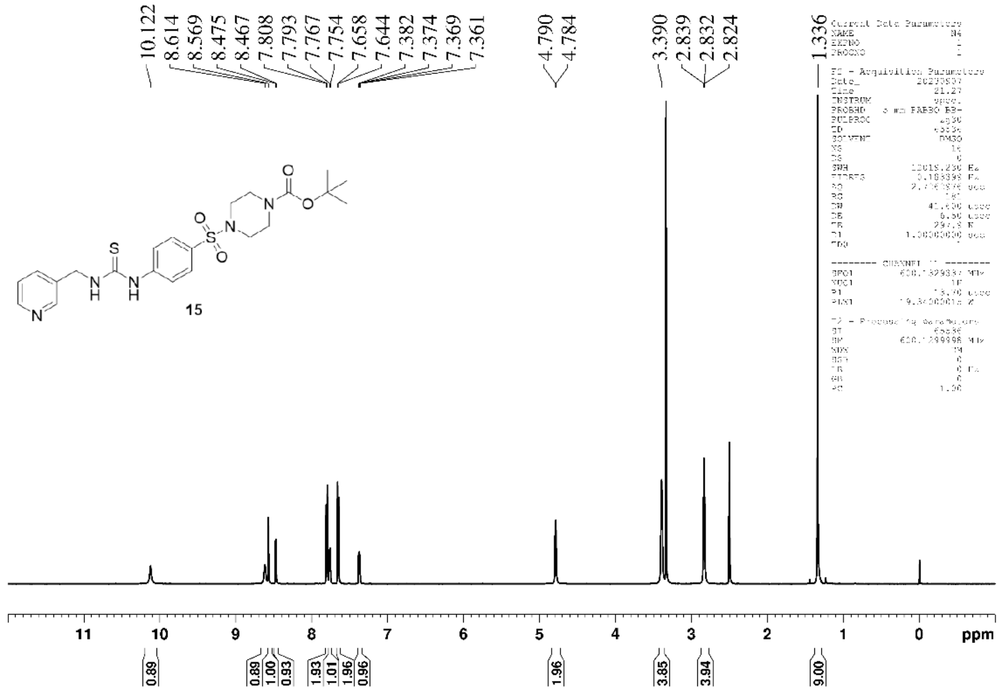
10.122
8.614
8.569
8.475
8.467
7.808
7.793
7.767
7.754
7.658
7.644
7.382
7.374
7.369
7.361
4.790
4.784
3.390
2.839
2.832
2.824
1.336
15
11 10 9 8 7 6 5 4 3 2 1 0 ppm
0.89
0.89
1.00
0.93
1.93
1.01
1.96
0.96
1.96
3.85
3.94
9.00

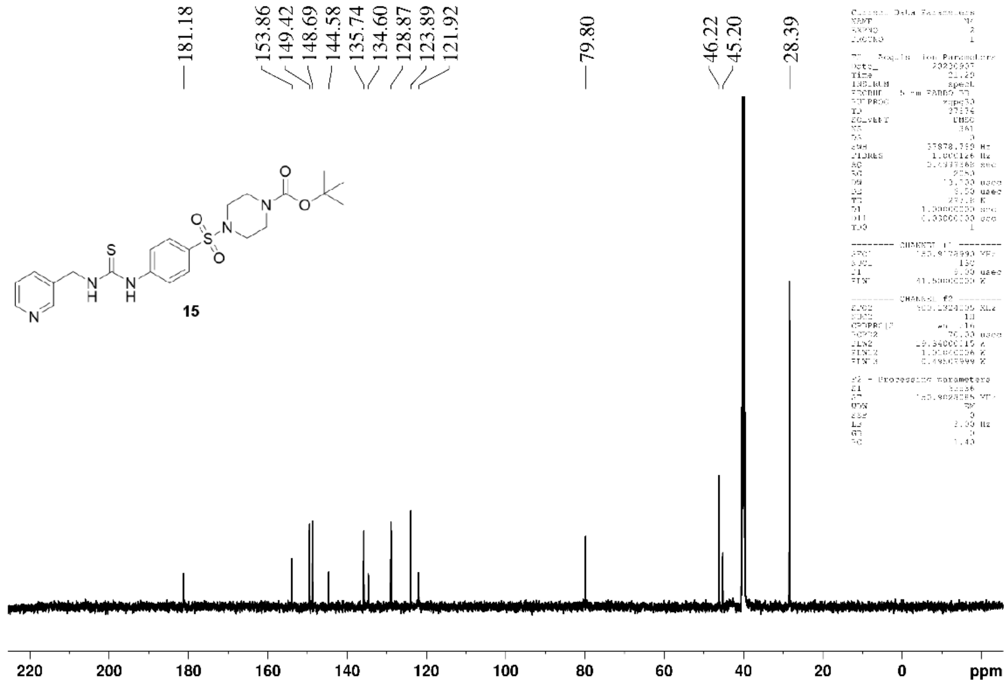
181.18
153.86
149.42
148.69
144.58
135.74
134.60
128.87
123.89
121.92
79.80
46.22
45.20
28.39
15
220 200 180 160 140 120 100 80 60 40 20 0 ppm

Compound 16

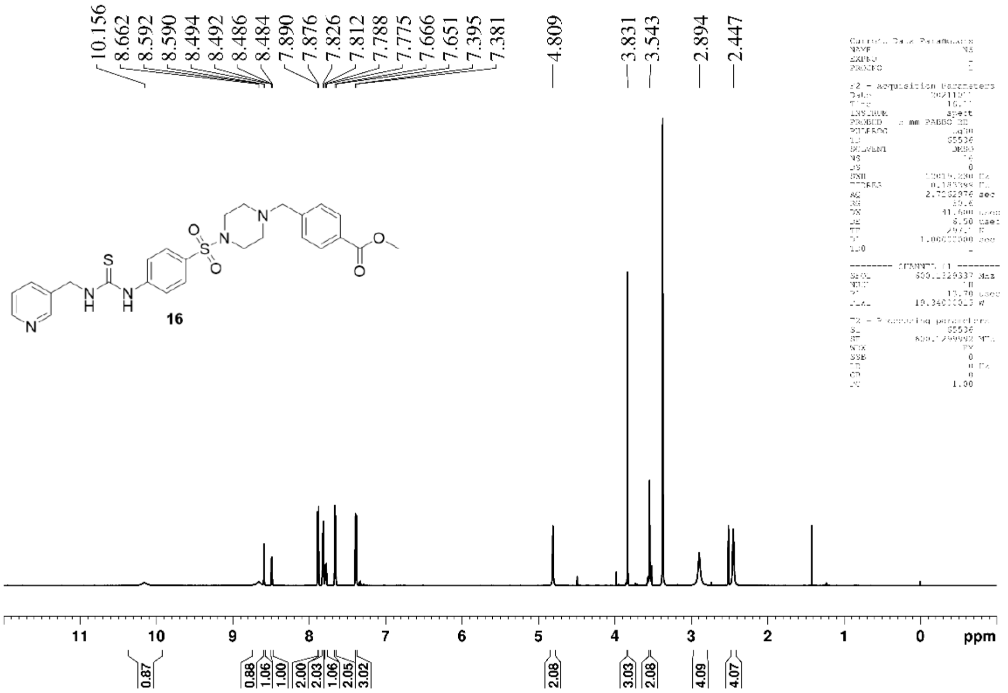
10.156
8.662
8.592
8.590
8.494
8.492
8.486
8.484
7.890
7.876
7.826
7.812
7.788
7.775
7.666
7.651
7.395
7.381
4.809
3.831
3.543
2.894
2.447
16
ppm
0.87
0.88
1.06
1.00
2.00
2.03
1.06
2.05
3.02
2.08
3.03
2.08
4.09
4.07

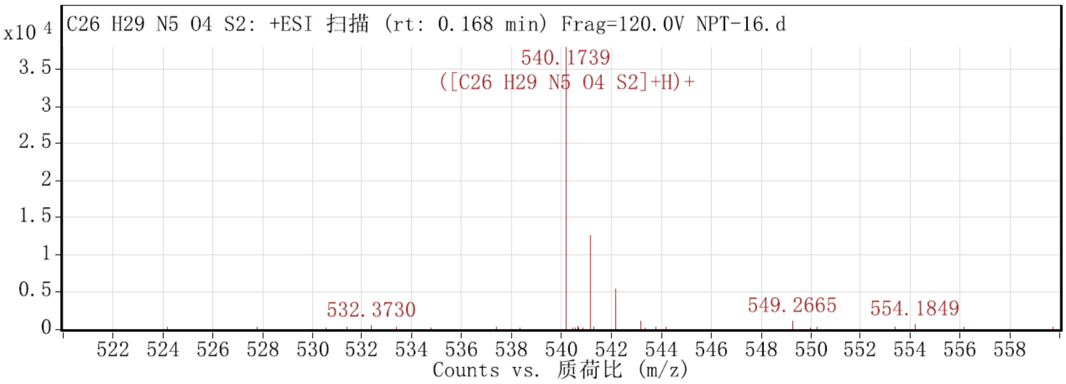
C26 H29 N5 O4 S2: +ESI 扫描 (rt: 0.168 min) Frag=120.0V NPT-16.d
x10 4
540.1739
([C26 H29 N5 O4 S2]+H)+
532.3730
549.2665
554.1849
Counts vs. 质荷比 (m/z)

Compound 1S

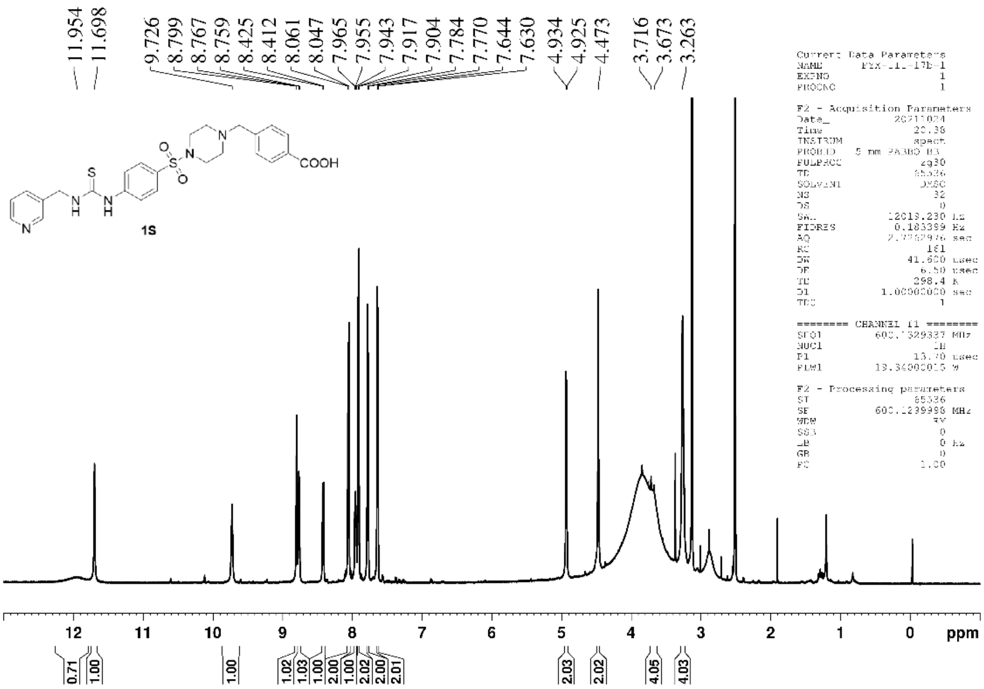

11.954
11.698
9.726
8.799
8.767
8.759
8.425
8.412
8.061
8.047
7.965
7.955
7.943
7.917
7.904
7.784
7.770
7.644
7.630
4.934
4.925
4.473
3.716
3.673
3.263
COOH
1S
ppm

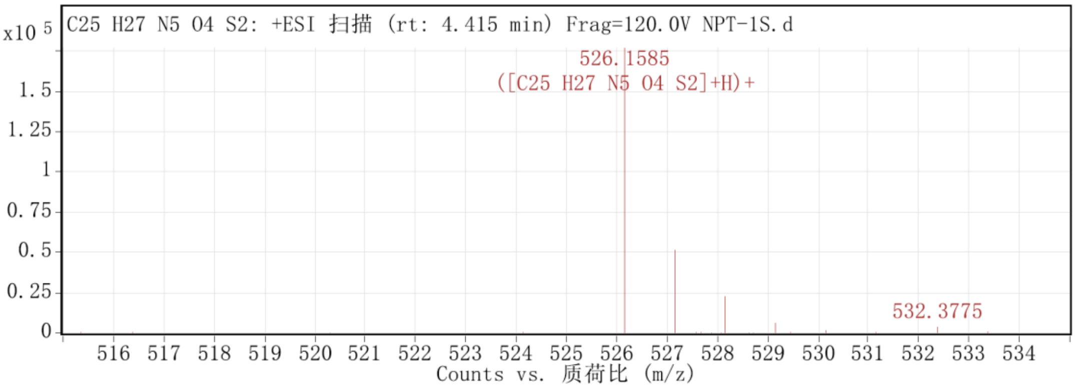

C25 H27 N5 O4 S2: +ESI 扫描 (rt: 4.415 min) Frag=120.0V NPT-1S.d
x10 5
526.1585
([C25 H27 N5 O4 S2]+H)+
532.3775
Counts vs. 质荷比 (m/z)

Table S2. The characteristics of title compounds

Compounds	Sequence	MW
**AS1411 (AS)**	5' GGTGGTGGTGGTTGTGGTGGTGGTGG 3'	8272.36
**AS-2Fdu-Fam**	5'amino-/i5F-dU 5F-dU/TTTTTTGGTGGTGGTGGTTGTGGTGGTGGTGG-Fam 3'	11462.56
**AS-2Fdu-Cy3**	5'amino-/i5F-dU 5F-dU/TTTTTTGGTGGTGGTGGTTGTGGTGGTGGTGG-Cy3 3'	11537.66
**AS-2Fdu-NH_2**	5'amino-/i5F-dU 5F-dU/TTTTTTGGTGGTGGTGGTTGTGGTGGTGGTGG 3'	10893.06
**CRO-2Fdu-NH_2**	5'amino-/i5F-dU 5F-dU/TTTTTTCCTCCTCCTCCTTCTCCTCCTCCTCC 3'	10212.65
**AS-Fam**	5' GGTGGTGGTGGTTGTGGTGGTGGTGG-Fam 3'	8841.86
**AS-Cy3**	5' GGTGGTGGTGGTTGTGGTGGTGGTGG-Cy3 3'	8916.96
**CRO-Fam**	5' CCTCCTCCTCCTTCTCCTCCTCCTCC-Fam 3'	8161.45
**CRO-Cy3**	5' CCTCCTCCTCCTTCTCCTCCTCCTCC-Cy3 3'	8236.55
**AS-2F-NP**	PROTAC-5'amino-/i5F-dU 5F-dU/TTTTTTGGTGGTGGTGGTTGTGGTGGTGGTGG 3'	12339.5
**CRO-2F-NP**	PROTAC-5'amino-/i5F-dU 5F-dU/TTTTTTCCTCCTCCTCCTTCTCCTCCTCCTCC 3'	11658.8
**AS-2F-NP-Fam**	PROTAC-5'amino-/i5F-dU 5F-dU/TTTTTTGGTGGTGGTGGTTGTGGTGGTGGTGG-Fam 3'	12908.45
**AS-2F-NP-Cy3**	PROTAC-5'amino-/i5F-dU 5F-dU/TTTTTTGGTGGTGGTGGTTGTGGTGGTGGTGG-Cy3 3'	12983.48

AS

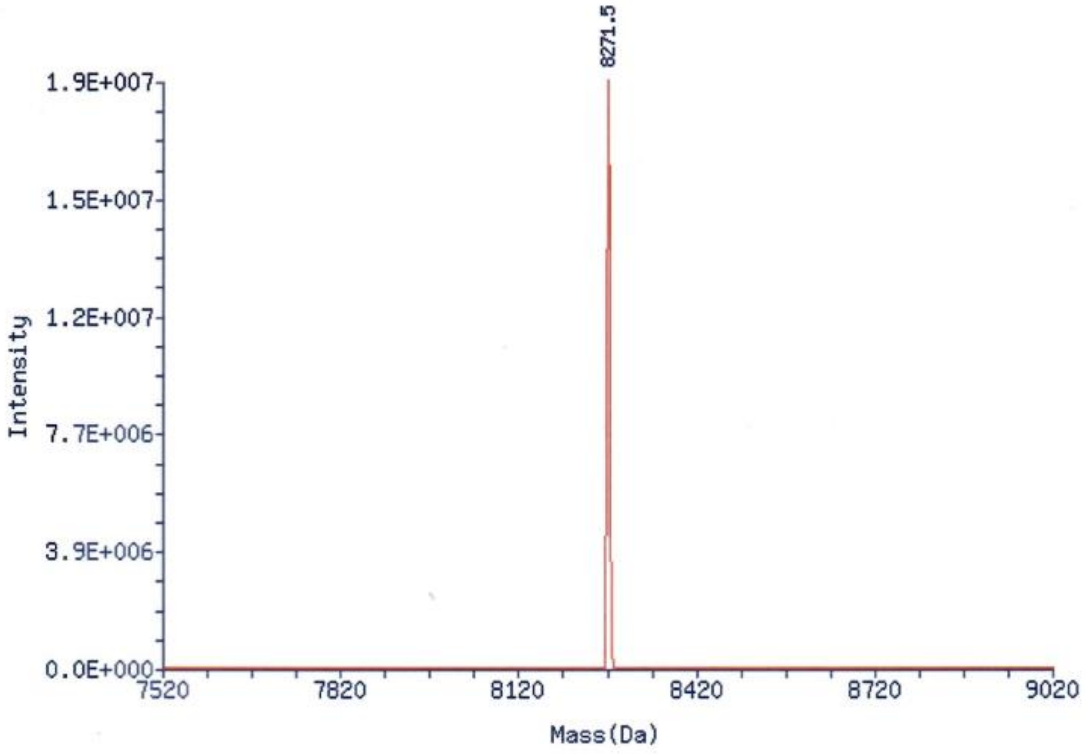

Figure S17. Mass analysis of **AS** by Sangon (Shanghai). Calculated molecular weight: 8272.36, Found: 8271.5.

AS-2Fdu-Fam

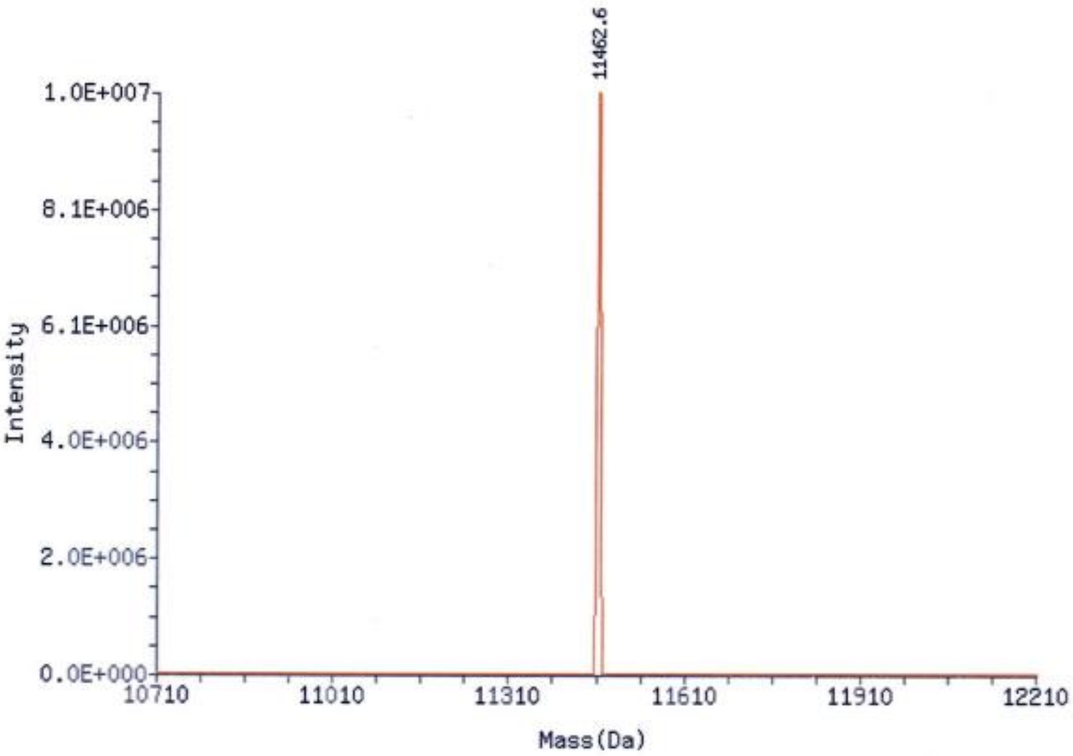

Figure S18. Mass analysis of **AS-2Fdu-Fam** by Sangon (Shanghai). Calculated molecular weight: 11462.56, Found: 11462.6.

AS-2Fdu-Cy3

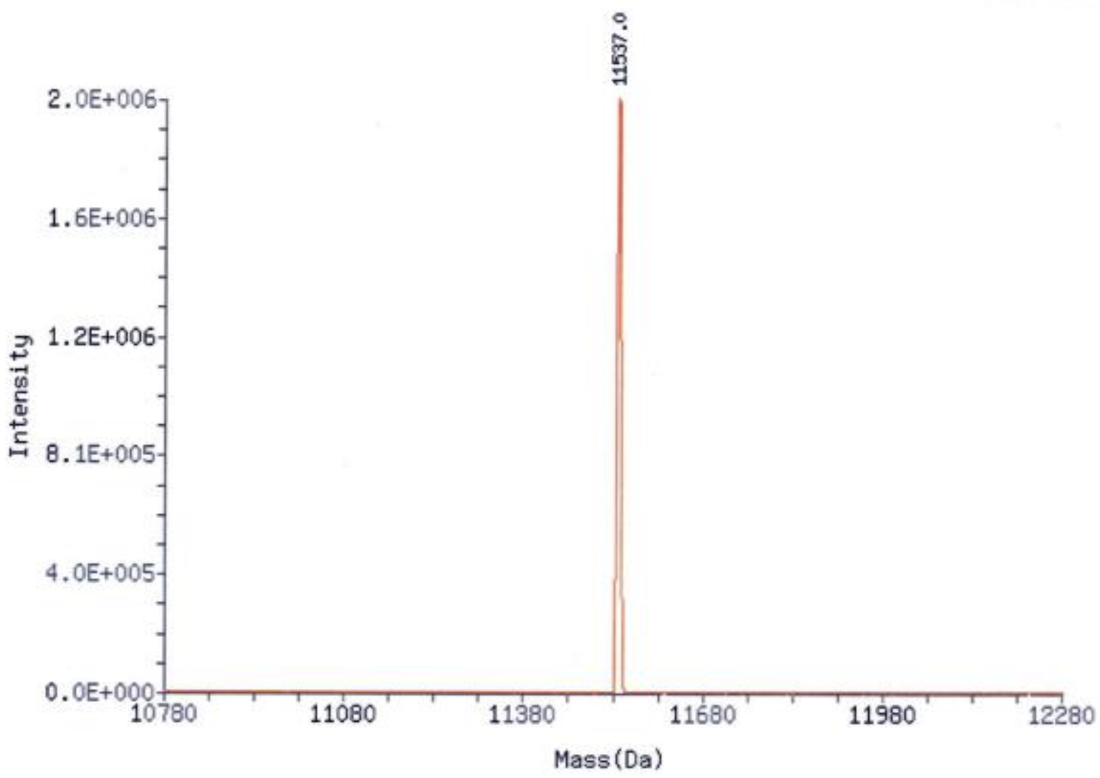

Figure S19. Mass analysis of **AS-2Fdu-Cy3** by Sangon (Shanghai). Calculated molecular weight: 11537.66, Found: 11537.0.

AS-2Fdu-NH_2

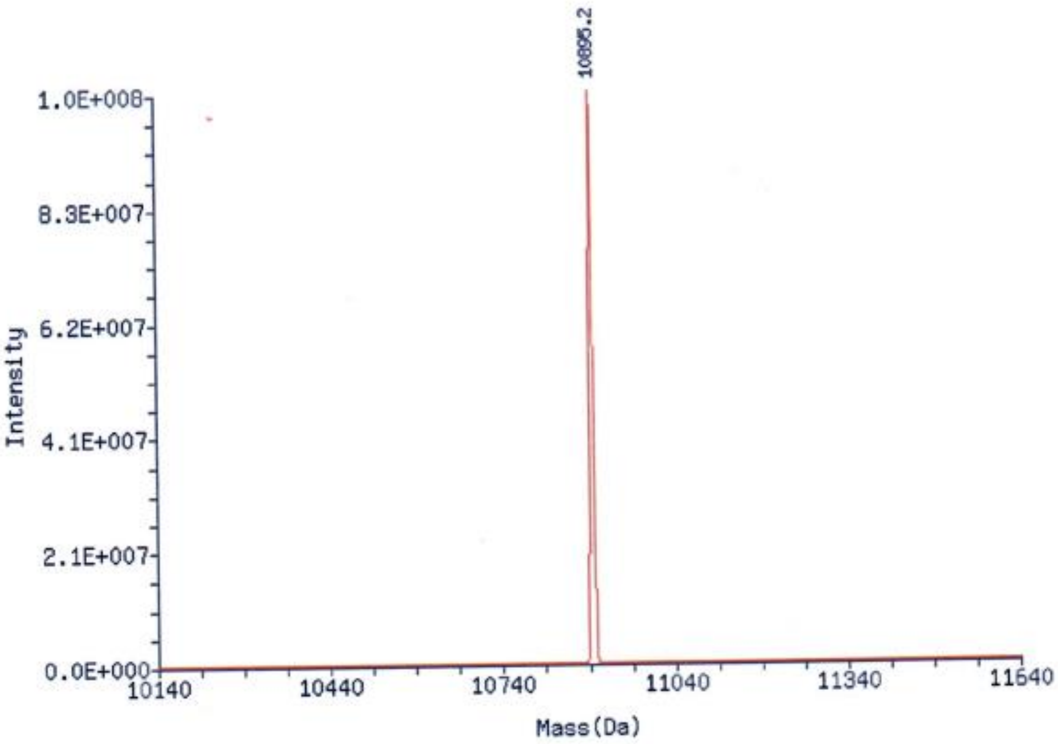

Figure S20. Mass analysis of **AS-2Fdu-NH_2** by Sangon (Shanghai). Calculated molecular weight: 10893.06, Found: 10895.2.

CRO-2Fdu-NH_2

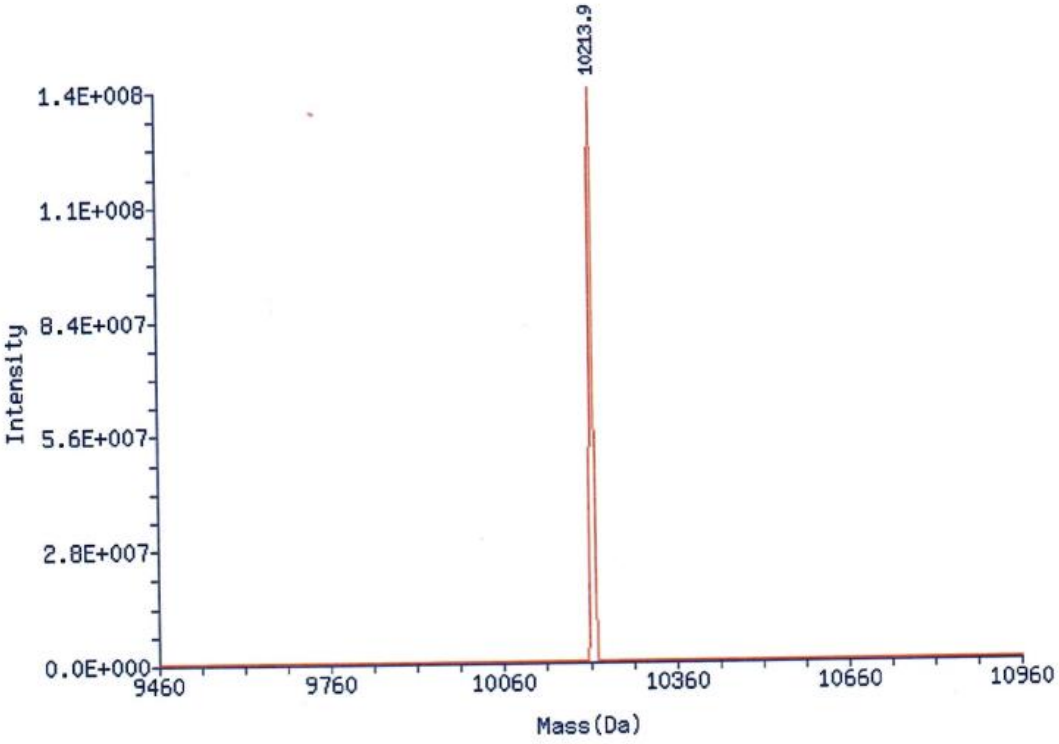

Figure S21. Mass analysis of **CRO-2Fdu-NH_2** by Sangon (Shanghai). Calculated molecular weight: 10212.65, Found: 10213.9.

AS-Fam

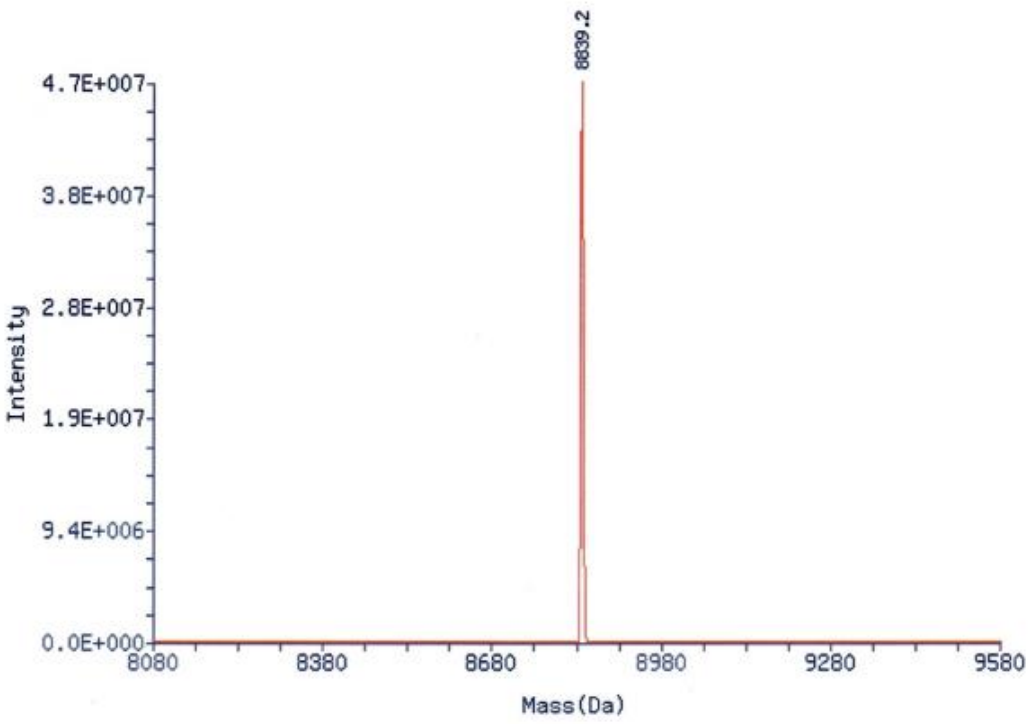

Figure S22. Mass analysis of **AS-Fam** by Sangon (Shanghai). Calculated molecular weight: 8841.86, Found: 8839.2.

AS-Cy3

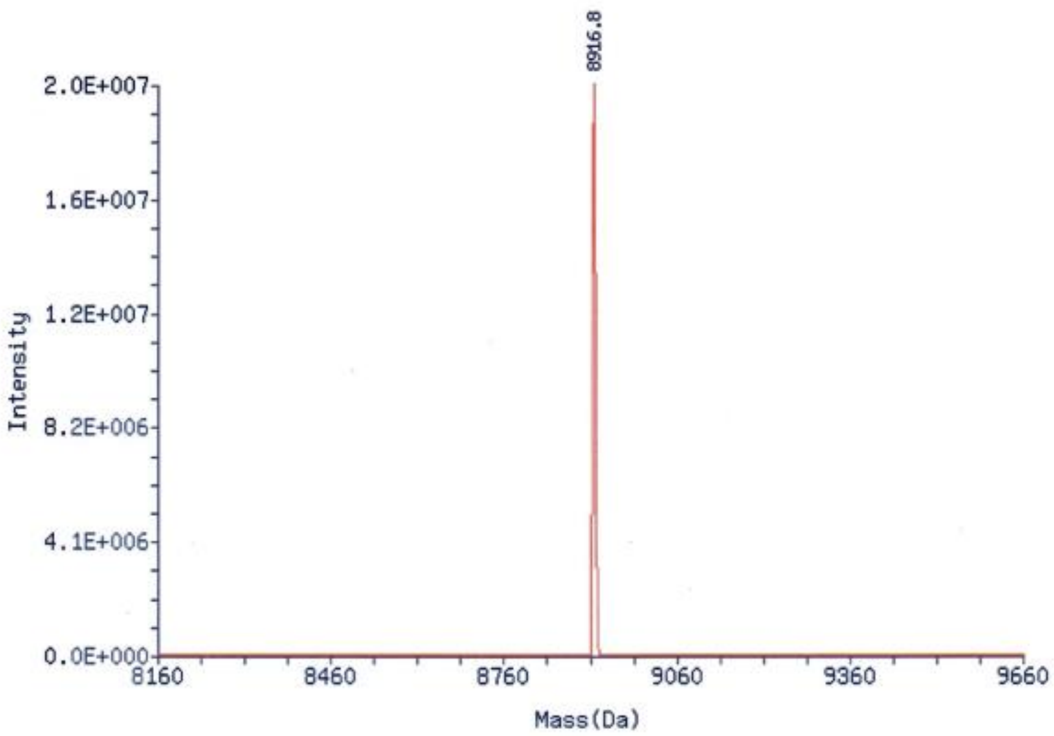

Figure S23. Mass analysis of **AS-Cy3** by Sangon (Shanghai). Calculated molecular weight: 8916.96, Found: 8916.8.

CRO-Fam

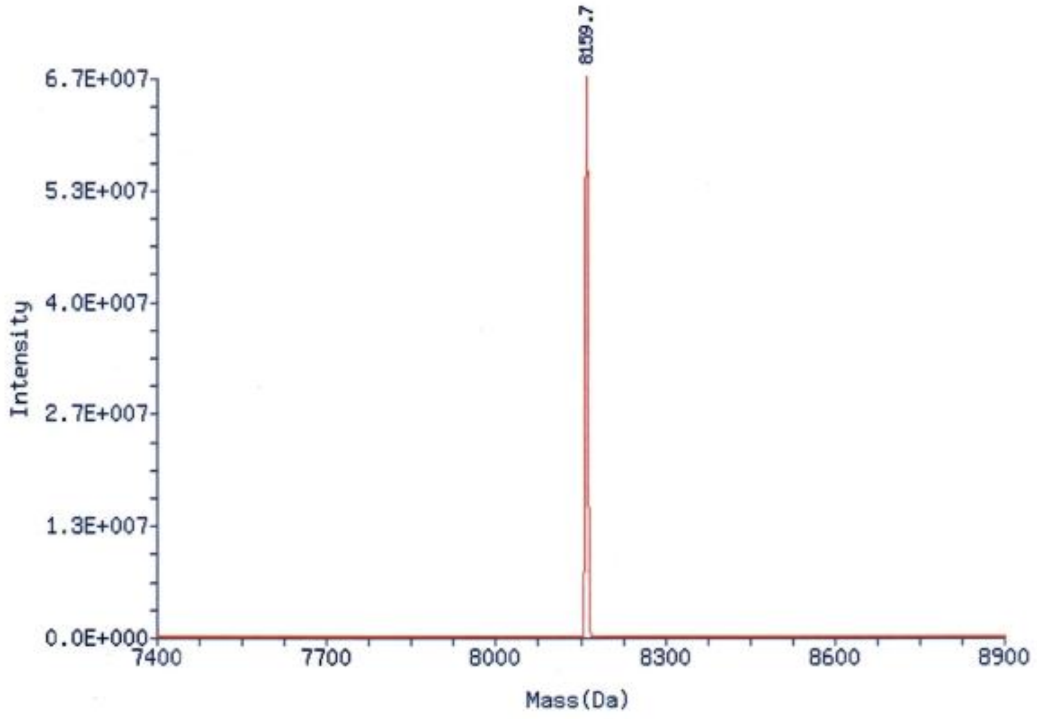

Figure S24. Mass analysis of **CRO-Fam** by Sangon (Shanghai). Calculated molecular weight: 8161.45, Found: 8159.7.

CRO-Cy3

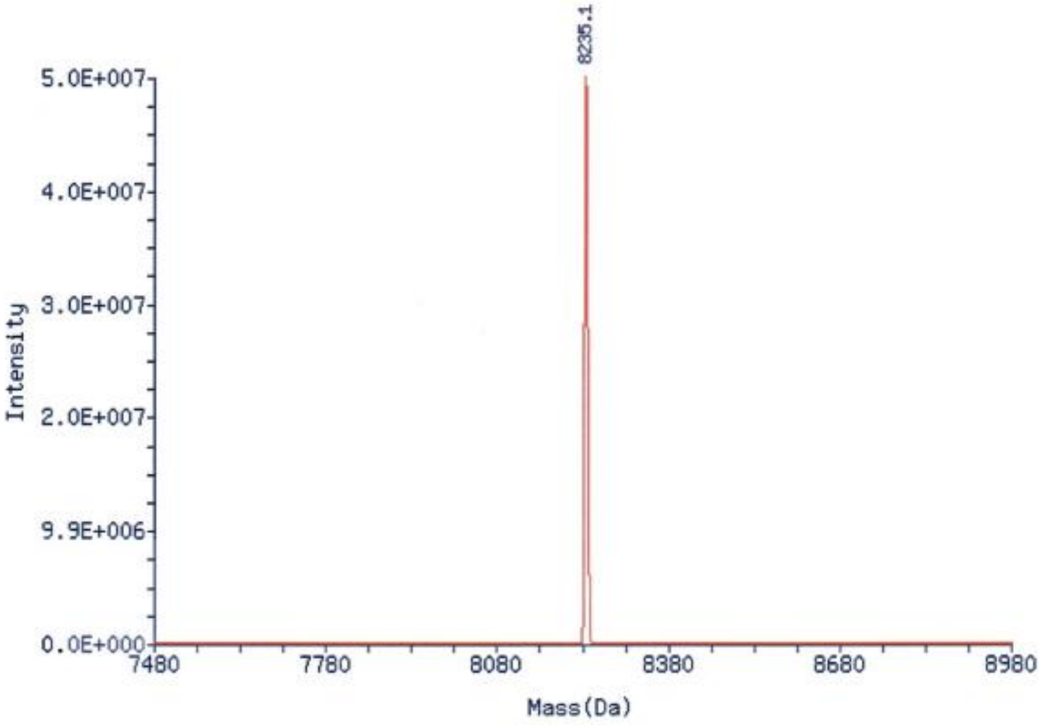

Figure S25. Mass analysis of **CRO-Cy3** by Sangon (Shanghai). Calculated molecular weight: 8236.55, Found: 8235.1.

AS-2F-NP

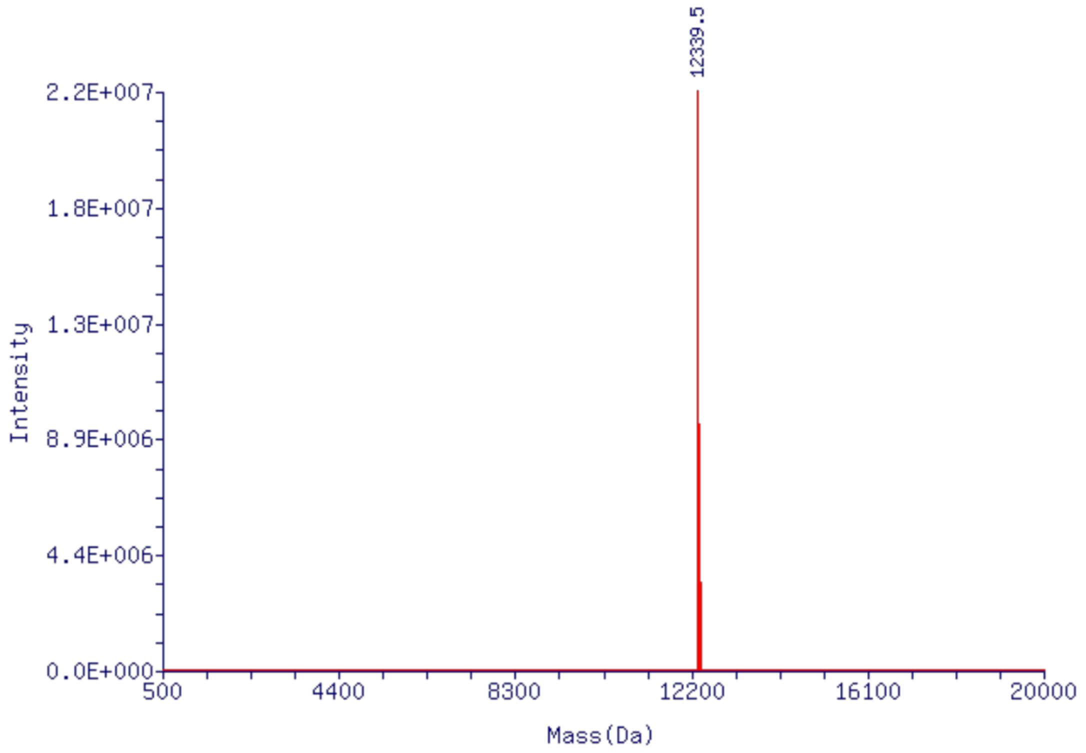

Purity: 95.5%

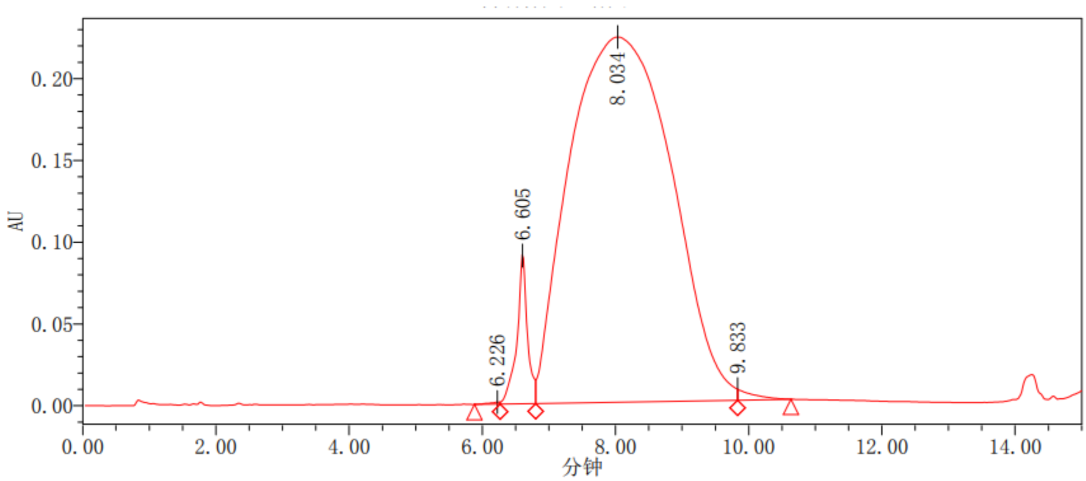

峰结果

	名称	保留时间 (分钟)	面积 (微伏*秒)	高度 (微伏)	% 面积
1		6.226	13890	1229	0.06
2		6.605	1007169	91127	4.03
3		8.034	23867791	223298	95.48
4		9.833	108001	6693	0.43

CRO-2F-NP

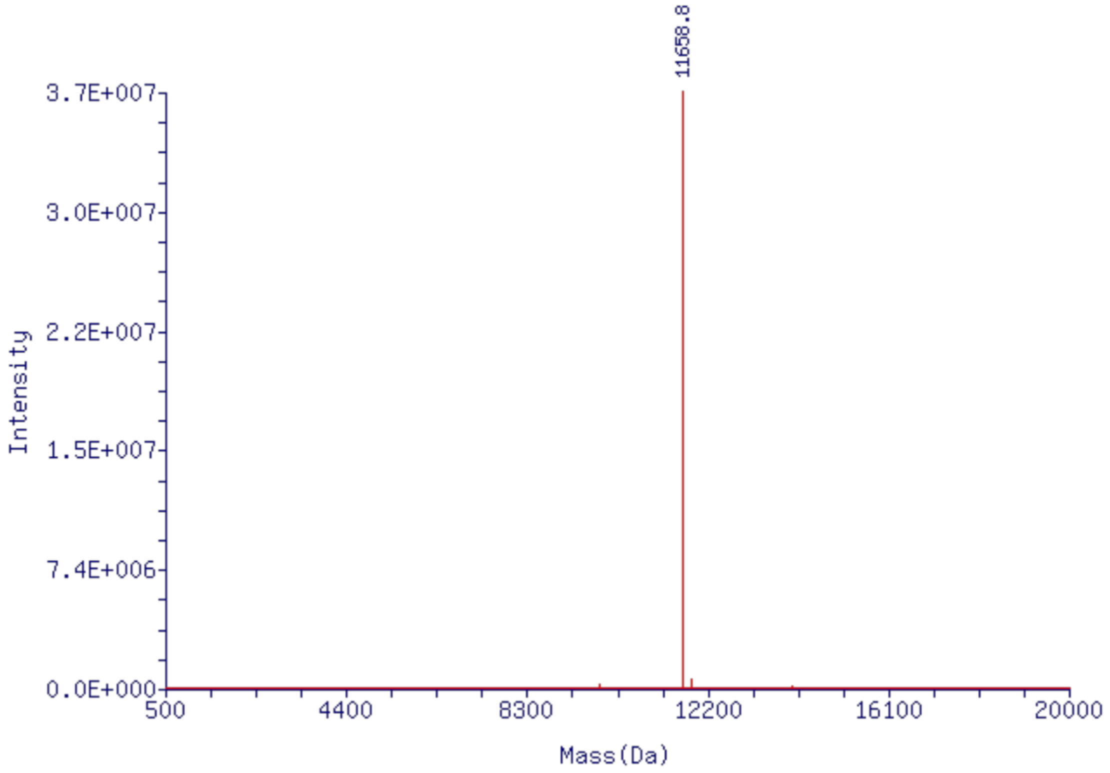

Purity: 97.4%

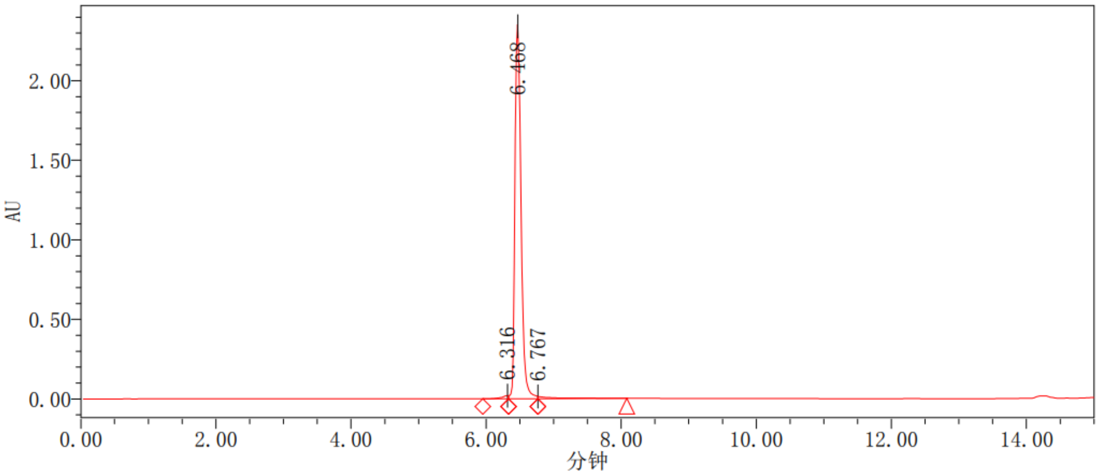

峰结果

	名称	保留时间 (分钟)	面积 (微伏*秒)	高度 (微伏)	% 面积
1		6.316	127667	19082	0.84
2		6.468	14880820	2356882	97.41
3		6.767	268534	14119	1.76

AS-2F-NP-Fam

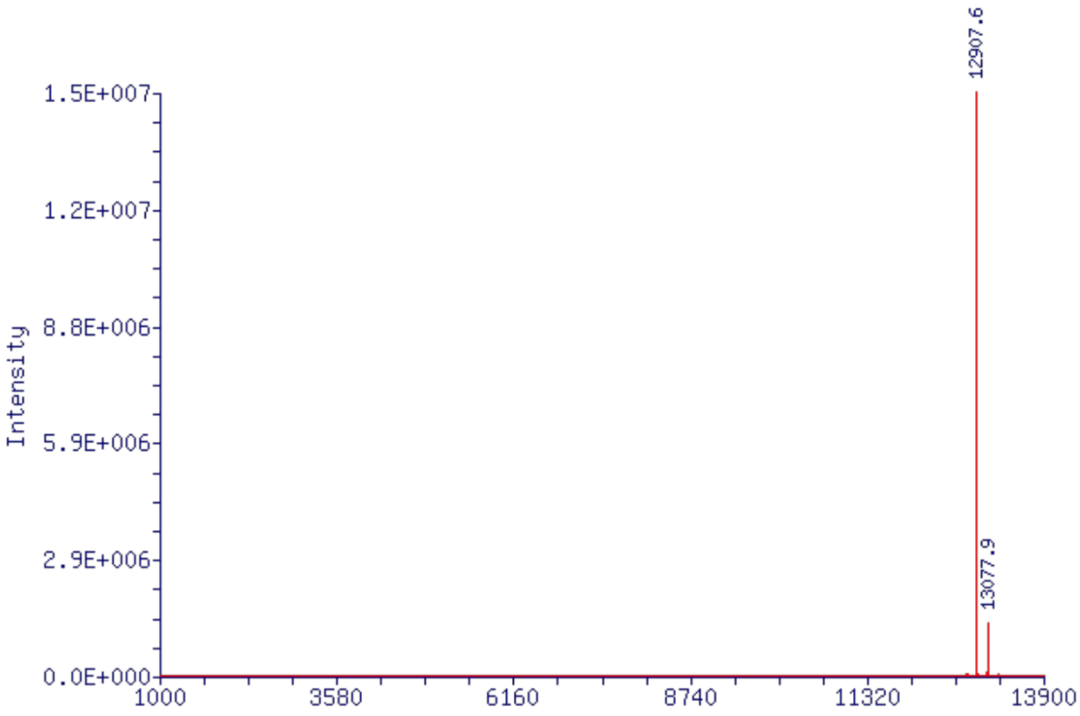

Purity: 99.1%

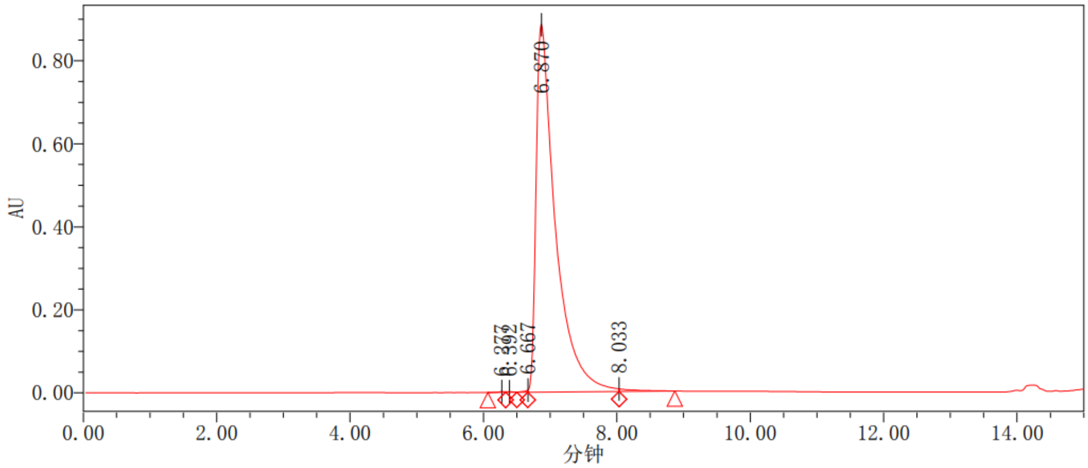

峰结果

	名称	保留时间<br>(分钟)	面积<br>(微伏*秒)	高度<br>(微伏)	% 面积
1		6. 277	11788	1896	0. 07
2		6. 392	12766	1550	0. 07
3		6. 667	20488	5095	0. 11
4		6. 870	17835354	887800	99. 16
5		8. 033	106150	6158	0. 59

AS-2F-NP-Cy3

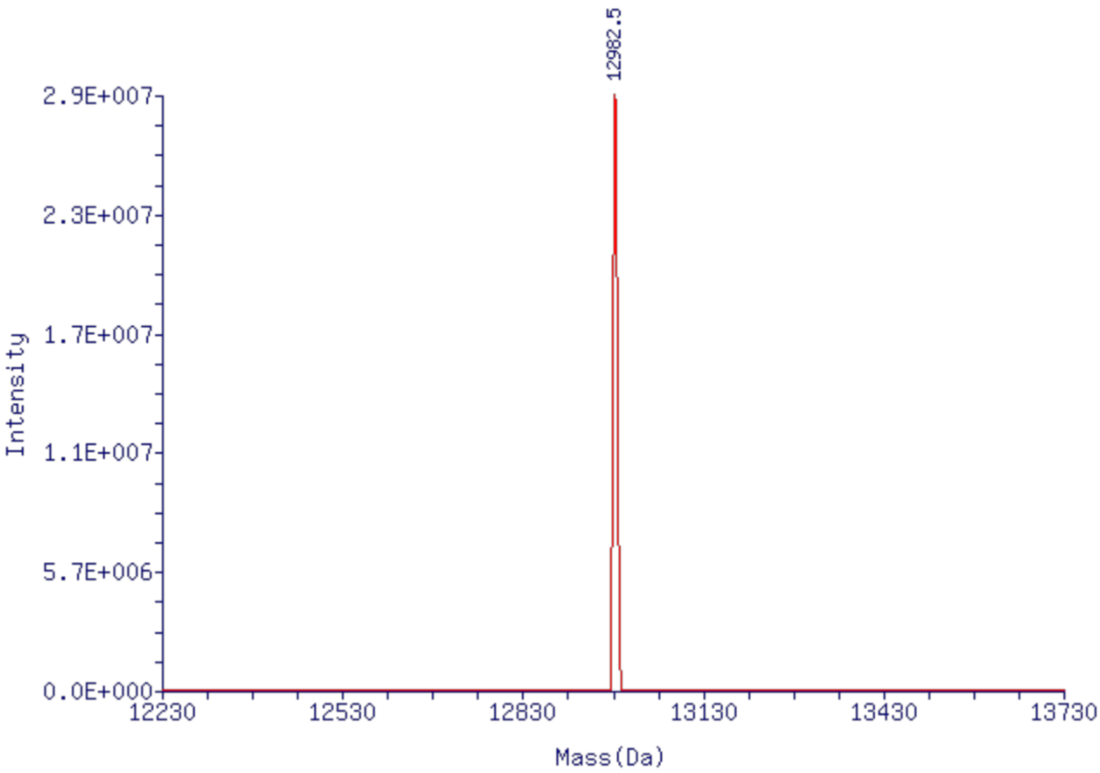

Purity: 97.5%

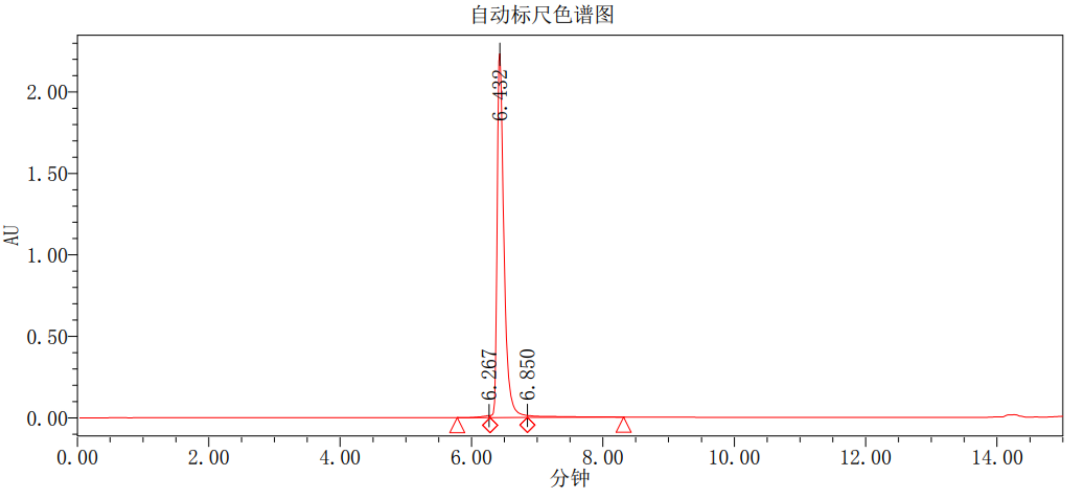

峰结果

	名称	保留时间（分钟）	面积（微伏*秒）	高度（微伏）	% 面积
1		6.267	104187	11572	0.62
2		6.432	16315359	2239170	97.53
3		6.850	309552	11797	1.85

Reference

1. Wu, Y.; Pu, C.; Fu, Y.; Dong, G.; Huang, M.; Sheng, C., NAMPT-targeting PROTAC promotes antitumor immunity *via* suppressing myeloid-derived suppressor cell expansion. *Acta Pharmaceutica Sinica B* **2022,** *12* (6), 2859-2868.

2. Li, F.; Lu, J.; Liu, J.; Liang, C.; Wang, M.; Wang, L.; Li, D.; Yao, H.; Zhang, Q.; Wen, J.; Zhang, Z.-K.; Li, J.; Lv, Q.; He, X.; Guo, B.; Guan, D.; Yu, Y.; Dang, L.; Wu, X.; Li, Y.; Chen, G.; Jiang, F.; Sun, S.; Zhang, B.-T.; Lu, A.; Zhang, G., A water-soluble nucleolin aptamer-paclitaxel conjugate for tumor-specific targeting in ovarian cancer. *Nature Communications* **2017,** *8*, 1390.

3. He, S.; Gao, F.; Ma, J.; Ma, H.; Dong, G.; Sheng, C., Aptamer-PROTAC Conjugates (APCs) for Tumor-Specific Targeting in Breast Cancer. *Angewandte Chemie-International Edition* **2021,** *60* (43), 23299-23305.

4. Chou, T.-C., Drug Combination Studies and Their Synergy Quantification Using the Chou-Talalay Method. *Cancer Research* **2010,** *70* (2), 440-446.
